# The Effect of Telehealth on Hospital Services Use: Systematic Review and Meta-analysis

**DOI:** 10.2196/25195

**Published:** 2021-09-01

**Authors:** Guido M Peters, Laura Kooij, Anke Lenferink, Wim H van Harten, Carine J M Doggen

**Affiliations:** 1 Department of Clinical Research Rijnstate Hospital Arnhem Netherlands; 2 Department of Health Technology and Services Research Technical Medical Centre University of Twente Enschede Netherlands; 3 Department of Information and Medical Technology Rijnstate Hospital Arnhem Netherlands; 4 Division of Psychosocial Research and Epidemiology Netherlands Cancer Institute Amsterdam Netherlands; 5 Rijnstate Hospital Arnhem Netherlands

**Keywords:** telehealth, systematic review, meta-analysis, hospitalization, health services use, eHealth

## Abstract

**Background:**

Telehealth interventions, that is, health care provided over a distance using information and communication technology, are suggested as a solution to rising health care costs by reducing hospital service use. However, the extent to which this is possible is unclear.

**Objective:**

The aim of this study is to evaluate the effect of telehealth on the use of hospital services, that is, (duration of) hospitalizations, and to compare the effects between telehealth types and health conditions.

**Methods:**

We searched PubMed, Scopus, and the Cochrane Library from inception until April 2019. Peer-reviewed randomized controlled trials (RCTs) reporting the effect of telehealth interventions on hospital service use compared with usual care were included. Risk of bias was assessed using the Cochrane Risk of Bias 2 tool and quality of evidence according to the Grading of Recommendations Assessment, Development and Evaluation guidelines.

**Results:**

We included 127 RCTs in the meta-analysis. Of these RCTs, 82.7% (105/127) had a low risk of bias or some concerns overall. High-quality evidence shows that telehealth reduces the risk of all-cause or condition-related hospitalization by 18 (95% CI 0-30) and 37 (95% CI 20-60) per 1000 patients, respectively. We found high-quality evidence that telehealth leads to reductions in the mean all-cause and condition-related hospitalizations, with 50 and 110 fewer hospitalizations per 1000 patients, respectively. Overall, the all-cause hospital days decreased by 1.07 (95% CI −1.76 to −0.39) days per patient. For hospitalized patients, the mean hospital stay for condition-related hospitalizations decreased by 0.89 (95% CI −1.42 to −0.36) days. The effects were similar between telehealth types and health conditions. A trend was observed for studies with longer follow-up periods yielding larger effects.

**Conclusions:**

Small to moderate reductions in hospital service use can be achieved using telehealth. It should be noted that, despite the large number of included studies, uncertainties around the magnitude of effects remain, and not all effects are statistically significant.

## Introduction

Many see the COVID-19 crisis as an opportunity to stimulate digital transformation. We can expect digital care and eHealth to receive a boost during this era. Creativity and flexibility are stimulated to formulate an answer to challenges in patients fearing infection in a hospital and to social distancing being necessary within hospital premises. Telehealth, defined as health care provided over a distance using information and communication technology (ICT) to enable interaction between patients and health professionals [1], may offer a solution. However, the efficacy of telehealth is unclear. When the dust has settled, there is a need to properly evaluate experiences and the evidence base underlying various forms of telehealth.

In addition, digital transformation is considered in response to the need to improve patient centeredness and concerns about growing health care expenditures [2,3]. Limiting the need for inpatient care, which is the main driver of hospital costs, may reduce health care expenditures [4,5]. Manufacturers’ claims and commercial pilot reports seem to dominate the debate, and policy makers frequently embrace those claims. In the Netherlands, the government presumes that hospital care can return to a very low percentage of annual volume growth in view of the anticipated effects of digital transformation. However, the extent to which telehealth can reduce hospital service use remains unclear. Some reviews have reported on the effect of telehealth on this outcome, finding both reductions and increases in hospital service use [6-8]. A recent systematic overview of telehealth interventions found that the effect on all-cause hospitalizations ranged from a reduction of 13.8% to an increase of 4.7% [6]. No prior review has compared the effects between health conditions, and most have focused on a single telehealth type, limiting generalizability [6-8]. Firm evidence for economic benefits is also limited, as cost-effectiveness studies are sparse and show contradictory results [9,10]. Moreover, telehealth can be implemented in various ways. Telehealth interventions include (1) video consultation, (2) automated device-based monitoring, (3) web-based monitoring, (4) interactive voice response (IVR) systems, (5) mobile telemonitoring, and (6) structured telephone support (STS) [6].

We conducted a systematic literature review of randomized controlled trials (RCTs) aiming to provide an overview of the evidence for the effect of telehealth on hospital services use, that is, all-cause and condition-related hospitalizations, and their duration (per patient and per hospitalization). Furthermore, we evaluated the risk of bias in all studies, as well as the quality of evidence for all outcomes. Finally, we explored which types of telehealth are most effective and which patient groups are the optimal target for reducing hospital service use.

## Methods

### Overview

This review followed the guidelines of the Cochrane Handbook, with some modifications [11]. Notably, we used reporting of the outcomes of interest as an inclusion criterion, selected studies and extracted data partially in duplicate (20%), and deviated somewhat from the suggested algorithm to judge the risk of bias arising from the randomization process (Multimedia Appendix 1).

### Data Sources and Searches

We searched MEDLINE, Scopus (Elsevier), and the Cochrane Central Register of Controlled Trials (Wiley) from inception up to April 2019. The search strategy (Multimedia Appendix 2) was developed by GMP using MeSH (Medical Subject Headings) terms and reference lists of relevant reviews until it encompassed all important keywords, and the search found all pertinent articles included in earlier reviews. WHVH and CJMD critically evaluated the search strategy before implementation.

### Eligibility Criteria

RCTs and cluster RCTs reporting the use of telehealth interventions compared with usual care were included. Telehealth was defined as health care interventions provided over a distance using ICT to enable interactions between patients and health professionals or among health professionals. Patients of any age and with any health conditions were considered. Reported outcomes included at least one of the following: all-cause hospitalization, condition-related hospitalization, or length of hospital stay. We considered only published, English, full-text, and peer reviewed articles. We did not apply any restrictions to the setting or date of publication.

This review follows the taxonomy of telehealth interventions developed in another systematic review [8], which differentiates between video consultations, (automated) device-based monitoring, web-based telemonitoring, IVR, mobile telemonitoring, and STS.

Video consultations are defined as any intervention using synchronous, two-way, audio-visual communication between patients and health care providers to perform triage or provide health advice. If measurement devices were provided, measurements were communicated solely during the video consultations.

In device-based monitoring, patients are provided with devices to measure vital signs or to report symptoms essential for detecting changes in health status. Automated alerts triggering actions from health care providers, such as phone calls, are frequently included.

Web-based telemonitoring includes interventions using a web portal to enable patients to report vital signs and symptoms, and to enable health professionals to provide educational material and feedback.

In IVR systems, patients are required to enter vital signs and symptoms through their home or mobile telephone in response to automated questions. These systems are typically combined with automated alerts that trigger actions from health care providers.

With mobile telemonitoring, patients actively submit vital signs and symptoms through their personal mobile devices. Vital signs are measured using external measurement devices.

STS provides patients with a specified number of telephone contacts for a given period of time, during which patients report their health status and receive health advice, medication adjustments, or referrals to health professionals.

We defined condition-related hospitalizations as hospitalizations due to the targeted health conditions. Studies that explicitly reported only condition-related outcomes are not aggregated with all-cause outcomes, as outcomes resulting from causes other than the condition of interest are unknown in that case, which could bias the results.

For the mean length of hospital stay, the total number of hospital days was divided by the total number of hospital stays. This is in contrast to the number of hospital days, where the total number of hospital days was divided by the total number of patients.

### Data Collection and Extraction

GMP screened all titles and abstracts. This screening was independently verified on a sample basis (10%) by LK and AL. Screening of full text articles was performed identically. Disagreements were resolved through discussion, or adjudication by CJMD. Screening was performed using the Covidence systematic review software [12].

Using a standardized data extraction form, GMP extracted the following data from all included studies: study characteristics (eg, country and setting), population characteristics (eg, health condition, age, and gender), intervention details (eg, ICT components used and frequency of use), and outcomes (hospitalizations, length of hospital stay, and hospital days; Multimedia Appendix 3). Data extraction was verified by LK on a sample basis.

### Assessment of Risk of Bias

We used the Cochrane Risk of Bias 2 (RoB 2) tool to assess the risk of bias for each study [13]. A number of rules were derived from the manual to ensure consistent judgments between reviewers (Multimedia Appendix 1). GMP assessed the risk of bias of all studies. Risk of bias assessment was performed independently and in duplicate for all studies by LK, AL, or CJMD. Disagreements were resolved through discussion or arbitration by a third reviewer, if necessary. The authors of the studies were not contacted for additional information in case of missing data or methodological unclarities.

### Data Synthesis and Analysis

Risk differences between telehealth and usual care were calculated for data reported as cumulative incidences. Cumulative incidences reported as percentages were converted to the number of participants with events. For data reported as means, such as the mean number of hospitalizations per patient, the mean differences (MDs) between telehealth and usual care were calculated. Missing SDs were calculated, where possible. All calculations were performed according to Chapter 6 of the Cochrane Handbook [14]. Meta-analyses were conducted with the meta package in R, Version 3.6.3, (R Foundation for Statistical Computing) [15], using Mantel-Haenszel random-effects models. Hartung-Knapp adjustment is used to better reflect the uncertainty in the estimation of between-study heterogeneity in CIs [16,17].

The overall quality of evidence was rated according to the Grading of Recommendations Assessment, Development and Evaluation (GRADE) approach (Multimedia Appendix 4) [18]. GMP rated the quality of evidence for each outcome (Multimedia Appendix 5). This rating was verified by all other authors, and disagreements were resolved by discussion.

We conducted subgroup analyses for health conditions that were studied in at least two articles, as well as for each type of telehealth, length of follow-up, and risk of bias. These analyses were planned a priori. The risk of bias was analyzed using the robvis package in R [19]. To assess publication bias, we visually inspected funnel plots (using the meta package in R).

## Results

### Study Selection

**Figure 1:**
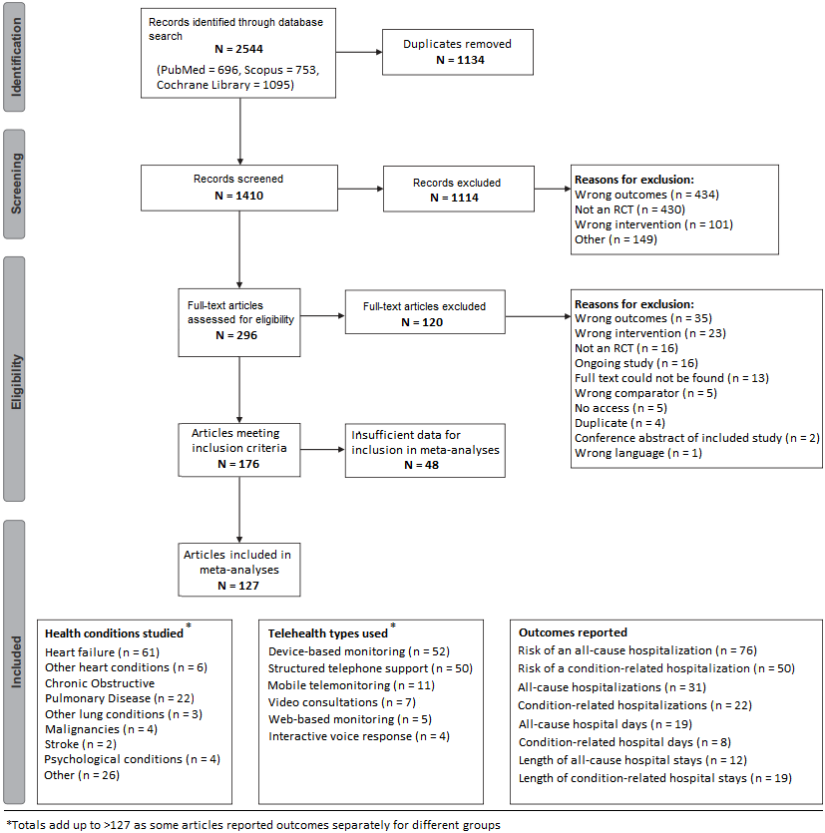
Study selection flowchart and study characteristics. RCT: randomized controlled trial.

The search identified 2544 records. After removing duplicates, 1410 records remained for the screening of titles and abstracts, through which 1114 (79.0%) records were excluded. We assessed 296 full-text articles for eligibility and excluded 120 articles. Of the remaining 176 articles, 127 (72.2%) provided sufficient data for inclusion in the meta-analysis (Multimedia Appendix 6). Figure 1 provides an overview of the study selection process.

### Study Characteristics

An overview of telehealth types, health conditions, and outcomes is provided in Figure 1 (details are provided in Multimedia Appendix 3). Most studies were conducted in Europe (n=55) and North America (n=41).

### Risk of Bias

**Figure 2:**
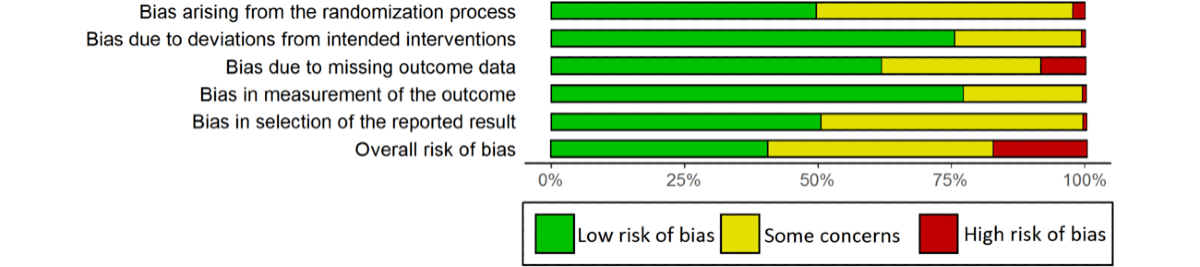
Unweighted risk of bias summary.

We judged 50 articles to be at low overall risk of bias, 55 to have some concerns, and 22 to be at high risk of bias. Most articles were assessed at low risk of bias for all five domains (64/127, 50.4% to 98/127, 77.2%), except for selection of the reported result (63/127, 49.6%; Figure 2). High risk was found for bias arising from the randomization process in only 3 articles, bias due to deviations from intended interventions in one, due to missing outcome data in 11, bias in measurement of the outcome in one, and in selection of the reported result in 1 out of 127 articles. Weighted risk of bias summaries are provided for each analysis in Multimedia Appendix 5. In the analyses of condition-related hospitalizations and the length of hospital stay due to any cause, studies at high risk of bias in at least one domain cumulatively accounted for approximately 20% of the weight. In all other analyses, this figure was below 10%.

### Outcomes

The summary of findings table (Table 1) provides a comprehensive overview of the main results for all outcomes.

For each analysis, most RCTs used device-based monitoring or STS and included mainly patients with heart failure or chronic obstructive pulmonary disease (COPD; details Multimedia Appendix 3). Complete analyses are available in Multimedia Appendix 5.

The outcomes are reported as rates in 14 articles. Although these could not be incorporated in the meta-analyses, an overview of these results is provided in Multimedia Appendix 7.

**Table 1:** Summary of findings table for the effect of telehealth interventions on various outcome measures compared with usual care.

Outcome	Studies (RCTs^a^), n	Participants, n	Follow-up (months)	Usual care estimate	Intervention effect estimate	Effect estimate (95% CI)	GRADE^b^ Strength of evidence^c^	Plain language summary	
Patients with an all-cause hospitalization (patients hospitalized per 1000 patients)	76	34,423	1-60	373	355	Risk difference: −18 (−30 to −0)	High	The number of patients hospitalized for any cause is reduced by 4.8%^d^	
Patients with a condition-related hospitalization (hospitalizations per 1000 patients)	50	20,867	1-60	237	200	Risk difference: −37 (−60 to −20)	High	The number of patients hospitalized for the condition targeted is reduced by 15.6%^d^	
Mean all-cause hospitalizations per patient (hospitalizations per 1000 patients)	31	11,191	3-12	880	830	Mean difference: −50 (−140 to +30)	High	All-cause hospitalizations are reduced by 5.7%^d^	
Mean condition-related hospitalizations per patient (hospitalizations per 1000 patients)	22	3461	1-60	470	360	Mean difference: −110 (−200 to −10)	High	Condition-related hospitalizations are reduced by 23.4%^d^	
All-cause hospital days^e^ (hospital days per patient)	19	9735	0-60	6.06	4.99	Mean difference: −1.07 (−1.76 to −0.39)	High	The mean number of days spent in the hospital for any cause per patient is reduced by 17.7%^d^	
Condition-related hospital days^e^ (hospital days per patient)	8	1216	3-60	2.84	1.71	Mean difference: −1.13 (−1.64 to −0.61)	Moderate^f^	The mean number of days spent in the hospital for the condition targeted is reduced by 39.8%^d^	
Length of all-cause hospital stay^g^ (days per hospitalization)	12	1964	0-60	8.37	7.89	Mean difference: −0.48 (−1.50 to 0.53)	Low^h^	Hospitalizations for any cause are 5.7%^d^ shorter with telehealth	
Condition-related hospital length of stay^g^ (days per hospital stay)	15	2047	0-24	2.92	2.03	Mean difference: −0.89 (−1.42 to −0.36)	High	Hospitalizations for the condition targeted are 30.5%^d^ shorter with telehealth	

^a^RCT: randomized controlled trial.

^b^GRADE: Grading of Recommendations Assessment, Development and Evaluation.

^c^High: we are very confident that the true effect lies close to that of the estimate of the effect; moderate: we are moderately confident in the effect estimate: the true effect is likely to be close to the estimate of the effect, but there is a possibility that it is substantially different; low: our confidence in the effect estimate is limited: the true effect may be substantially different from the estimate of the effect.

^d^Percentages were calculated by dividing the effect estimate by the usual care estimate.

^e^Participants are the unit of analysis.

^f^Downgraded by one level for risk of publication bias.

^g^Hospitalizations are the unit of analysis.

^h^Downgraded by one level for risk of bias and another for imprecision.

### Risk of All-Cause Hospitalization

The risk of all-cause hospitalization was reported by 76 RCTs, including 34,423 participants. The analysis provides high-quality evidence for a risk difference of −18 (95% CI −30 to 0) hospitalized patients per 1000 patients (−4.8% of usual care).

### Risk of Condition-Related Hospitalization

We found 50 RCTs reporting the risk of condition-related hospitalization, including 20,867 participants. The absolute risk was reduced by 37 per 1000 patients (95% CI 20-60), with high-quality evidence (−5.7% of usual care). When stratified by health condition, only the heart failure group showed a statistically significant effect (risk difference = −0.03), although the subgroup difference was not significant (*P*=.40).

### Mean All-Cause Hospitalizations

We found 31 RCTs reporting the mean number of all-cause hospitalizations per patient, including 11,191 participants. Follow-up varied between 3 and 12 months. The analysis showed high-quality evidence for an MD of −50 (95% CI −140 to +30) hospitalizations per 1000 patients, a 5.7% reduction with regards to the number of hospitalizations in the usual care group. Only the COPD subgroup showed a statistically significant MD between telehealth and usual care of −200 (95% CI −390 to −10) hospitalizations per 1000 patients. No effects were found for heart failure and other diseases. In addition, an RCT studying malignancies reported an MD of +0.09 hospitalizations per patient compared with usual care but did not report a SD and was therefore excluded from the meta-analysis.

### Mean Condition-Related Hospitalizations

The mean number of condition-related hospitalizations per patient was reported in 22 RCTs, including 3461 participants. Follow-up varied between 1 and 60 months. The analysis showed high-quality evidence for an MD of −110 (−200 to −10; −23.4% of usual care) hospitalizations per 1000 patients with telehealth compared with usual care. Differences between outcomes appeared to depend on the length of follow-up (*P<*.01). The difference increased gradually with a longer follow-up from an MD of −90 between 3 and 6 months up to a reduction of 1190 hospitalizations per 1000 patients for outcomes reported after more than 12 months. When stratified by health condition, only heart failure showed a statistically significant effect (MD −120; −200 to −40 hospitalizations per 1000 patients).

### All-Cause Hospital Days

The mean number of days patients were hospitalized for any cause was reported in 19 RCTs including 9735 participants. Overall, the analysis showed high quality evidence for an MD of −1.07 (95% CI −1.76 to −0.39) hospital days per patient. In addition, 9 RCTs reported the total number of days for which patients were hospitalized, and 2 reported the rate of hospital days. Furthermore, 1 RCT reported an MD of +0.60 hospital days with telehealth compared with usual care but did not report an SD nor the necessary information to calculate one. These 12 RCTs, which included 3144 participants, could not be incorporated in the meta-analysis.

### Condition-Related Hospital Days

The mean number of days patients were hospitalized for the condition of interest was reported by 8 RCTs, including a total of 1216 participants. The analysis showed moderate quality evidence of an MD of −1.13 (95% CI −1.64 to −0.61) hospital days per patient. The quality of evidence was downgraded because of risk of publication bias. A statistically significant difference was found for the length of follow-up (*P*<.01), with longer follow-up resulting in larger reductions in hospital days. It is notable that when stratified by health condition, a statistically significant result was only achieved in heart failure (MD −1.06 hospital days, 95% CI −1.71 to −0.40). For COPD, an MD of −1.75 (95% CI −4.62 to 1.11) was found. In addition, 7 studies reported the total number of days patients were hospitalized, and one reported the rate of hospital days. These studies, including 2492 participants, could not be included in the meta-analysis.

### Length of All-Cause Hospital Stay for Hospitalized Patients

A total of 12 RCTs reported length of all-cause hospital stay, including 1964 hospitalized patients. Low-quality evidence was found for an MD of −0.48 (95% CI −1.44 to +0.47 days) hospital days per stay. The quality of evidence was downgraded by one level for risk of bias and by another for imprecision. Subgroup differences were found between different lengths of follow-up (*P*<.01) and different levels of risk of bias (*P*≤.01), but no clear trends were found. Three studies reported the length of hospital stay as medians and IQRs, and they could therefore not be included in the meta-analysis.

### Length of Condition-Related Hospital Stay for Hospitalized Patients

Fifteen RCTs reported length of condition-related hospital stay, including 2047 hospitalized patients. The analysis showed high-quality evidence for an MD of −0.89 hospital days per stay (95% CI −1.42 to −0.36 days).

Subgroup differences were found in reporting outcomes at different lengths of follow-up (*P<*.01). An MD of −3.95 hospital days per stay (95% CI −6.06 to −1.84 days) was found for reporting between 7 and 12 months, whereas other MDs ranged from −1.00 to −0.42 days. An additional 3 RCTs reported the length of hospital stay as medians and IQRs and 4 did not report SDs nor any information that could be used to calculate them. These 7 RCTs, including 922 participants, were therefore excluded from the meta-analysis.

## Discussion

### Principal Findings

Our review indicates that the risk of all-cause hospitalization decreased significantly by 18 hospitalizations per 1000 patients (−4.8%) and 37 (−15.6%) for condition-related hospitalizations. We found high-quality evidence that, compared with usual care, telehealth leads to reductions in mean all-cause (MD −0.05, 95% CI −0.14 to 0.03 hospitalizations per patient; −5.7% of usual care) and condition-related hospitalizations (MD −0.11, 95% −0.20 to −0.01; −23.4%), that is, 50 to 110 fewer mean hospitalizations, respectively, per 1000 patients. Overall, it is evident that all-cause hospital days decreased significantly with a mean of −1.07 (−17.7%) hospital days per patient and condition-related hospital days with −1.13 (−39.8%) days, although evidence for the latter was only moderate. For hospitalized patients, the mean stay for any cause could potentially be reduced (MD −0.48 days, 95% CI −1.50 to 0.53; 5.7%, low-quality evidence), and mean stay for condition-related hospitalizations even more (MD −0.89 days, 95% CI −1.42 to −0.36; 30.5%, high-quality evidence). The effects were similar for various health conditions and types of telehealth. A trend was observed for studies with longer follow-up periods, yielding larger effects. It should, however, be noted that, although this is a systematic review including a large number of studies, uncertainties around the magnitude of effects remain, and not all differences were statistically significant.

The quality of evidence was high for most of the analyses. Downgrading was only necessary for two analyses because of the risk of bias, risk of publication bias, and imprecision because of a small cumulative sample size. Overall, there were approximately as many articles with some concerns as there were articles at low risk of bias. The main culprits were insufficient reporting of the randomization method, lack of available trial registrations or study protocols, and incomplete outcome data (mostly due to deaths). None of these aspects necessarily indicate issues with the study itself, but rather with the reporting of a study. It is desirable that more information is made available, such as by providing web-based supplementary material.

### Comparison With Prior Work

In our review, the most commonly used telehealth types were device-based monitoring and STS. In general, only small differences in effects were found between telehealth types, which did not appear to be relevant. This finding is in line with a Cochrane review including RCTs investigating the effect of either STS or device-based monitoring in the management of heart failure, which also found no difference [20]. It should be explored whether design aspects, such as monitoring frequency or duration, or patient engagement, could explain the differences in effect. Furthermore, patient compliance is often important for the success of telehealth interventions. For example, the patients must consistently take and send measurements, be available for telephone contacts or video consultations, or report symptoms. If these actions are not taken by the patient, telehealth interventions cannot function. Therefore, it is important to consider patient preferences during the design process [21,22].

Studies including patients with heart failure or COPD accounted for the majority of the weight in the meta-analyses of this review, although the effects found for other health conditions seemed similar. No other review has combined the results for multiple health conditions. However, reviews of heart failure and COPD specifically are available for comparison. A systematic review including reviews on telehealth for chronic heart failure patients published between 1996 and 2014 found low-quality evidence for absolute risk reductions in patients with an all-cause hospitalization of 4.7% to 13.8% and of 3.7% to 8.2% for patients with a condition-related hospitalization [6]. Our estimate for patients with all-cause hospitalization was considerably lower (2%) and more precise. This is caused by the larger number of studies (75 in our study vs 8 in the other meta-analysis) and thus participants in our analysis (N=30,937 vs N=2343). Our estimate for patients with condition-related hospitalization was similar (3.8%). A recent review on telehealth for heart failure patients also found a trend toward reduced hospitalizations [23]. Another recent review, on coronary heart disease patients, found a relative risk of 0.56 (95% CI 0.39-0.81), although absolute differences were also small [24].

A systematic overview of reviews including COPD patients found 3 reviews investigating the effect of telehealth on hospitalizations, all of which found a reduction in hospitalizations [7]. Another systematic review reported reduced hospitalizations in 8 out of 11 studies, ranging from −10% to −63%. The findings were similar for all-cause hospitalization and condition-related hospitalizations [25]. Our review confirms the reduction in hospitalizations also found in previous reviews and provides a more realistic estimate of the effect through meta-analyses, which was rarely performed in previous reviews.

In a systematic overview of the use of telehealth for various chronic health conditions, reviews on health conditions other than heart failure or COPD also found only a few articles, except for diabetes [8]. This result is consistent with the findings of our review. As COPD and heart failure only make up a small part of the care provided by hospitals [26], more research is necessary on the effect of telehealth on hospital services use in health conditions other than COPD and heart failure, which are also highly prevalent.

The length of follow-up seems to be an important factor influencing the effect of telehealth in our review. We found subgroup differences in length of hospital stay (both all-cause and condition-related), condition-related hospitalizations, and condition-related hospital days, with larger effect sizes for studies with longer follow-up. A similar trend was observed for all-cause hospital days. One review reported a reduction in mortality at 6 months, with no differences at 1 year [21]. No other reviews assessed differences in effects between the lengths of follow-up.

When telehealth replaces face-to-face contact, it is clear that this can aid in reducing outpatient contacts and supporting social distancing in outpatient departments. In view of the small effects on hospitalizations and moderate effects on hospital inpatient days, it is important to determine whether telehealth actually contributes to cost reduction. Telehealth comes at a cost, for example, because health professionals make phone calls, conduct video consultations, or interpret data. To reduce the costs of interventions, automation of some of these aspects, for example, by developing algorithms to recognize deterioration of patients' health status, should be studied. Although we investigated whether the mechanism by which telehealth is often claimed to reduce costs is indeed present, we did not directly investigate whether costs were reduced. Thorough budget impact and cost-effectiveness studies are needed to reach firm conclusions in this domain.

### Limitations

This review has several strengths and limitations. First, the wide scope enabled us to find a large number of articles meeting our inclusion criteria. Furthermore, we quantitatively compared the effects achieved in different health conditions using different types of telehealth and length of follow-up. Another important strength is that we assessed all included articles for risk of bias and graded the strength of evidence for each analysis, providing a comprehensive overview of the evidence on the effect of telehealth on hospital service use.

The wide scope also acts as a double-edged sword in that it makes the participants in the various studies less comparable than in a typical review. This concern is alleviated by the fact that we did not find significant differences between health conditions or types of telehealth, although for some comparisons only a few studies were available. Telehealth interventions often entail many more changes to the health care process, besides the application of technology [27]. The effect of the telehealth type thus becomes entangled with the effects of changes to processes and infrastructure, which requires a more detailed analysis to unravel. Study selection was performed partially in duplicate, which may have caused some articles to have been missed. As we only included peer reviewed articles published in English, it is unknown what evidence exists in other languages. This review is further limited by our scope, which focuses on types of telehealth requiring interaction between patients and health professionals. Passive forms of digital health care, such as self-management applications or health information provision, were not included. These types of services could reduce hospital service use [28], while potentially being more efficient in terms of resource use because of their passive nature. Furthermore, we did not contact the study authors for details in the case of missing data or methodological unclarities.

### Conclusions

Thus, the effects of telehealth are small to moderate and appear to be stronger for condition-related outcomes than for all-cause outcomes. Further research is needed to obtain more insight into the effects of telehealth on other diseases, apart from COPD and heart failure, and into which aspects of telehealth interventions result in positive effects.

Finally, in the context of the COVID-19 crisis, it is important to acknowledge that a great deal of health care can be provided from a distance, eliminating the need for vulnerable individuals to come to a potentially hazardous environment to receive health care and enabling hospitals to continue providing care to all who need it.

## Appendix

Multimedia Appendix 1 Deviation from and clarification of Cochrane Risk of Bias 2 Tool guidance document.

Multimedia Appendix 2 Search syntaxes.

Multimedia Appendix 3 Study characteristics.

Multimedia Appendix 4 Grading of Recommendations Assessment, Development and Evaluation protocol.

Multimedia Appendix 5 Complete analyses and Grading of Recommendations Assessment, Development and Evaluation assessments.

Multimedia Appendix 6 Studies included in the meta-analysis.

Multimedia Appendix 7 Hospitalization rates.

## Abbreviations

COPD: chronic obstructive pulmonary disease
GRADE: Grading of Recommendations Assessment, Development and Evaluation
ICT: information and communication technology
IVR: interactive voice response
MD: mean difference
MeSH: Medical Subject Headings
RCT: randomized controlled trial
STS: structured telephone support
