# Supplementary material for: The Effect of Telehealth on Hospital Services Use: Systematic Review and Meta-analysis
Source: J Med Internet Res. 2021 Sep 1;23(9):e25195. doi: 10.2196/25195 (PMC8444037; doi:10.2196/25195)
Supplement: Multimedia Appendix 1 [file jmir_v23i9e25195_app1.docx]

**Multimedia Appendix 1: Deviation from and clarification of the Cochrane Risk of Bias 2 Tool guidance document**

**Deviation**

Randomization: The algorithm suggested by the Cochrane Risk of Bias 2 guidance document immediately judges randomization to be at high risk of bias if the next allocation could have been known (e.g. due to a systematic allocation method or small block sizes). We, instead, assessed randomization as “Some concerns” if this was the case, but there were no relevant differences in baseline characteristics between groups. We only assessed randomization as high risk of bias if it was clear that none of the components met the criteria proposed in the manual, or important differences in baseline characteristics were observed.

**Clarification**

Selective outcomes reporting: If no trial registration or study protocol was available to check whether the outcomes of interest for our review were planned for analysis, the default judgement for this outcome was “Some concerns”. If a trial registration or study protocol *was* available, it had to be checked whether the outcomes of interest for our review were indeed planned a priori. If that was the case, our judgement was “Low risk”. If hospital services use was not mentioned in the trial registration or study protocol, but was reported as a secondary outcome in the article, our judgement was “Some concerns”. If these outcomes were not planned according to the trial registration or study protocol, but were reported in the article as primary outcome measure, our judgement was “High risk”. If they were planned as a secondary outcome measure, but reported as primary outcome in the article, our judgement was also “High risk”.
