# Supplementary material for: The Effect of Telehealth on Hospital Services Use: Systematic Review and Meta-analysis
Source: J Med Internet Res. 2021 Sep 1;23(9):e25195. doi: 10.2196/25195 (PMC8444037; doi:10.2196/25195)
Supplement: Multimedia Appendix 2 [file jmir_v23i9e25195_app2.docx]

**Multimedia Appendix 2: Search syntaxes for PubMed, Scopus, and the Cochrane Library (CENTRAL)**

## PubMed

## ((((((((((((((((((((((((telehomecare[All Fields] OR "remote consultation"[All Fields]) OR (remote[All Fields] AND ("referral and consultation"[MeSH Terms] OR ("referral"[All Fields] AND "consultation"[All Fields]) OR "referral and consultation"[All Fields] OR "consultation"[All Fields]))) OR ("remote consultation"[MeSH Terms] OR ("remote"[All Fields] AND "consultation"[All Fields]) OR "remote consultation"[All Fields] OR "teleconsultation"[All Fields])) OR "telephone follow-up"[All Fields]) OR "telephone followup"[All Fields]) OR "telephone case management"[All Fields]) OR "telephone case-management"[All Fields]) OR "telerehabilitation"[MeSH Terms]) OR ("telerehabilitation"[MeSH Terms] OR "telerehabilitation"[All Fields])) OR "telemedicine"[MeSH Terms]) OR ("telemedicine"[MeSH Terms] OR "telemedicine"[All Fields])) OR ("telemedicine"[MeSH Terms] OR "telemedicine"[All Fields] OR "ehealth"[All Fields])) OR e-health[All Fields]) OR "videoconferencing"[MeSH Terms]) OR ("videoconferencing"[MeSH Terms] OR "videoconferencing"[All Fields])) OR ("telemedicine"[MeSH Terms] OR "telemedicine"[All Fields] OR "telehealth"[All Fields])) OR telehealthcare[All Fields]) OR "home telemonitoring"[All Fields]) OR telemonitoring[All Fields]) OR ("remote sensing technology"[MeSH Terms] NOT "satellite imagery"[MeSH Terms])) OR "wireless technology"[MeSH Terms]) OR "wearable electronic devices"[MeSH Terms]) OR ((((("health"[MeSH Terms] OR "health"[All Fields]) AND care[All Fields]) OR "health care"[All Fields]) OR care[All Fields]) AND (("internet based"[All Fields] OR "computer based"[All Fields]) OR "phone based"[All Fields]))) AND (((("patients"[MeSH Terms] OR "patients"[All Fields]) OR "patient"[All Fields]) AND (((rehospitalization[All Fields] OR rehospitalisation[All Fields]) OR re-hospitalization[All Fields]) OR re-hospitalisation[All Fields])) OR (((("hospitalization"[MeSH Terms] OR "hospitalization"[All Fields]) OR "hospitalisation"[All Fields]) OR ("patient readmission"[MeSH Terms] OR readmission[All Fields])) OR ((("length of stay"[MeSH Terms] OR "length of stay"[All Fields]) OR "stay length"[All Fields]) OR "hospital stay"[All Fields])))) AND (Randomized Controlled Trial[ptyp] OR ((RCT[All Fields] OR "randomized controlled trial"[All Fields]) OR "randomised controlled trial"[All Fields])) NOT protocol[All Fields]

## Scopus

## ( ( ( ( TITLE-ABS-KEY ( telehomecare ) ) OR ( TITLE-ABS-KEY ( "remote consultation" ) ) OR ( ( TITLE-ABS-KEY ( remote ) AND TITLE-ABS-KEY ( consultation ) ) ) OR ( TITLE-ABS-KEY ( teleconsultation ) ) OR ( TITLE-ABS-KEY ( "telephone follow-up" ) ) OR ( TITLE-ABS-KEY ( "telephone follow up" ) ) OR ( TITLE-ABS-KEY ( telerehabilitation ) ) OR ( TITLE-ABS-KEY ( telemedicine ) ) OR ( TITLE-ABS-KEY ( ehealth ) ) OR ( TITLE-ABS-KEY ( "e health" ) ) OR ( TITLE-ABS-KEY ( mhealth ) ) OR ( TITLE-ABS-KEY ( "m-health" ) ) OR ( TITLE-ABS-KEY ( videoconferencing ) ) OR ( TITLE-ABS-KEY ( telehealth ) ) OR ( TITLE-ABS-KEY ( telehealthcare ) ) OR ( TITLE-ABS-KEY ( "home telemonitoring" ) ) OR ( TITLE-ABS-KEY ( telemonitoring ) ) ) OR ( ( ( ( TITLE-ABS-KEY ( health ) AND TITLE-ABS-KEY ( care ) ) ) OR ( TITLE-ABS-KEY ( "health care" ) ) OR ( TITLE-ABS-KEY ( care ) ) ) AND ( ( TITLE-ABS-KEY ( "internet based" ) ) OR ( TITLE-ABS-KEY ( "computer based" ) ) OR ( TITLE-ABS-KEY ( "phone based" ) ) ) ) ) AND ( ( TITLE-ABS-KEY ( rehospitalization ) ) OR ( TITLE-ABS-KEY ( rehospitalisation ) ) OR ( TITLE-ABS-KEY ( re-hospitalisation ) ) OR ( TITLE-ABS-KEY ( readmission ) ) OR ( TITLE-ABS-KEY ( re-admission ) ) OR ( TITLE-ABS-KEY ( hospitalization ) ) OR ( TITLE-ABS-KEY ( "length of stay" ) ) OR ( TITLE-ABS-KEY ( "stay length" ) ) OR ( TITLE-ABS-KEY ( "hospital stay" ) ) ) ) AND ( ( TITLE-ABS-KEY ( rct ) ) OR ( TITLE-ABS-KEY ( "randomized controlled trial" ) ) OR ( TITLE-ABS-KEY ( "randomised controlled trial" ) ) )

## Cochrane Library Trials (CENTRAL)

ID Search Hits

#1 (telehomecare):ti,ab,kw (Word variations have been searched) 26

#2 "remote consultation" 385

#3 (remote) AND consultation 646

#4 teleconsultation 591

#5 "telephone follow-up" 1119

#6 "telephone followup" 15

#7 "telephone case management" 13

#8 telerehabilitation 417

#9 MeSH descriptor: [Telerehabilitation] explode all trees 74

#10 telemedicine 3190

#11 MeSH descriptor: [Telemedicine] explode all trees 2044

#12 ehealth 999

#13 e-health 5549

#14 videoconferencing 559

#15 MeSH descriptor: [Videoconferencing] explode all trees 178

#16 telehealth 1143

#17 telehealthcare 29

#18 "home telemonitoring" 148

#19 telemonitoring 854

#20 MeSH descriptor: [Remote Sensing Technology] 1 tree(s) exploded 29

#21 MeSH descriptor: [Wireless Technology] explode all trees 33

#22 MeSH descriptor: [Wearable Electronic Devices] explode all trees 314

#23 #1 OR #2 OR #3 OR #4 OR #5 OR #6 OR #7 OR #8 OR #9 OR #10 OR #11 OR #12 OR #13 OR #14 OR #15 OR #16 OR #17 OR #18 OR #19 OR #20 OR #21 OR #22 12119

#24 (health) AND care 105320

#25 "health care" 56537

#26 care 226606

#27 #24 OR #25 OR #26 226606

#28 "internet based" 2907

#29 "computer based" 2419

#30 "phone based" 536

#31 #28 OR #29 OR #30 5751

#32 #27 AND #31 2428

#33 #23 OR #32 14026

#34 rehospitalization 1460

#35 rehospitalisation 359

#36 re-hospitalization 523

#37 re-hospitalisation 525

#38 MeSH descriptor: [Patient Readmission] explode all trees 922

#39 MeSH descriptor: [Length of Stay] explode all trees 6694

#40 "length of stay" 18062

#41 "stay length" 315

#42 "hospital stay" 17639

#43 MeSH descriptor: [Hospitalization] explode all trees 12870

#44 hospitalization 35929

#45 re-admission 579

#46 #34 OR #35 OR #36 OR #37 OR #38 OR #39 OR #40 OR #41 OR #42 OR #43 OR #44 OR # 45 164544

#47 #33 AND #46 5633

#48 "RCT" 27981

#49 "randomized controlled trial" 778363

#50 MeSH descriptor: [Randomized Controlled Trial] explode all trees 126

#51 #48 OR #49 OR #50 785408

#52 #47 AND #51 4690
