# Supplementary material for: The Effect of Telehealth on Hospital Services Use: Systematic Review and Meta-analysis
Source: J Med Internet Res. 2021 Sep 1;23(9):e25195. doi: 10.2196/25195 (PMC8444037; doi:10.2196/25195)
Supplement: Multimedia Appendix 3 [file jmir_v23i9e25195_app3.docx]

**Multimedia Appendix 3: Characteristics of studies included in the meta-analyses**

| Author, year | Sponsorship source | Country | Setting | Health condition | Telehealth type | Usual care |
| --- | --- | --- | --- | --- | --- | --- |
| Abraham 2011 ^1^ | CardioMEMS | USA | Hospital, not further specified | Heart failure | Device-based monitoring | Also received usual care |
| Al-Sutari 2017 ^2^ | None declared | Jordan | Teaching hospital | Heart failure | Structured telephone support | n.a. |
| Amara 2017 ^3^ | Biotronik SE & Co. | France | Hospital, multicentre | Supraventricular arrhythmia | Device-based monitoring | Ambulatory visits at 1-3 months and 12 months. |
| Angermann 2012 ^4^ | University of Wuerzburg. | Germany | Hospital, multicentre | Heart failure | Structured telephone support | Treatment plans, comprehensive discharge letters, appointment with GP or cardiologist within 7-14 days. |
| Antoniades 2012 ^5^ | Austin Hospital | Australia | Metropolitan Hospital | COPD | Device-based monitoring | Adherence to established guidelines, assessment by trained respiratory nurse, COPD education, social work, occupational therapy, close post-discharge follow-up with access to outreach nursing, assistance in developing self-management plan. |
| Arendts 2018 ^6^ | ^5^State Health Research Advisory Council of Western Australia | Australia | Hospital | n.a. | Structured telephone support | n.a. |
| Basch 2016 ^7^ | National Cancer Institute, Memorial Sloan Kettering Cancer Center | USA | Hospital | Cancer | Web-based monitoring | n.a. |
| Bekelman 2015 ^8^ | Veterans Affairs | USA | Hospital | Heart failure | Interactive voice response | Information sheets outlining self-care, and care at discretion of regular VA provider, potentially including cardiology specialty care, CHF education, etc. |
| Bell 2015 ^9^ | Vanderbilt University Medical Center; National Heart, Lung, and Blood Institute (NHLBI) | USA | Academic hospital | Heart failure | Structured telephone support | n.a. |
| Biese 2018 ^10^ | Duke Endowment; the Kenan Family Foundation; Mr. John A. McNeill, Jr. | USA | Hospital | n.a. | Structured telephone support | n.a. |
| Bohingamu Mudiyanselage 2018 ^11^ | Victorian Government; Barwon Health | Australia | Community | Mixed | Device-based monitoring | n.a. |
| Böhm 2016 ^12^ | Medtronic PLC | Germany | Hospital | Heart failure | Device-based monitoring | n.a. |
| Bonetti 2018 ^13^ | Universidade Federal do ParanÃ¡ | Brazil | Hospital | Cardiovascular disease | Structured telephone support | No post-discharge care. |
| Boriani 2013 ^14^ | Medtronic Bakken Research Center | Italy | Hospital | Heart failure | Device-based monitoring | In-office visits at baseline and 8 months. |
| Boriani 2017 ^15^ | Medtronic | Multinational | Hospital | Heart failure | Device-based monitoring | n.a. |
| Bourbeau 2003 ^16^ | Boehringer Ingelheim Canada; Fonds de la Recherche en SantÃ© du QuÃ©bec. | Canada | Hospital | COPD | Structured telephone support | n.a. |
| Bowles 2009 ^17^ | Centers for Disease Control and Prevention | USA | Community | Heart failure or diabetes | Structured telephone support | Home nursing according to evidence-based disease-management protocol. |
| Bowles 2009b ^17^ | Centers for Disease Control and Prevention | USA | Community | Heart failure or diabetes | Device-based monitoring | Home nursing according to evidence-based disease-management protocol. |
| Bowles 2011 ^18^ | National Institute of Nursing Research | USA | Community | Heart failure | Device-based monitoring | Home visits. |
| Boyne 2012 ^19^ | The Province of Limburg in The Netherlands; the Annadal Foundation Maastricht; Astra Zeneca [an unrestricted grant]; the Rescar Foundation Maastricht | The Netherlands | Hospital | Heart failure | Interactive voice response | Two fewer follow-up visits than the usual care group. |
| Braun 2009 ^20^ | Not reported | Israel | Hospital | Miscellaneous | Structured telephone support | Patients receive a \Discharge Report\" including patient history |
| Chau 2012 ^21^ | Not reported. | Hong Kong | Hospital | COPD | Device-based monitoring | Home visits from the community nurse educating patients on use of medication, purse-lip breathing, lifestyle modification, and exercise |
| Chaudhry 2010 ^22^ | National Heart Blood and Lung Institute | USA | Hospital | Heart failure | Interactive voice response | n.a. |
| Chen 2011 ^23^ | Chang Gung Memorial Hospital | Taiwan | Hospital | Chronic Kidney Disease | Structured telephone support | n.a. |
| Chen 2019 ^24^ | Not reported | China | Hospital | Heart failure | Structured telephone support | Single educational session before discharge. |
| Chiantera 2005 ^25^ | Not reported | Italy | Hospital | Acute coronary syndrome | Device-based monitoring | n.a. |
| Cleland 2005 ^26^ | Not reported. | United Kingdom | Hospital | Heart failure | Device-based monitoring | n.a. |
| Comin-Colet 2016 ^27^ | Telefonica Soluciones S.A; IMIM. | Spain | Hospital | Heart failure | Device-based monitoring | n.a. |
| Dansky 2008 ^28^ | Robert Wood Johnson Foundation | USA | NA | Heart failure | Device-based monitoring | Routine home visits. |
| Dar 2009 ^29^ | Honeywell HomMed | UK | Hospital | Heart failure | Device-based monitoring | Initial home visit by study nurse. Regular clinic review, including life-style advice and medication optimalization. |
| Datta 2010 ^30^ | US Department of Veterans Affairs | USA | Primary care | Mixed | Structured telephone support | Phone contacts at 6 and 24 months to collect secondary outcome data. |
| De Jong 2017 ^31^ | Maastricht University Medical Centre | The Netherlands | Hospital | IBD | Web-based monitoring | At least one scheduled outpatient visit per year. |
| Dendale 2012 ^32^ | The Belgian Government Health Insurance Institute | Belgium | Primary care | Heart failure | Device-based monitoring | Standard one hour education course. Outpatient follow up after 2 weeks. Planned in-patient clinic follow-up at 3 and 6 months. |
| De San Miguel 2013 ^33^ | Australian Department of Health and Aging | Australia | Community | COPD | Device-based monitoring | COPD book. |
| De Vito Dabbs 2016 ^34^ | National Institute of Nursing Research | USA | Academic Hospital | Lung transplant | Mobile telemonitoring | Scripted discharge instructions of 60 minutes, and an instruction binder. |
| DeWalt 2006 ^35^ | Pfizer Health Literacy Initiative; the Robert Wood Johnson Clinical Scholars Program; the University of North Carolina Program on Health Outcomes; the National Institute of Nursing Research, NIH | USA | Hospital | Heart failure | Structured telephone support | Heart failure education pamphlet written at 7th grade level and usual care from primary physician. |
| Dhalla 2014 ^36^ | Canadian Institutes of Health Research; the Ontario Ministry of Health and Long-Term Care; the Green Shield Canada Foundation; the University of Toronto Department of Medicine; the Academic Funding Plan Innovation Fund. | Canada | Hospital | Miscellaneous | Structured telephone support | n.a. |
| Dinesen 2012 ^37^ | Bureau of Business and Construction | Denmark | Hospital | COPD | Device-based monitoring | Home exercises and contacting GP or emergency doctor when needed. |
| Domingues 2011 ^38^ | FundaÃ§Ã£o Instituto de Pesquisas EconÃ´micas; Conselho Nacional de Desenvolvimento CientÃ­fico e TecnolÃ³gico. | Brazil | Hospital | Heart failure | Structured telephone support | n.a. |
| Dougherty 2005 ^39^ | National Institutes of Health, National Institute for Nursing Research | USA | Hospital | Sudden cardiac arrest | Structured telephone support | Standardized hospital-based education (booklets and videos developed by the ICD manufacturer) and outpatient clinic visits. |
| Dudas 2002 ^40^ | University of California, San Francisco, Department of Medicine RESPECT grant program. | USA | Hospital | Mixed | Structured telephone support | n.a. |
| Ferrante 2010 ^41^ | Not explicitly reported; GESICA Foundation implied | Argentina | Hospital | Heart failure | Structured telephone support | n.a. |
| Finlayson 2018 ^42^ | Australian Research Council Discovery Project Grants Scheme | Australia | Hospital | Miscellaneous | Structured telephone support | Routine discharge planning, rehabilitation advice, and potentially community nursing. |
| Fors 2018 ^43^ | Centre for Person-Centred Care, University of Gothenburg | Sweden | Academic hospital | Mixed | Structured telephone support | According to guidelines |
| Gallagher 2017 ^44^ | Columbia University | USA | Academic hospital | Heart failure | Device-based monitoring | Medication |
| Garbutt 2010 ^45^ | Agency for Healthcare Research and Quality | USA | Community | Asthma | Structured telephone support | Care according to guideline recommendations. |
| Gattis 1999 ^46^ | Not reported | USA | Academic hospital | Heart failure | Structured telephone support | The pharmacist explained the purpose of each drug and the importance of adherence. |
| Gellis 2014 ^47^ | New York State Department of Health | USA | Community | Heart failure or COPD | Device-based monitoring | n.a. |
| GESICA 2005 ^48^ | GESICA Foundation; Roche; Boehringer Ingelheim; BagÃ³; Pharmacia; Novartis; Merck Sharp; Dohme | Argentina | Mixed | Heart failure | Structured telephone support | Three-monthly in-clinic follow-up. |
| Giordano 2009 ^49^ | Italian Ministry of Health | Italy | Hospital | Heart failure | Structured telephone support | Pre-discharge education. |
| Goodwin 2014 ^50^ | Novartis Pharmaceuticals | Canada; USA | Hospital | Breast cancer | Structured telephone support | n.a. |
| Gray 2000 ^51^ | National Library of Medicine's Telemedicine Initiative | USA | Hospital, NICU | Low birth weight | Web-based monitoring | n.a. |
| Hale 2016 ^52^ | Presentcare Inc | USA | Hospital | Heart failure | Device-based monitoring | n.a. |
| Halimi 2008 ^53^ | Biotronik Inc | France | Hospital | Heart failure | Device-based monitoring | n.a. |
| Hannan 2013 ^54^ | Not reported | USA | Unclear | n.a. | Structured telephone support | n.a. |
| Hansen 2018 ^55^ | Abbott (formerly St Jude Medical) | Germany | Hospital | Heart failure | Structured telephone support | n.a. |
| Hanssen 2009 ^56^ | Haukeland University Hospital; the Norwegian Nurse Association; the Meltzer Foundation for grants; the Norwegian Lung and Heart Foundation. | Norway | Academic hospital | Myocardial infarction | Structured telephone support | n.a. |
| Harrison 2011 ^57^ | Surgical Outcomes Research Centre | Australia | Hospital | Colorectal cancer | Structured telephone support | n.a. |
| Härter 2016a ^58^ | Kaufmännische Krankenkasse Hannover | Germany | Unclear | Various chronic conditions; heart failure; depression or schizophrenia | Structured telephone support | Not reported |
| Härter 2016b ^58^ | Kaufmännische Krankenkasse Hannover | Germany | Unclear | Heart failure | Structured telephone support | Not reported |
| Härter 2016c ^58^ | Kaufmännische Krankenkasse Hannover | Germany | Unclear | Depression or schizophrenia | Structured telephone support | Not reported |
| Hebert 2008 ^59^ ^60^ | AHRQ | USA | Hospital | Heart failure | Structured telephone support | n.a. |
| Hindricks 2014 ^61^ | Biotronik SE & Co | Germany | Hospital | Heart failure | Device-based monitoring | n.a. |
| Ho 2016 ^62^ | National Taiwan University (NT-CESRP-101R7608-3) | Taiwan | University hospital | COPD | Web-based monitoring | n.a. |
| Imhof 2012 ^63^ | Age Foundation Zurich, Ebnet Foundation Teufen, Heinrich und Erna Walder Foundation Zurich, City of Winterthur | Switzerland | Community | n.a. | Structured telephone support | n.a. |
| Ishani 2016 ^64^ | VA Center for Innovation | USA | Hospital | Chronic kidney disease | Device-based monitoring | n.a. |
| Jakobsen 2015 ^65^ | The Philanthropic Foundation TrygFonden (grant 7561-08), The Health Insurance Foundation (grant 2011B003), The Danish Lung Association, The Toyota Foundation (grant OH/BG 7003), The Frederiksberg Foundation (grant 2010-88), and a Lykfeldtâ€™s grant. | Denmark | University hospital | COPD | Videoconferencing | n.a. |
| Javadpour 2013 ^66^ | Shiraz University of Medical Science | Iran | Hospital | Bipolar disorder | Structured telephone support | Pharmacotherapy (and eight psychoeducation sessions) |
| Jerant 2001 ^67^ | UCD School of Medicine Hibbard E. Williams research grant | USA | Unclear | Heart failure | Structured telephone support | Two in-person visits, and provision of emergency contact numbers. |
| Jódar-Sánchez 2014 ^68^ | The Spanish Ministry of Science and Innovation. | Spain | Community | COPD | Device-based monitoring | n.a. |
| Kalter-Leibovici 2017 ^69^ | Maccabi Institute for Health Services Research; The Medical Research Infrastructure Development and Health Services Fund by the Sheba Medical Center | Israel | Hospital | Heart failure | Device-based monitoring | Bi-annual in-clinic follow-up visits |
| Kessler 2018 ^70^ | Air Liquide Healthcare | Multinational | Community | COPD | Mobile telemonitoring | n.a. |
| Ko 2017 ^71^ | Chinese University of Hong Kong | Hong Kong | Hospital | COPD | Structured telephone support | Two in-person 1-hour educational sessions |
| Koehler 2011 ^72^ | Greman Federal Ministry of Economics and Technology; Robert Bosch Healthcare; InterComponentWare; Aipermon | Germany | Hospital | Heart failure | Device-based monitoring | Care according to guidelines |
| Kraai 2016 ^73^ | Dutch Ministry of Health, Department of Pharmaceutical Affairs and Medical Technology. | The Netherlands. | Hospital | Heart failure | Device-based monitoring | Computer Decision Support System providing guideline-based treatment recommendations. |
| Krum 2013 ^74^ | National Health and Medical Research Council; National Heart Foundation of Australia; Medical Benefits Fund | Australia | Community | Heart failure | Interactive voice response | Care according to guidelines, and an individualized patient diary. |
| Kulshreshtha 2010 ^75^ | Partners Healthcare | USA | Hospital | Heart failure | Device-based monitoring | n.a. |
| Laramee 2003 ^76^ | University of Vermont General Clinical Research Center; Novartis Pharmaceuticals | Canada | Hospital | Heart failure | Structured telephone support | Standard in-patient care plus case manager, 15-page CHF booklet, weight logs, self-care activities summary sheets, computerized medication lists, a guide for measuring sodium intake, as well as scales and pillboxes as needed. |
| Lavesen 2016 ^77^ | Capital Region of Denmark | Denmark | Hospital | COPD | Structured telephone support | Appointment in the outpatient clinic 3 months post discharge. A discharge summary was sent to the GP. |
| Lindegaard Pedersen 2017 ^78^ | Aarhus University Hospital | Denmark | Hospital | Malnourishment | Structured telephone support | Standard in-hospital care. Discharge arrangements with home care provider, including meal service, food delivery, and home care. |
| Luthje 2015 ^79^ | Medtronic Inc. | Germany | Hospital | Heart failure | Device-based monitoring | n.a. |
| Lyng 2012 ^80^ | The Swedish Governmental Agency for Innovation Systems; the Swedish Heart and Lung foundation | Sweden | Hospital | Heart failure | Device-based monitoring | n.a. |
| Mabo 2012 ^81^ | Biotronik SE and Co. KG | France | Hospital | Heart failure | Device-based monitoring | No in-clinic follow-ups unless indicated by a level 1 or 2 alarm. |
| Martin-Lesende 2013 ^82^ | Spanish Ministry of Health, Social Services and Equality | Spain | Hospital | Heart failure and / or chronic lung disease | Mobile telemonitoring | Regular medical examinations and on-demand telephone contacts or home visits. |
| Mayo 2008 ^83^ | Canadian Institute of Health Research | Canada | Hospital | Stroke | Structured telephone support | n.a. |
| Milsis 2012 ^84^ | EU / e-TEN project \Healthwear\"" | Greece | Hospital | COPD | Device-based monitoring | n.a. |
| Morgan 2017 ^85^ | British Heart Foundation; Boston Scientific Ltd; Medtronic Ltd; St Jude Medical | UK | Hospital | Heart failure | Device-based monitoring | Alerts for device malfunction. |
| Olivari 2018 ^86^ | European Commission | Italy | Hospital | Heart failure | Device-based monitoring | n.a. |
| Ong 2016 ^87^ | AHRQ | USA | Academic hospital | Heart failure | Device-based monitoring | n.a. |
| Osmera 2014 ^88^ | Faculty of Health and Social Studies, University of South Bohemia | Czech Republic | Hospital | Heart failure | Device-based monitoring | Yearly outpatient visits. |
| Paquette 2013 ^89^ | Quebec Interuniversity Nursing Intervention Research Group; Quebec MinistÃ¨re de l'Ã‰ducation, du Loisir et du Sport; University of Montreal; The Gustav Levinschi Foundation of the CHU Sainte-Justine; The Canadian Nurses Foundation; The Faculty of Nursing, University of Montreal | Canada | Academic Hospital | Tonsillitis | Structured telephone support | n.a. |
| Pekmezaris 2012 ^90^ | New York State Department of Health | USA | Community | Heart failure | Videoconferencing | Face-to-face nurse visits at the nurse's discretion. |
| Pekmezaris 2018 ^91^ | Patient-Centered Outcomes Research Institute | USA | Hospital | Heart failure | Device-based monitoring | Routine visits every three months |
| Phillips 2001 ^92^ | Not reported | USA | Hospital | Spinal cord injury | Videoconferencing | Scheduled post-discharge visit at 2 months. |
| Pinnock 2013 ^93^ | Chief Scientist Office, NHS Applied Research Programme Grant | Scotland | Hospital | COPD | Device-based monitoring | Self-management booklet, written management plan, emergency medication supply. |
| Riegel 2002 ^94^ | Pfizer Inc. | USA | Unclear | Heart failure | Structured telephone support | n.a. |
| Riegel 2006 ^95^ | American Heart Association | USA | Hospital | Heart failure | Structured telephone support | Written discharge instructions. Verbal if Spanish speaking personnel was available. |
| Ringbaek 2015 ^96^ | Not reported | Denmark | Hospital | COPD | Device-based monitoring | All patients were managed according to national and international guidelines. |
| Rollman 2009 ^97^ | NIH | USA | Hospital | Depression | Structured telephone support | At the discretion of patients' PCP. |
| Sardu 2016 ^98^ | NIH | Italy | Hospital | Heart failure | Device-based monitoring | Follow-up with the treating physician at 10 days after hospital discharge, and at 1, 3, 6, and 12 months. |
| Scherr 2009 ^99^ | Novartis Pharma Austria; Roche Pharma Austria; Mobilkom Austria | Austria | Hospital | Heart failure | Device-based monitoring | Pharmacological intervention. |
| Schwarz 2008 ^100^ | National Institute of Nursing Research; NIH; Ohio Board of Regents | USA | Hospital | Heart failure | Device-based monitoring | n.a. |
| Seto 2012 ^101^ | Toronto General Hospital Foundation; Natural Sciences and Engineering Research Council of Canada Strategic Research Network | Canada | Hospital | Heart failure | Device-based monitoring | Clinic visits every 2 weeks to every 3 to 6 months depending on disease severity. |
| Shany 2017 ^102^ | The Department of State and Regional Development of New South Wales Government; the Australian Research Council; the Sydney West Area Health Service; University of New South Wales. | Australia | Community | COPD | Device-based monitoring | Weekly scheduled home visits by a respiratory nurse. |
| Smolis-Bąk 2015 ^103^ | Not reported | Poland | Hospital | Heart failure | Device-based monitoring | Patients trained in the rehabilitation unit for an average of 3 weeks. |
| Soran 2008 ^104^ | Centers for Medicare & Medicaid Services Baltimore | USA | Hospital | Heart failure | Device-based monitoring | One-on-one educational session and heart failure booklet. |
| Soriano 2018 ^105^ | FundaciÃ³n TeÃ³filo Hernando, Universidad AutÃ³noma de Madrid; Linde Healthcare. | Spain | Hospital | COPD | Device-based monitoring | n.a. |
| Sorknaes 2013 ^106^ | European Commission; Danish Health Foundation; Danish Nurses' Organization; University of Southern Denmark; OUH-Odense University Hospital; Svendborg Hospital. | Denmark | Hospital, multicentre | COPD | Videoconferencing | NA |
| Spaniel 2015 ^107^ | Ministry of Health | Czech Republic | NA | Schizophrenia or schizoaffective disorder | Device-based monitoring | n.a. |
| Steventon 2012 ^108^ | Department of Health | England | Community | COPD, heart failure, or diabetes | Device-based monitoring | n.a. |
| Takahashi 2012 ^109^ | Mayo Foundation Institutional Funds; National Center for Research Resources, NIH; NIH Roadmap for Medical Research | USA | Hospital | Miscellaneous | Device-based monitoring | Access to primary and specialty office visits, phone nursing, urgent clinic visits, and ER visits. |
| Tomita 2009 ^110^ | National Institute on Aging | USA | Unclear | Heart failure | Web-based monitoring | Three-month regular check up. |
| Tsuchihashi-Makaya 2013 ^111^ | Japanese Ministry of Health, Labour and Welfare; the Japan Heart Foundation; Pfizer Health Research Foundation | Japan | Hospital | Heart failure | Structured telephone support | Medical treatment, routine cardiologist follow-up, and biweekly home visits until 2 months post-discharge. |
| Van Den Berg 2016 ^112^ | Hospital Trust Funds; NHMRC Partnership Grant Cognitive Impairment and Physical Conditions | Australia | Hospital | Stroke | Videoconferencing | n.a. |
| Vasilopoulou 2017 ^113^ | General Secretariat for Research and Technology; National Strategic Reference Framework, European Union. | Greece | Hospital | COPD | Device-based monitoring | n.a. |
| Venter 2012 ^114^ | Lakes District Health Board, Lake Taupo Primary Health Organisation; Healthcare of New Zealand | New Zealand | Primary care | Mixed | Device-based monitoring | Regular home visits, and systematic assessment and care planning. |
| Vesterby 2017 ^115^ | CareTech Innovation, European Regional Development Fund; Fund for Clinical Research, Central Denmark Region; Animation Hub, Danish Ministry of Science, Innovation and Higher Education | Denmark | Hospital | Hip replacement | Videoconferencing | n.a. |
| Vianello 2016 ^116^ | European Commission | Italy | Unclear | COPD | Device-based monitoring | Medical treatment according to guidelines. No other structural care. |
| Vuorinen 2014 ^117^ | The Finnish Funding Agency for Technology and Innovation; VTT Technical Research Centre of Finland | Finland | Hospital | Heart failure | Device-based monitoring | Support for self-management by a team of 2 physicians, a specialized heart failure nurse, and a physiotherapist. |
| Wade 2011 ^118^ | Aetna Inc; Intel Inc | USA | Community | Heart failure | Device-based monitoring | Case management facilitating healthcare processes. |
| Wagenaar 2019 ^119^ | Foundation 'Care Within Reach' | The Netherlands | Hospital | Heart failure | Device-based monitoring | n.a. |
| Wakefield 2008 ^120^ | Department of Veterans Affairs, Veterans Health Administration, Health Services Researech and Development | USA | Hospital | Heart failure | Videoconferencing | In-clinic follow-ups. |
| Waldmann 2008 ^121^ | AOK Schleswig-Holstein; Card Guard Europe; Segeberger Kliniken | Germany | Hospital | Coronary artery disease | Device-based monitoring | n.a. |
| Walker 2018 ^122^ | European Commission | UK, Estonia, Sweden, Spain, Slovenia | Hospital | COPD | Device-based monitoring | n.a. |
| Weintraub 2010 ^123^ | GlaxoSmithKline Inc; Philips Medical Systems Inc; Health Hero Network Inc | USA | Hospital | Heart failure | Device-based monitoring | n.a. |
| Wong 2005 ^124^ | Not reported | China | Hospital | COPD | Structured telephone support | n.a. |
| Xu 2010 ^125^ | Asthma Foundations of Australia; Royal Children's Hospital Foundation Brisbane Australia. | Australia | Hospital | Asthma | Structured telephone support | GP or hospital outpatient care. |
| Young 2013 ^126^ | Cancer Institute New South Wales Health Services Research Program | Australia | Hospital | Colorectal cancer | Structured telephone support | n.a. |
| Zhao 2009 ^127^ | Hong Kong Polytechnic University | China | Community | Coronary heart disease | Structured telephone support | Two home visits (one in week 1, one in week 3). |
