# Supplementary material for: The Effect of Telehealth on Hospital Services Use: Systematic Review and Meta-analysis
Source: J Med Internet Res. 2021 Sep 1;23(9):e25195. doi: 10.2196/25195 (PMC8444037; doi:10.2196/25195)
Supplement: Multimedia Appendix 4 [file jmir_v23i9e25195_app4.docx]

**Multimedia Appendix 4: GRADE Protocol**

**Risk of bias**

Rate down one level if:
There are studies with a high risk of bias for any one domain that cumulatively account for a weight of 60% in an analysis. For example, if 4 studies in one analysis are all rated at high risk of bias for incomplete outcome data, and each of those studies received a weight of 15% in the meta-analysis, the quality of evidence would be rated down by one level.

OR

There are studies with an unclear risk of bias for any three domains, which cumulatively account for a weight of 60% in an analysis.

Rate down two levels if:
There are studies with a high risk of bias for any two domains that cumulatively account for a weight of 60% in an analysis. For example, if 4 studies in one analysis are all rated at high risk of bias for incomplete outcome data, and each of those studies received a weight of 15% in the meta-analysis, the quality of evidence would be rated down by one level.

OR

There are studies that have a high risk of bias for any one domain, AND an unclear risk of bias for any three domains, cumulatively accounting for a weight of 60% in an analysis.

**Inconsistency**Rate down by one level if:

Unexplained heterogeneity is at least equal to 60% for 3 of the 4 methods of stratification (by health condition, telehealth type, follow-up, and risk of bias). Unexplained heterogeneity is computed as

$$Unexplained heterogeneity= I^{2}*Residual heterogeneity$$

**Imprecision**Rate down by one level if:

Fewer than 2000 participants are included in the analysis AND the confidence interval of the point estimate overlaps no effect.

Rate down by two levels if:

There are very few events, and confidence intervals of both relative and absolute effects fail to exclude a null effect.

**Publication bias**Rate down by at most one level if:

Funnel plot asymmetry found by visual inspection suggests publication bias or there are much fewer small studies than large studies.
