# Supplementary material for: The Effect of Telehealth on Hospital Services Use: Systematic Review and Meta-analysis
Source: J Med Internet Res. 2021 Sep 1;23(9):e25195. doi: 10.2196/25195 (PMC8444037; doi:10.2196/25195)
Supplement: Multimedia Appendix 5 [file jmir_v23i9e25195_app5.docx]

**Multimedia Appendix 5: GRADE assessments including inconsistency, risk of bias, imprecision, and publication bias**

[Multimedia Appendix 5 Figure 1. Forest plot of all-cause hospitalizations for telehealth compared to usual care, stratified by telehealth type 4](#_Toc59008106)

[Multimedia Appendix 5 Figure 2. Forest plot of all-cause hospitalizations for telehealth compared to usual care, stratified by health condition 5](#_Toc59008107)

[Multimedia Appendix 5 Figure 3. Forest plot of all-cause hospitalizations for telehealth compared to usual care, stratified by length of follow-up 6](#_Toc59008108)

[Multimedia Appendix 5 Figure 4. Forest plot of all-cause hospitalizations for telehealth compared to usual care, stratified by risk of bias 7](#_Toc59008109)

[Multimedia Appendix 5 Figure 5. Risk of bias for each domain per study reporting all-cause hospitalizations 8](#_Toc59008110)

[Multimedia Appendix 5 Figure 6. Cumulative weighted risk of bias for each domain for all-cause hospitalizations 8](#_Toc59008111)

[Multimedia Appendix 5 Figure 7. Funnel plot for all-cause hospitalizations 9](#_Toc59008112)

[Multimedia Appendix 5 Figure 8. Forest plot of condition-related hospitalizations for telehealth compared to usual care, stratified by telehealth type 10](#_Toc59008113)

[Multimedia Appendix 5 Figure 9. Forest plot of condition-related hospitalizations for telehealth compared to usual care, stratified by health condition 11](#_Toc59008114)

[Multimedia Appendix 5 Figure 10. Forest plot of condition-related hospitalizations for telehealth compared to usual care, stratified by length of follow-up 12](#_Toc59008115)

[Multimedia Appendix 5 Figure 11. Forest plot of condition-related hospitalizations for telehealth compared to usual care, stratified by risk of bias 13](#_Toc59008116)

[Multimedia Appendix 5 Figure 12. Risk of bias per domain per study reporting condition-related hospitalizations 14](#_Toc59008117)

[Multimedia Appendix 5 Figure 13. Weighted risk of bias summary per domain for condition-related hospitalizations 14](#_Toc59008118)

[Multimedia Appendix 5 Figure 14. Funnel plot for condition-related hospitalizations 15](#_Toc59008119)

[Multimedia Appendix 5 Figure 15. Forest plot of participants with an all-cause hospitalization for telehealth compared to usual care, stratified by telehealth type 16](#_Toc59008120)

[Multimedia Appendix 5 Figure 16. Forest plot of participants with an all-cause hospitalization for telehealth compared to usual care, stratified by health condition 17](#_Toc59008121)

[Multimedia Appendix 5 Figure 17. Forest plot of participants with an all-cause hospitalization for telehealth compared to usual care, stratified by length of follow-up 18](#_Toc59008122)

[Multimedia Appendix 5 Figure 18. Forest plot of participants with an all-cause hospitalization for telehealth compared to usual care, stratified by risk of bias 19](#_Toc59008123)

[Multimedia Appendix 5 Figure 19. Risk of bias per domain per study reporting participants with an all-cause hospitalization 20](#_Toc59008124)

[Multimedia Appendix 5 Figure 20. Cumulative weighted risk of bias for each domain for participants with an all-cause hospitalization 21](#_Toc59008125)

[Multimedia Appendix 5 Figure 21. Funnel plot for participants with an all-cause hospitalization 21](#_Toc59008126)

[Multimedia Appendix 5 Figure 22. Forest plot of participants with a condition-related hospitalization for telehealth compared to usual care, stratified by telehealth type 23](#_Toc59008127)

[Multimedia Appendix 5 Figure 23. Forest plot of participants with a condition-related hospitalization for telehealth compared to usual care, stratified by health condition 24](#_Toc59008128)

[Multimedia Appendix 5 Figure 24. Forest plot of participants with a condition-related hospitalization for telehealth compared to usual care, stratified by length of follow-up 25](#_Toc59008129)

[Multimedia Appendix 5 Figure 25. Forest plot of participants with a condition-related hospitalization for telehealth compared to usual care, stratified by risk of bias 26](#_Toc59008130)

[Multimedia Appendix 5 Figure 26. Risk of bias per domain per study reporting participants with a condition-related hospitalization 28](#_Toc59008131)

[Multimedia Appendix 5 Figure 27. Weighted risk of bias summary per domain for participants with a condition-related hospitalization 29](#_Toc59008132)

[Multimedia Appendix 5 Figure 28. Funnel plot for participants with a condition-related hospitalization 29](#_Toc59008133)

[Multimedia Appendix 5 Figure 29. Forest plot for all-cause hospital days for telehealth compared to usual care, stratified by telehealth type 30](#_Toc59008134)

[Multimedia Appendix 5 Figure 30. Forest plot for all-cause hospital days for telehealth compared to usual care, stratified by health condition 31](#_Toc59008135)

[Multimedia Appendix 5 Figure 31. Forest plot for all-cause hospital days for telehealth compared to usual care, stratified by length of follow-up 32](#_Toc59008136)

[Multimedia Appendix 5 Figure 32. Forest plot for all-cause hospital days for telehealth compared to usual care, stratified by risk of bias 33](#_Toc59008137)

[Multimedia Appendix 5 Figure 33. Risk of bias per domain per study reporting all-cause hospital days 34](#_Toc59008138)

[Multimedia Appendix 5 Figure 34. Weighted risk of bias summary per domain for all-cause hospital days 34](#_Toc59008139)

[Multimedia Appendix 5 Figure 35. Funnel plot for all-cause hospital days 35](#_Toc59008140)

[Multimedia Appendix 5 Figure 36. Forest plot for condition-related hospital days for telehealth compared to usual care, stratified by telehealth type 36](#_Toc59008141)

[Multimedia Appendix 5 Figure 37. Forest plot for condition-related hospital days for telehealth compared to usual care, stratified by health condition 36](#_Toc59008142)

[Multimedia Appendix 5 Figure 38. Forest plot for condition-related hospital days for telehealth compared to usual care, stratified by length of follow-up 37](#_Toc59008143)

[Multimedia Appendix 5 Figure 39. Forest plot for condition-related hospital days for telehealth compared to usual care, stratified by risk of bias 37](#_Toc59008144)

[Multimedia Appendix 5 Figure 40. Risk of bias per domain per study reporting condition-related hospital days 38](#_Toc59008145)

[Multimedia Appendix 5 Figure 41. Weighted risk of bias summary for condition-related hospital days 38](#_Toc59008146)

[Multimedia Appendix 5 Figure 43. Forest plot for length of all-cause hospital stay for telehealth compared to usual care, stratified by telehealth type 40](#_Toc59008147)

[Multimedia Appendix 5 Figure 44. Forest plot for length of all-cause hospital stay for telehealth compared to usual care, stratified by health condition 40](#_Toc59008148)

[Multimedia Appendix 5 Figure 45. Forest plot for length of all-cause hospital stay for telehealth compared to usual care, stratified by health condition 41](#_Toc59008149)

[Multimedia Appendix 5 Figure 46. Forest plot for length of all-cause hospital stay for telehealth compared to usual care, stratified by risk of bias 41](#_Toc59008150)

[Multimedia Appendix 5 Figure 47. Risk of bias per domain per study reporting length of all-cause hospital stay 42](#_Toc59008151)

[Multimedia Appendix 5 Figure 48. Weighted risk of bias summary per domain for length of all-cause hospital stay 42](#_Toc59008152)

[Multimedia Appendix 5 Figure 49. Funnel plot for length of all-cause hospital stay 43](#_Toc59008153)

[Multimedia Appendix 5 Figure 51. Forest plot for length of condition-related hospital stay for telehealth compared to usual care, stratified by health condition 45](#_Toc59008154)

[Multimedia Appendix 5 Figure 52. Forest plot for length of condition-related hospital stay for telehealth compared to usual care, stratified by length of follow-up 46](#_Toc59008155)

[Multimedia Appendix 5 Figure 53. Forest plot for length of condition-related hospital stay for telehealth compared to usual care, stratified by risk of bias 47](#_Toc59008156)

[Multimedia Appendix 5 Figure 54. Risk of bias per domain per study reporting length of condition-related hospital stay 48](#_Toc59008157)

[Multimedia Appendix 5 Figure 55. Weighted risk of bias summary per domain for length of condition-related hospital stay 48](#_Toc59008158)

[Multimedia Appendix 5 Figure 56. Funnel plot for length of condition-related hospital stay 49](#_Toc59008159)

**All-cause hospitalizations***Inconsistency*

*
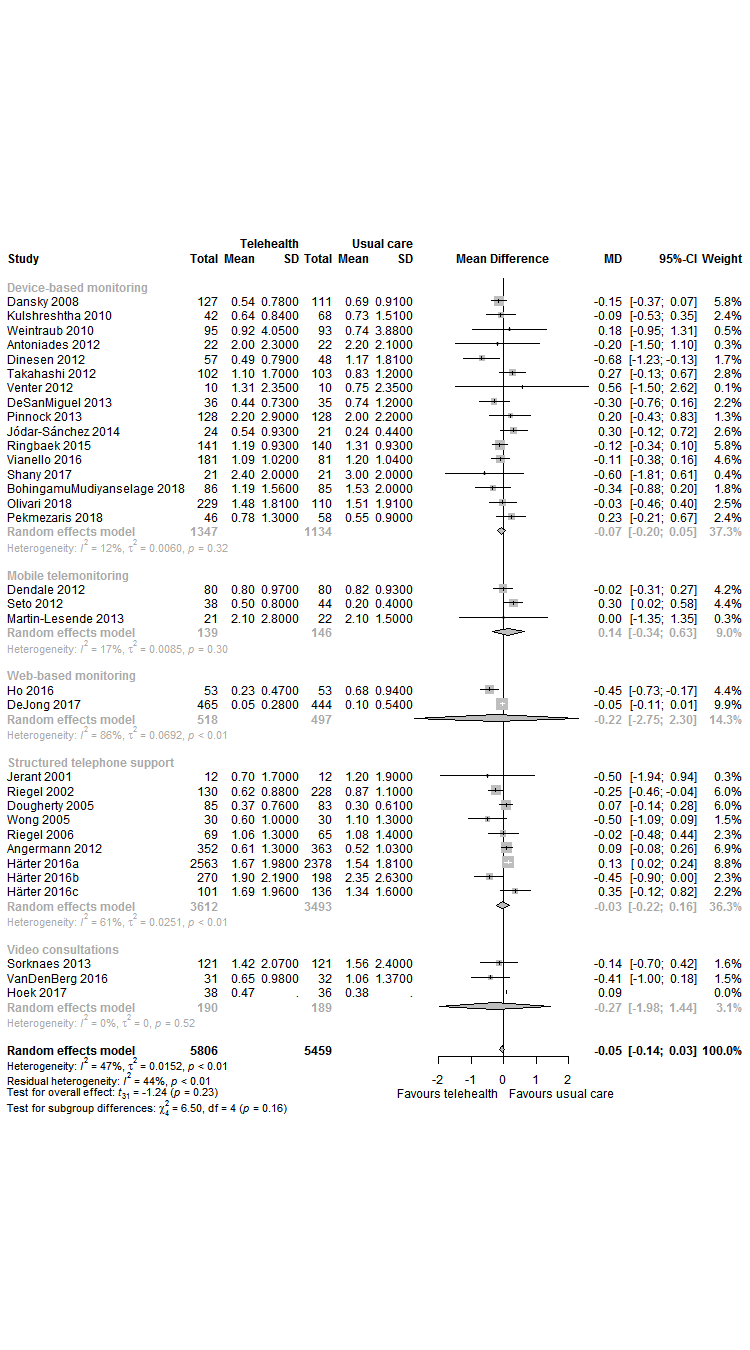
*

Multimedia Appendix 5 Figure 1. Forest plot of all-cause hospitalizations for telehealth compared to usual care, stratified by telehealth type


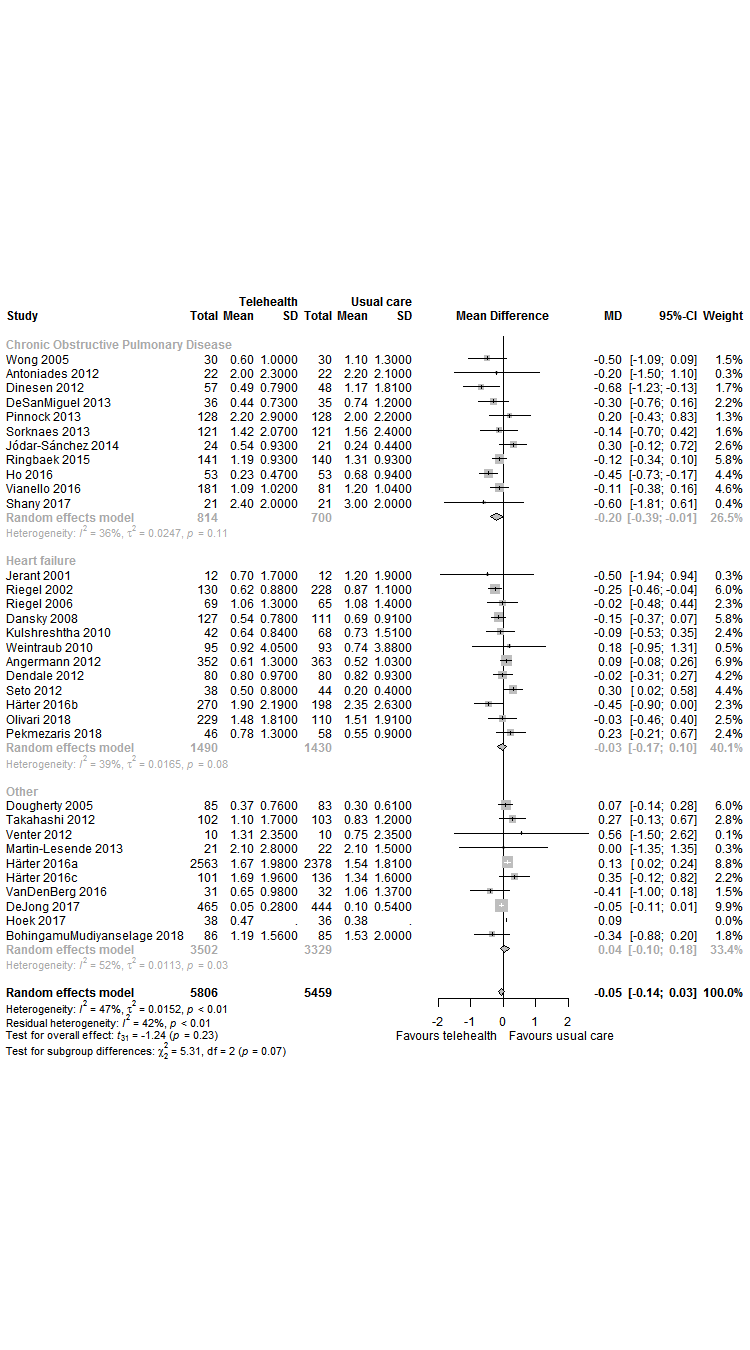


Multimedia Appendix 5 Figure 2. Forest plot of all-cause hospitalizations for telehealth compared to usual care, stratified by health condition


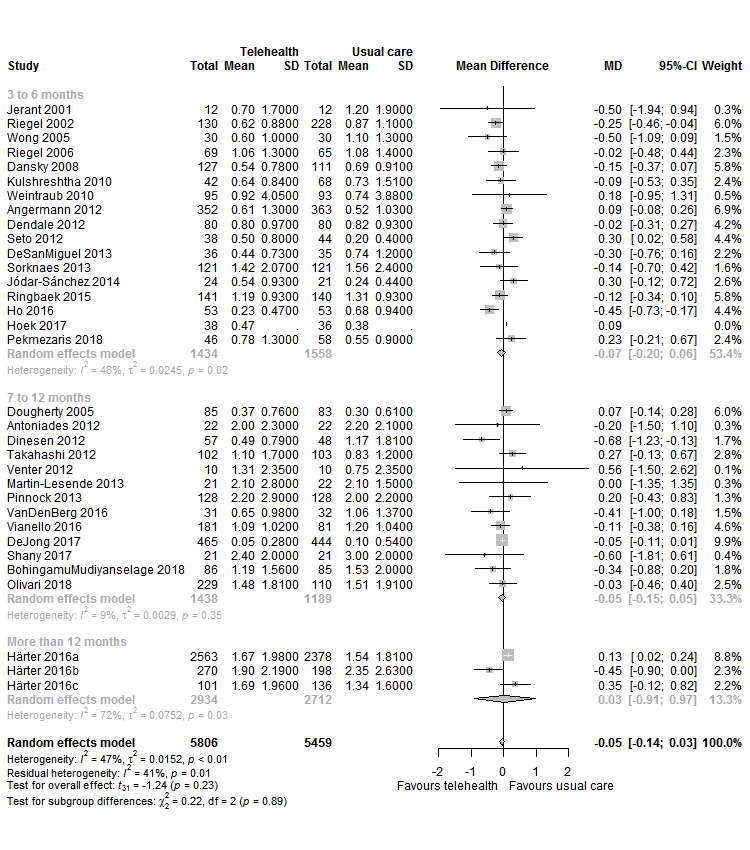


Multimedia Appendix 5 Figure 3. Forest plot of all-cause hospitalizations for telehealth compared to usual care, stratified by length of follow-up


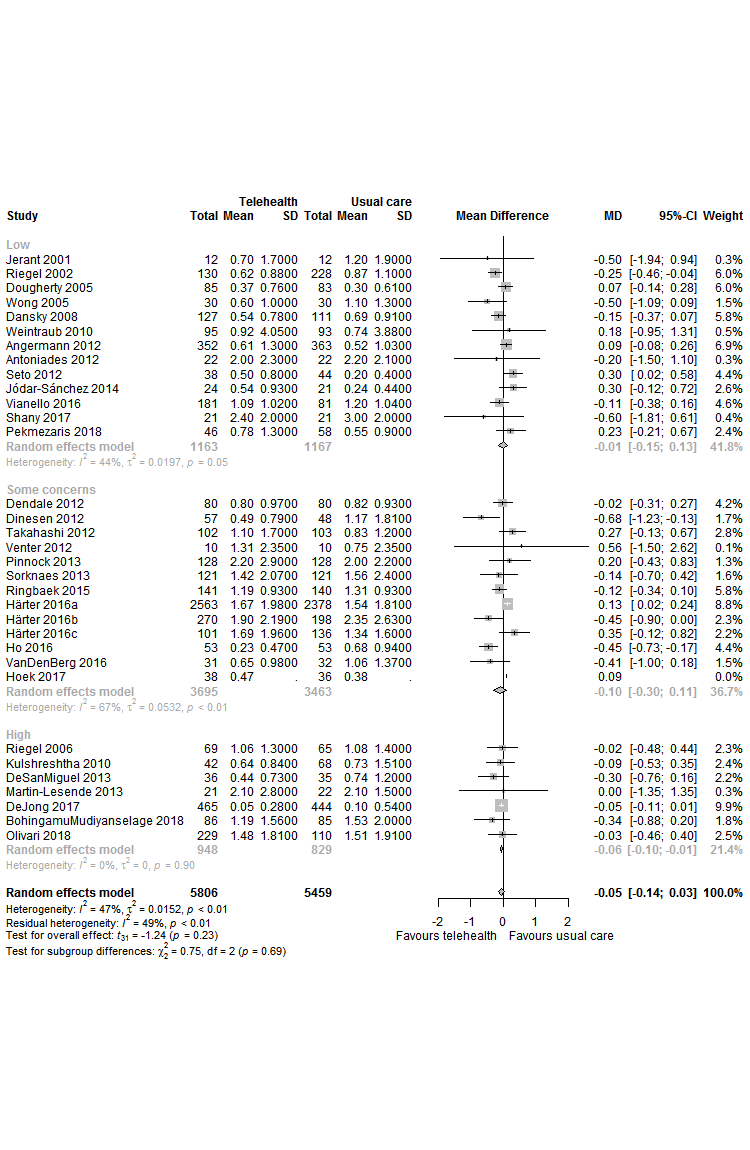


Multimedia Appendix 5 Figure 4. Forest plot of all-cause hospitalizations for telehealth compared to usual care, stratified by risk of bias

Unexplained heterogeneity is below 15% for each analysis. Additionally, the majority of confidence intervals overlaps, and although point estimates do vary, do not consider it enough to downgrade quality of evidence.

*Risk of bias*


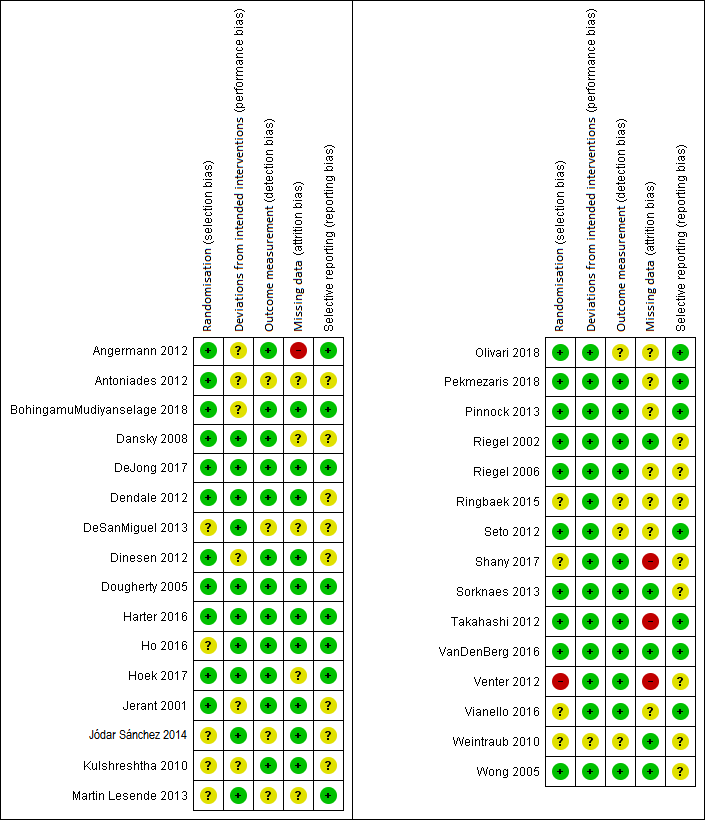


Multimedia Appendix 5 Figure 5. Risk of bias for each domain per study reporting all-cause hospitalizations


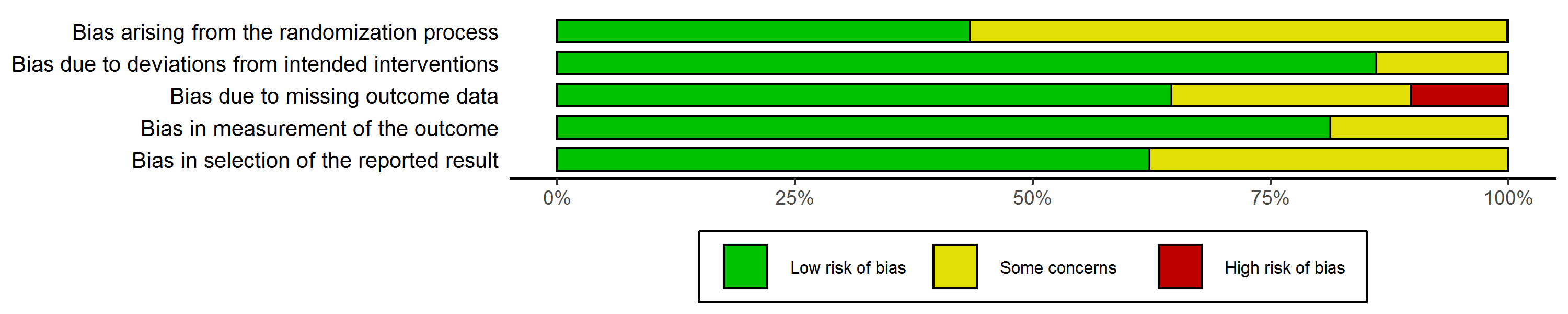


Multimedia Appendix 5 Figure 6. Cumulative weighted risk of bias for each domain for all-cause hospitalizations

The majority of studies has a low risk of bias, so the quality of evidence is not downgraded.

*Imprecision*
Although the confidence interval of the summary estimate does overlap a null effect, the analysis included well over 2000 participants. Therefore we did not downgrade quality of evidence for imprecision.

*Publication bias*


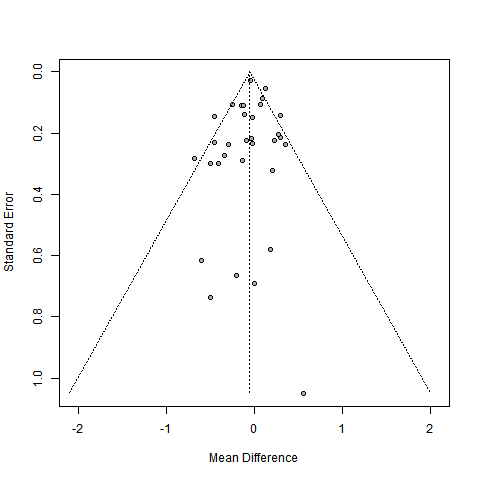


Multimedia Appendix 5 Figure 7. Funnel plot for all-cause hospitalizations

The funnel plot appears to be quite symmetrical, so downgrading for publication bias is not necessary.

Summary: Unexplained heterogeneity is well below the threshold value of 60%, the majority of studies has a low risk of bias, and risk for publication bias appears low. The confidence interval of the summary estimate overlaps a null effect (-0.14 to 0.03), however we did not downgrade the quality of evidence because of the high number of participants included in the analysis.

Overall judgement: High quality of evidence

**Condition-related hospitalizations***Inconsistency*


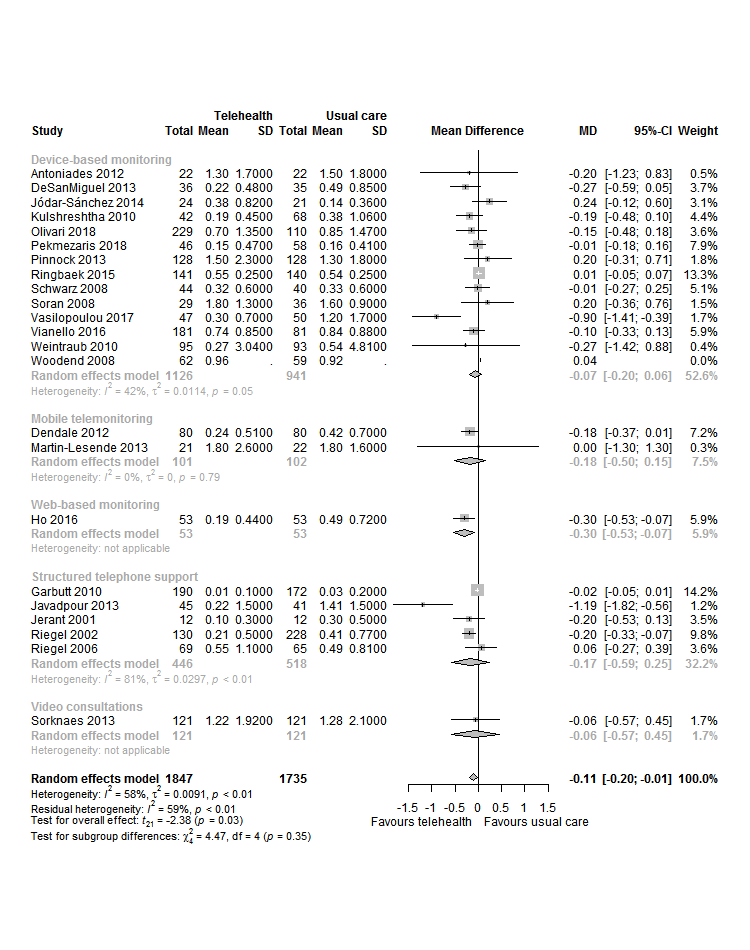


Multimedia Appendix 5 Figure 8. Forest plot of condition-related hospitalizations for telehealth compared to usual care, stratified by telehealth type


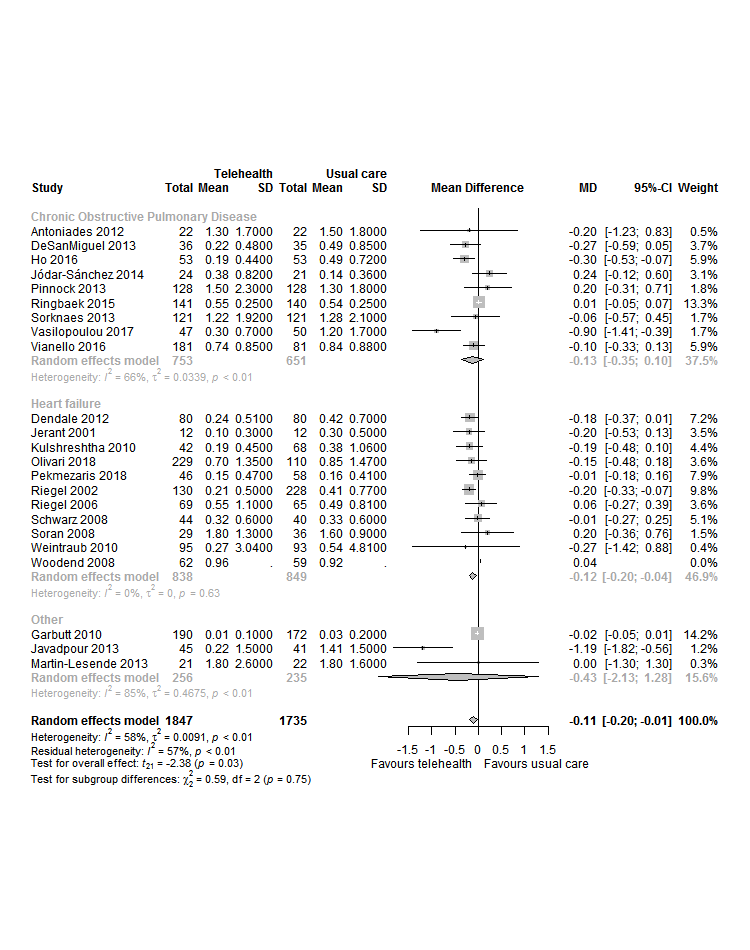


Multimedia Appendix 5 Figure 9. Forest plot of condition-related hospitalizations for telehealth compared to usual care, stratified by health condition


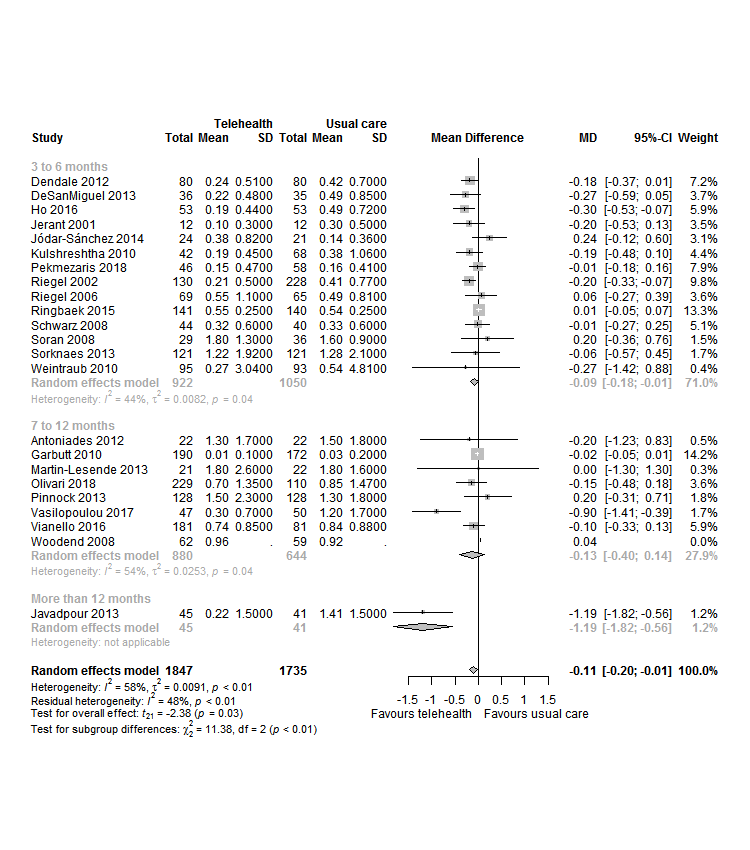


Multimedia Appendix 5 Figure 10. Forest plot of condition-related hospitalizations for telehealth compared to usual care, stratified by length of follow-up


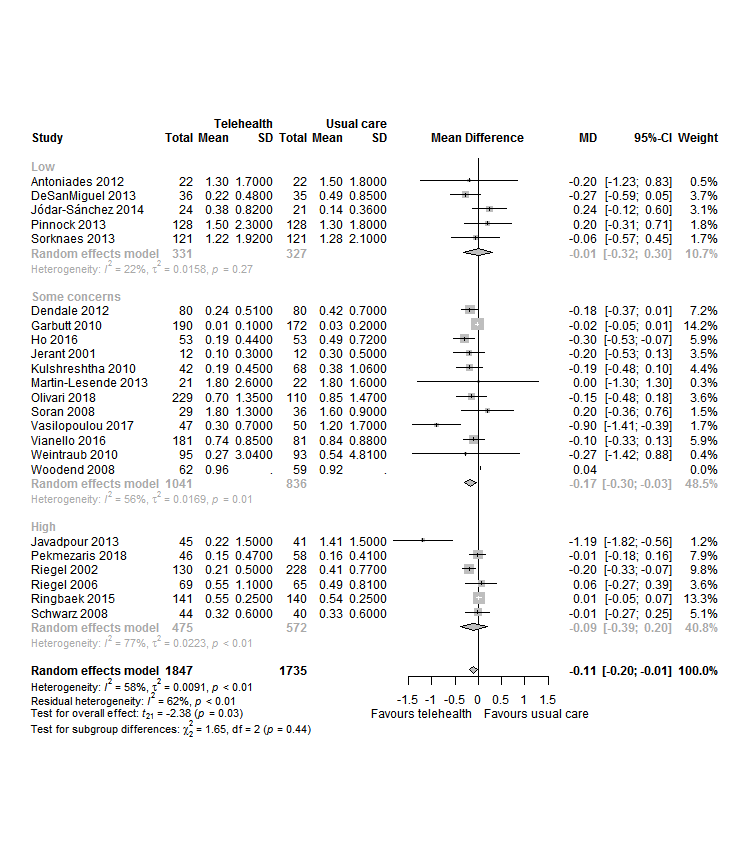


Multimedia Appendix 5 Figure 11. Forest plot of condition-related hospitalizations for telehealth compared to usual care, stratified by risk of bias

Unexplained heterogeneity is below 40% for all analyses. Additionally, the majority of confidence intervals overlap, and variation in point estimates seems reasonable. Therefore, we do not downgrade for inconsistency.

*Risk of bias*


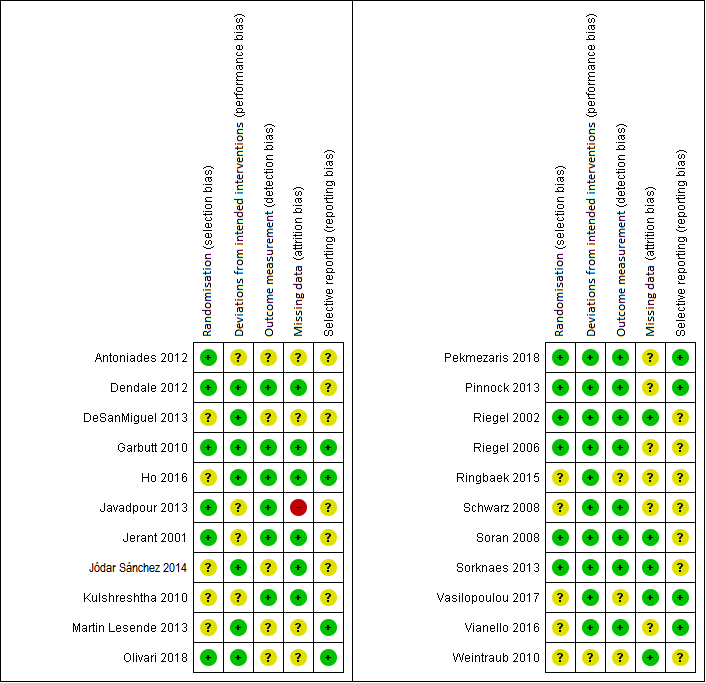


Multimedia Appendix 5 Figure 12. Risk of bias per domain per study reporting condition-related hospitalizations


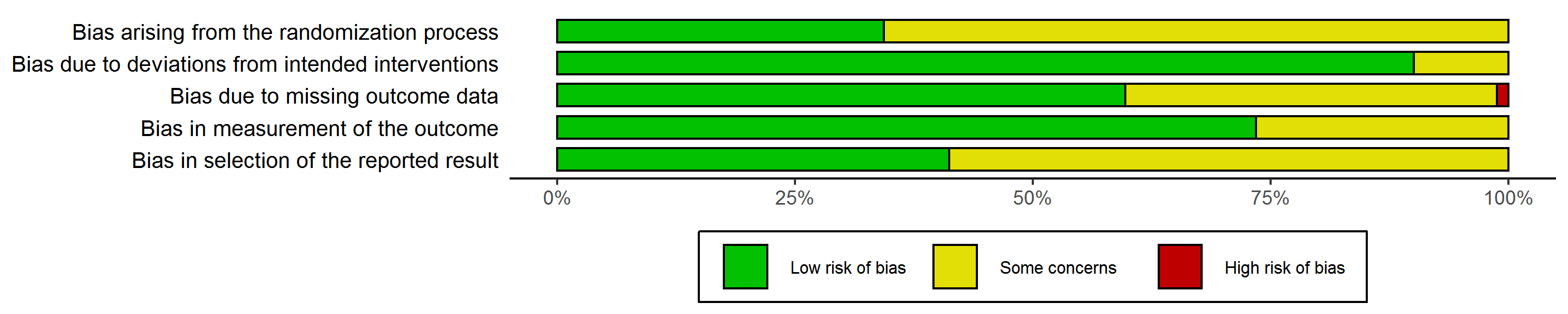


Multimedia Appendix 5 Figure 13. Weighted risk of bias summary per domain for condition-related hospitalizations

More than 50% of the weight is accounted for by studies at low risk of bias in three out of the five domains. Thus, downgrading is not necessary.

*Imprecision*
The confidence interval of the summary estimate does not overlap a null effect, and the analysis included well over 2000 participants, so there is no need to downgrade the quality of evidence.

*Publication bias*

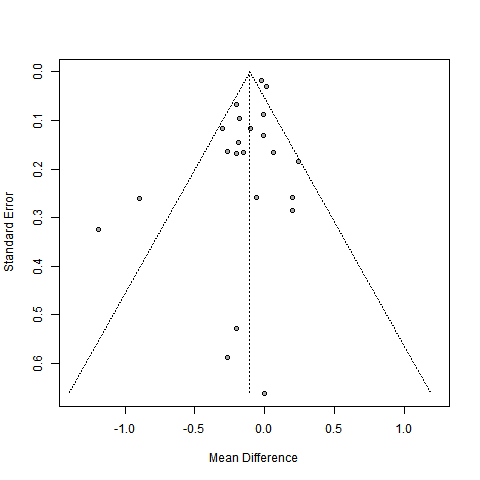


Multimedia Appendix 5 Figure 14. Funnel plot for condition-related hospitalizations

The funnel plot appears to be fairly symmetrical, so there is no reason to downgrade the quality of evidence for publication bias.

Summary: Unexplained heterogeneity is well below the threshold value of 60%, imprecision is limited owing to the large number of participants, the majority of studies has a low risk of bias, and risk for publication bias appears low.

Overall judgement: High quality of evidence.

**Participants with an all-cause hospitalization**
*Inconsistency*


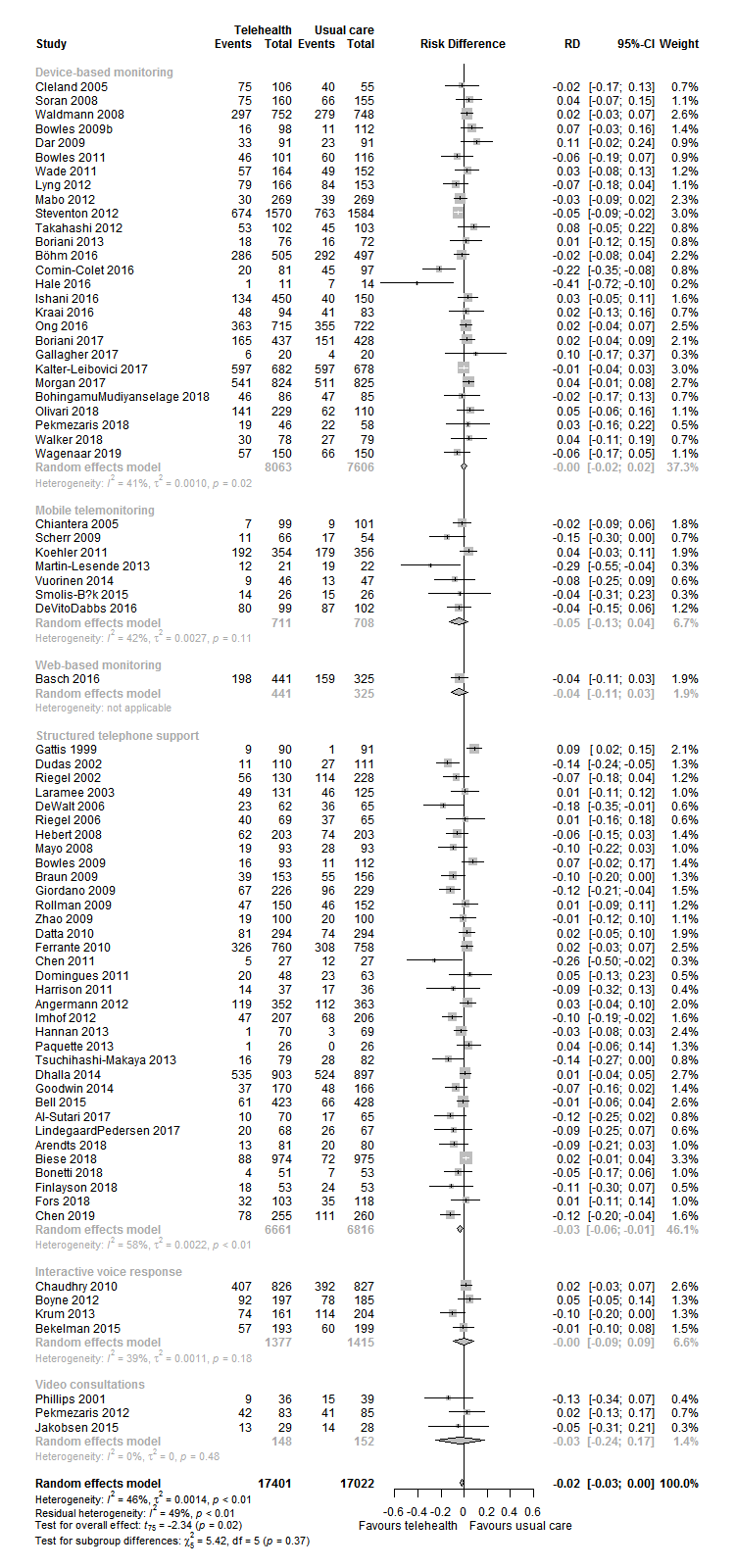


Multimedia Appendix 5 Figure 15. Forest plot of participants with an all-cause hospitalization for telehealth compared to usual care, stratified by telehealth type


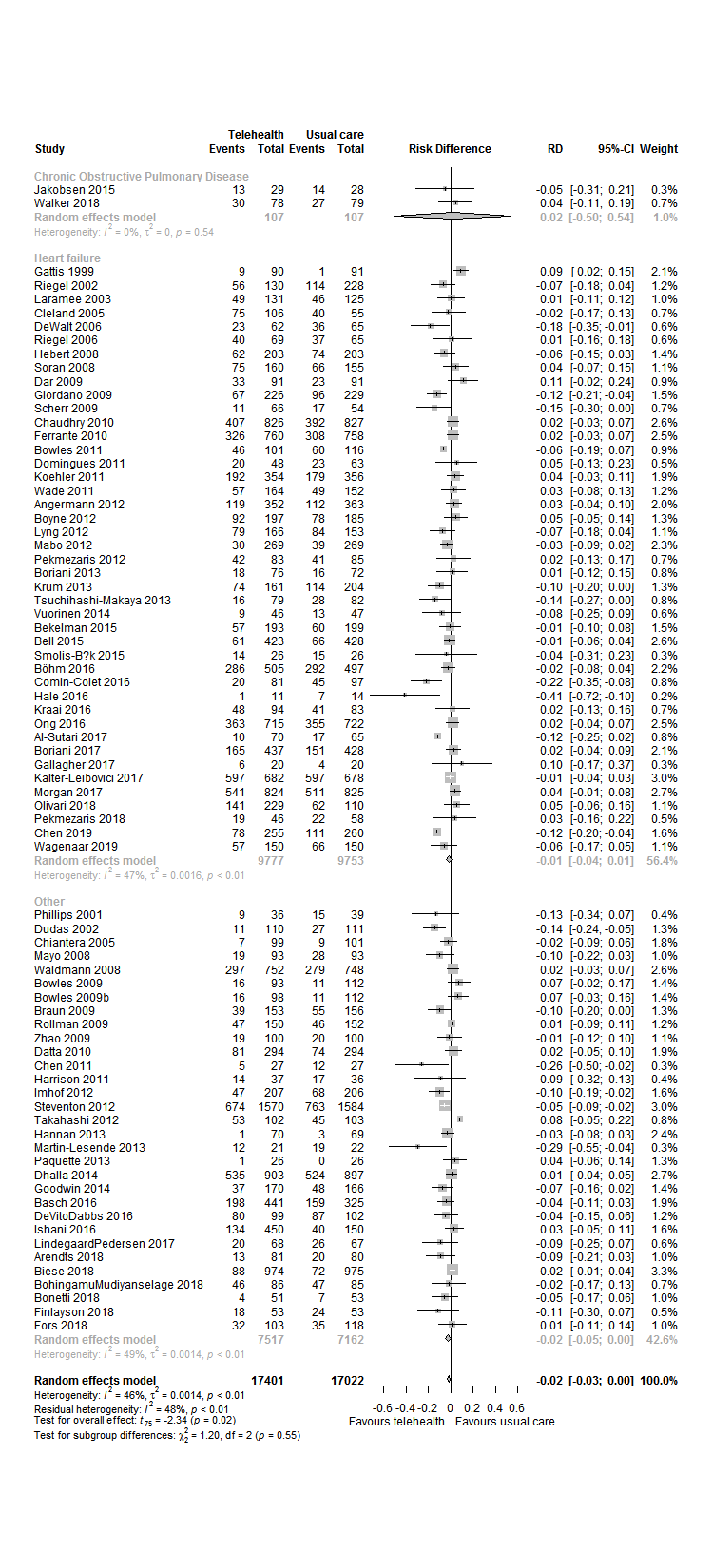


Multimedia Appendix 5 Figure 16. Forest plot of participants with an all-cause hospitalization for telehealth compared to usual care, stratified by health condition


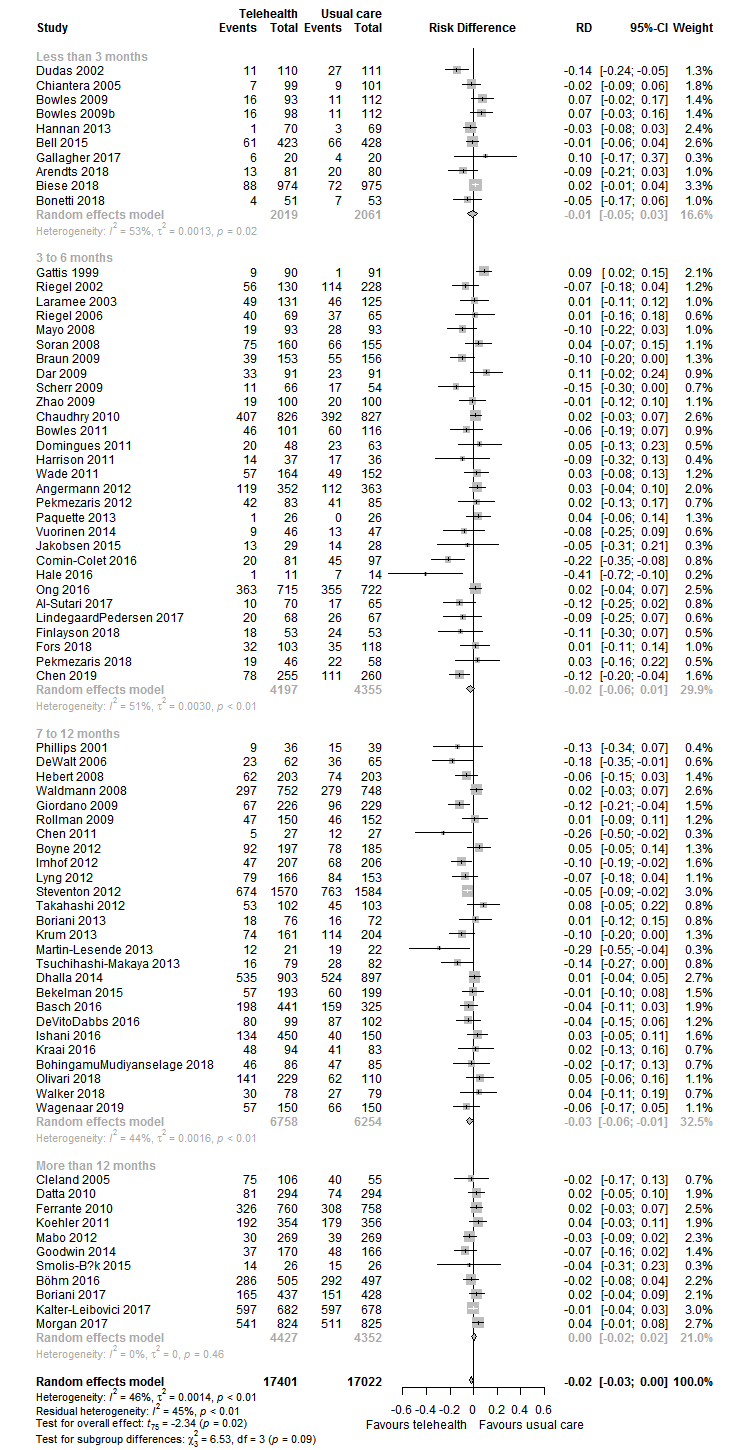


Multimedia Appendix 5 Figure 17. Forest plot of participants with an all-cause hospitalization for telehealth compared to usual care, stratified by length of follow-up


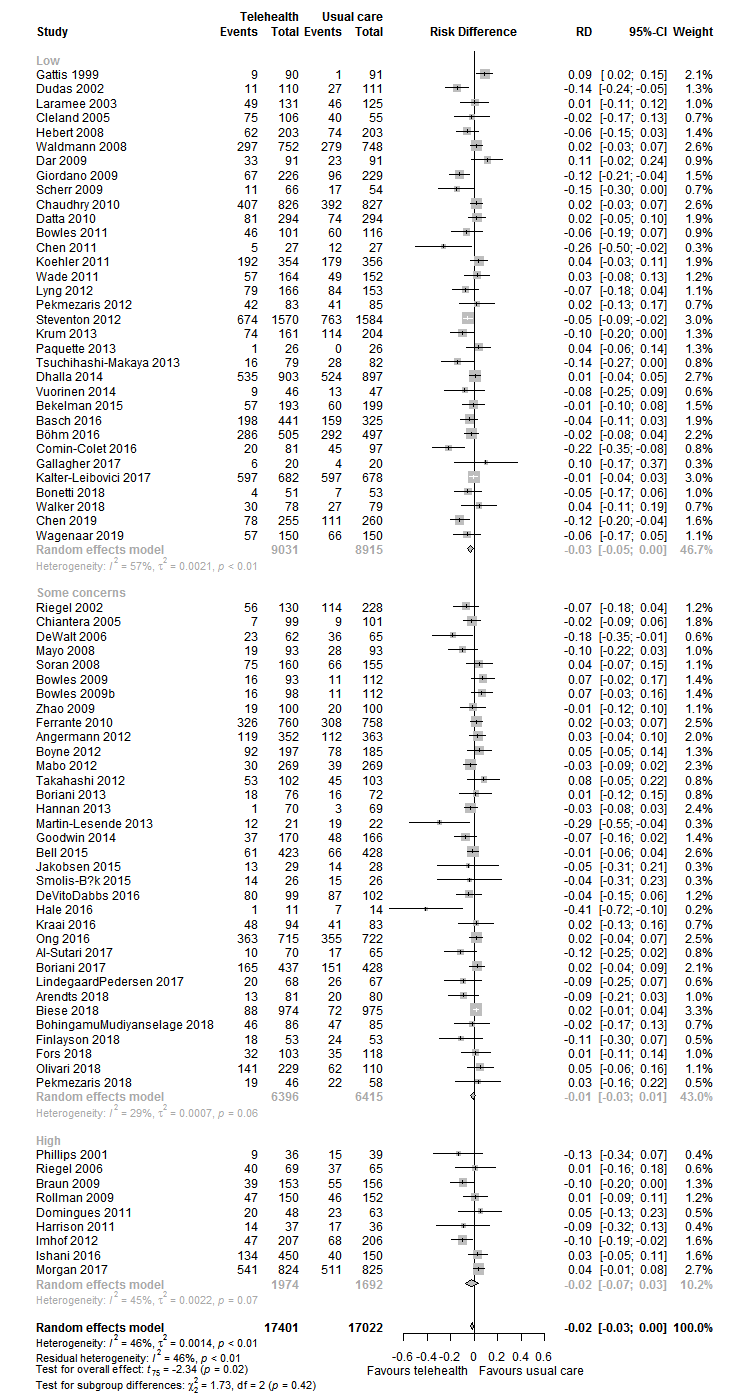


Multimedia Appendix 5 Figure 18. Forest plot of participants with an all-cause hospitalization for telehealth compared to usual care, stratified by risk of bias

The amount of unexplained heterogeneity is below 25% for each analysis. Furthermore, the majority of confidence intervals appears to overlap, and variation between point estimates seems limited. Therefore, there is no reason to downgrade quality of evidence for inconsistency.

*Risk of bias*
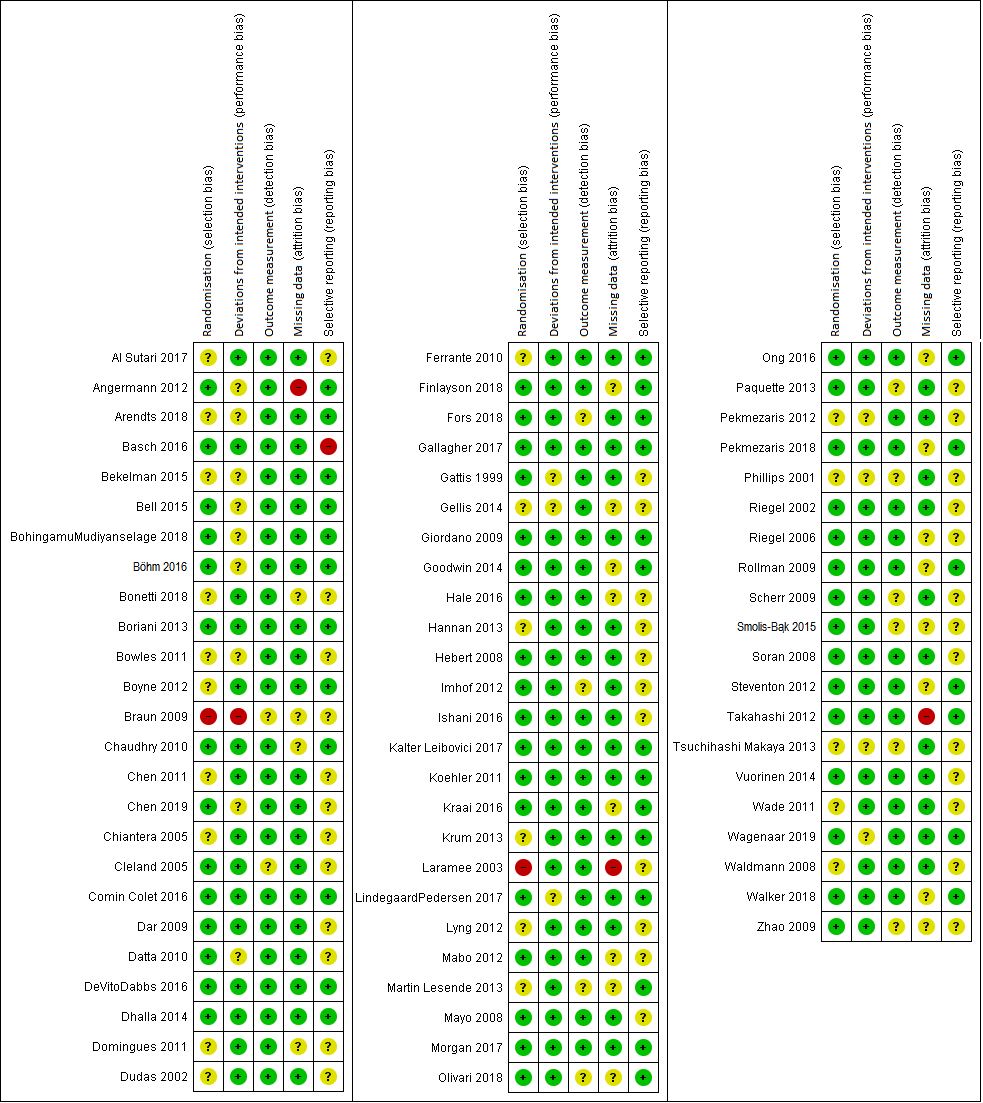


Multimedia Appendix 5 Figure 19. Risk of bias per domain per study reporting participants with an all-cause hospitalization


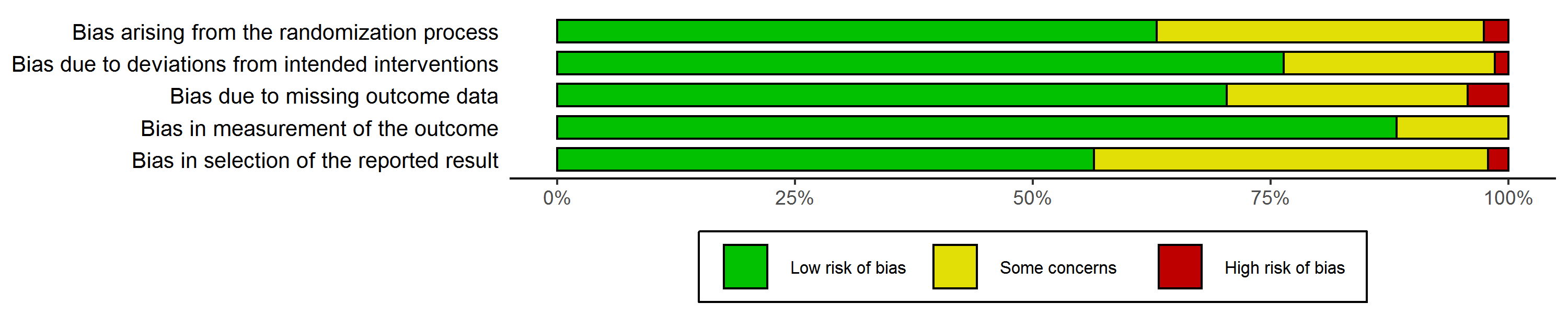


Multimedia Appendix 5 Figure 20. Cumulative weighted risk of bias for each domain for participants with an all-cause hospitalization

In each domain of risk of bias, articles with a low risk of bias represent a weight of more than 50%. Therefore, there is no reason to downgrade the quality of evidence for risk of bias.

*Imprecision*
The confidence interval of the summary estimate does not overlap a null effect, and the analysis included well over 2000 participants. Therefore, quality of evidence is not downgraded.

*Publication bias*

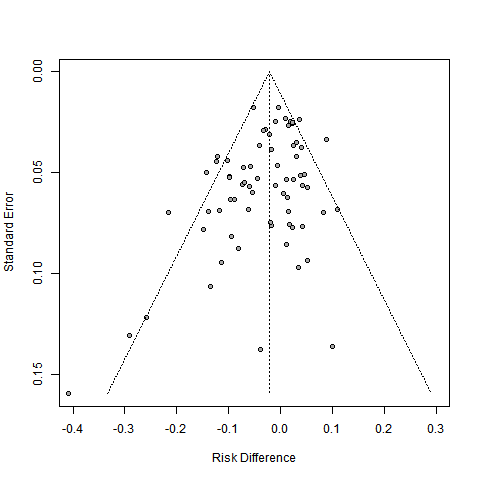


Multimedia Appendix 5 Figure 21. Funnel plot for participants with an all-cause hospitalization

The funnel plot appears to be quite symmetrical, so downgrading for publication bias does not seem necessary.

Summary: Unexplained heterogeneity is well below the threshold value of 60%, imprecision is limited owing to the large number of participants, the majority of studies has a low risk of bias, and risk for publication bias appears low.
Overall judgement: High quality of evidence

**Participants with a condition-related hospitalization**
*Inconsistency*

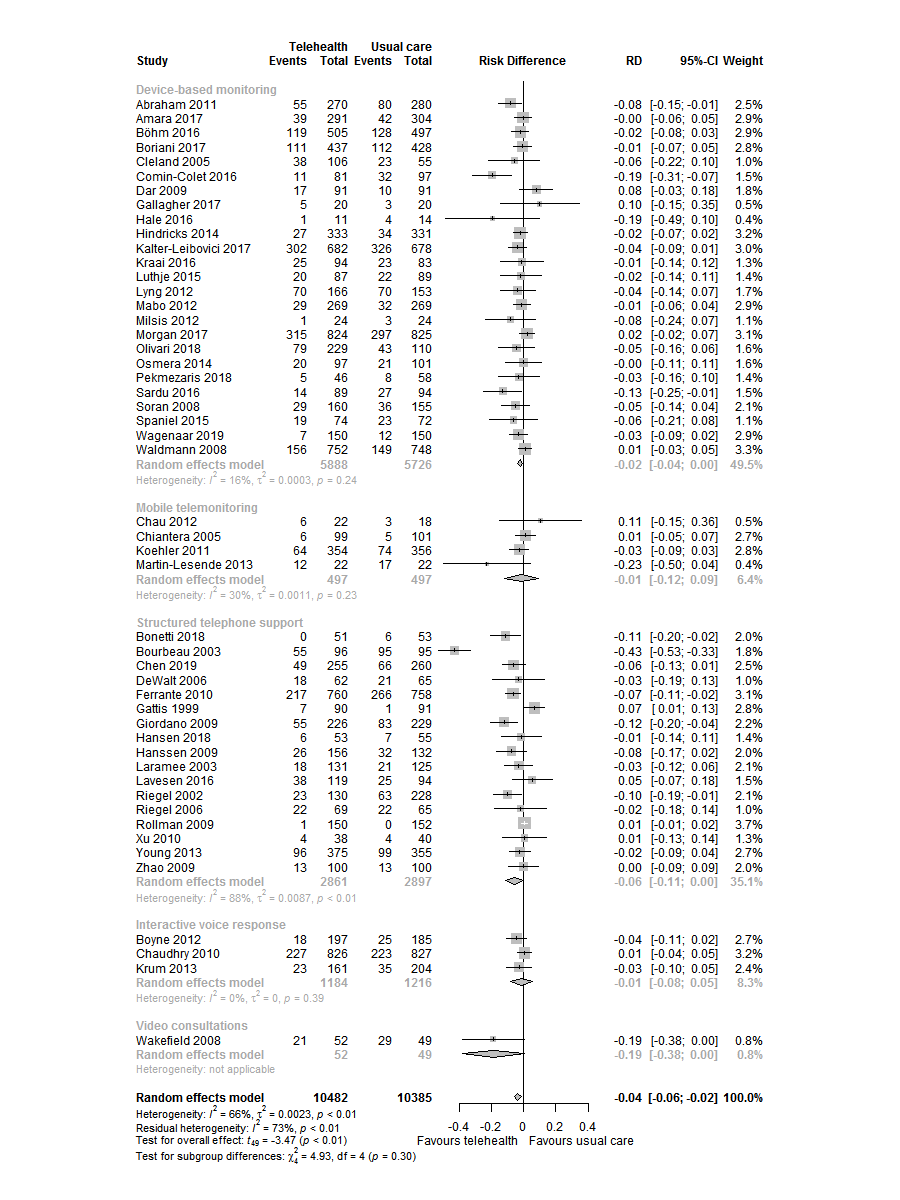


Multimedia Appendix 5 Figure 22. Forest plot of participants with a condition-related hospitalization for telehealth compared to usual care, stratified by telehealth type


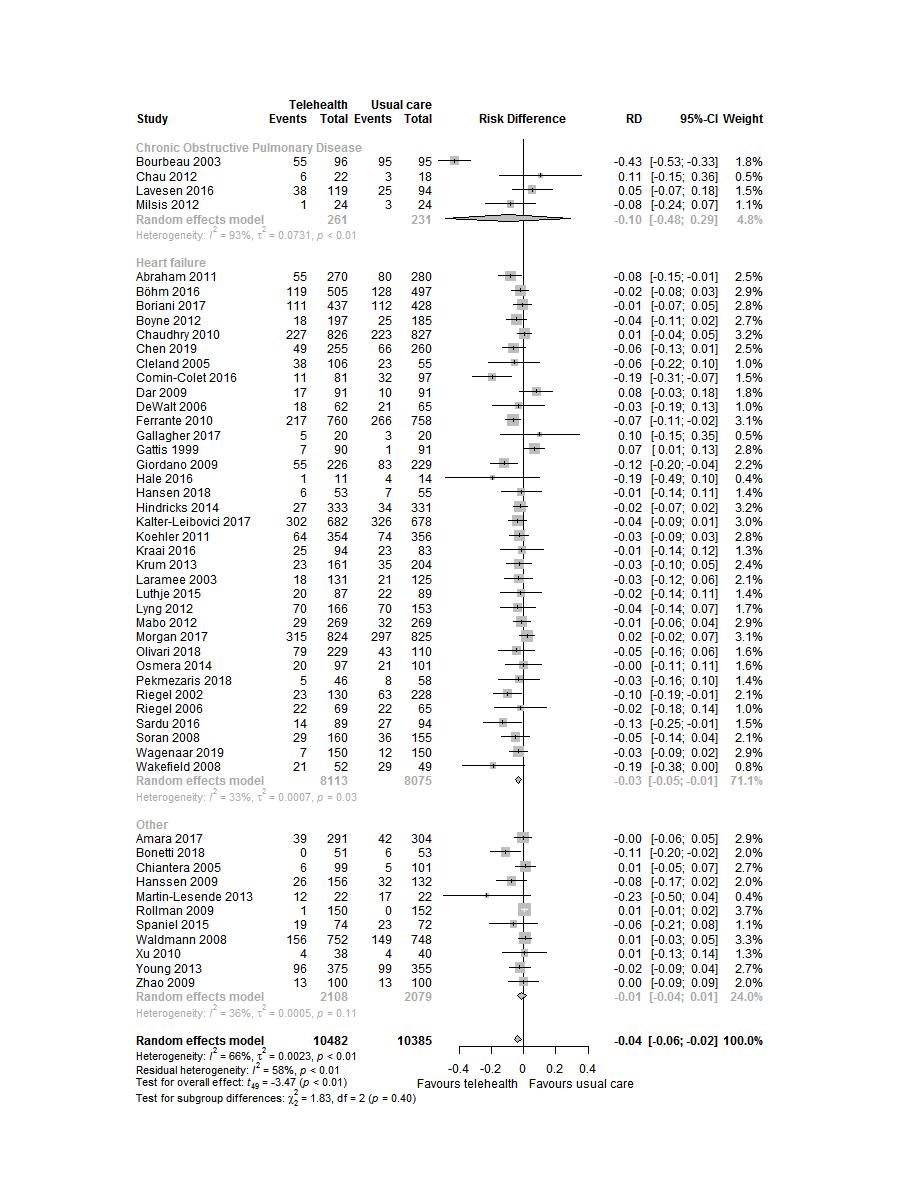


Multimedia Appendix 5 Figure 23. Forest plot of participants with a condition-related hospitalization for telehealth compared to usual care, stratified by health condition


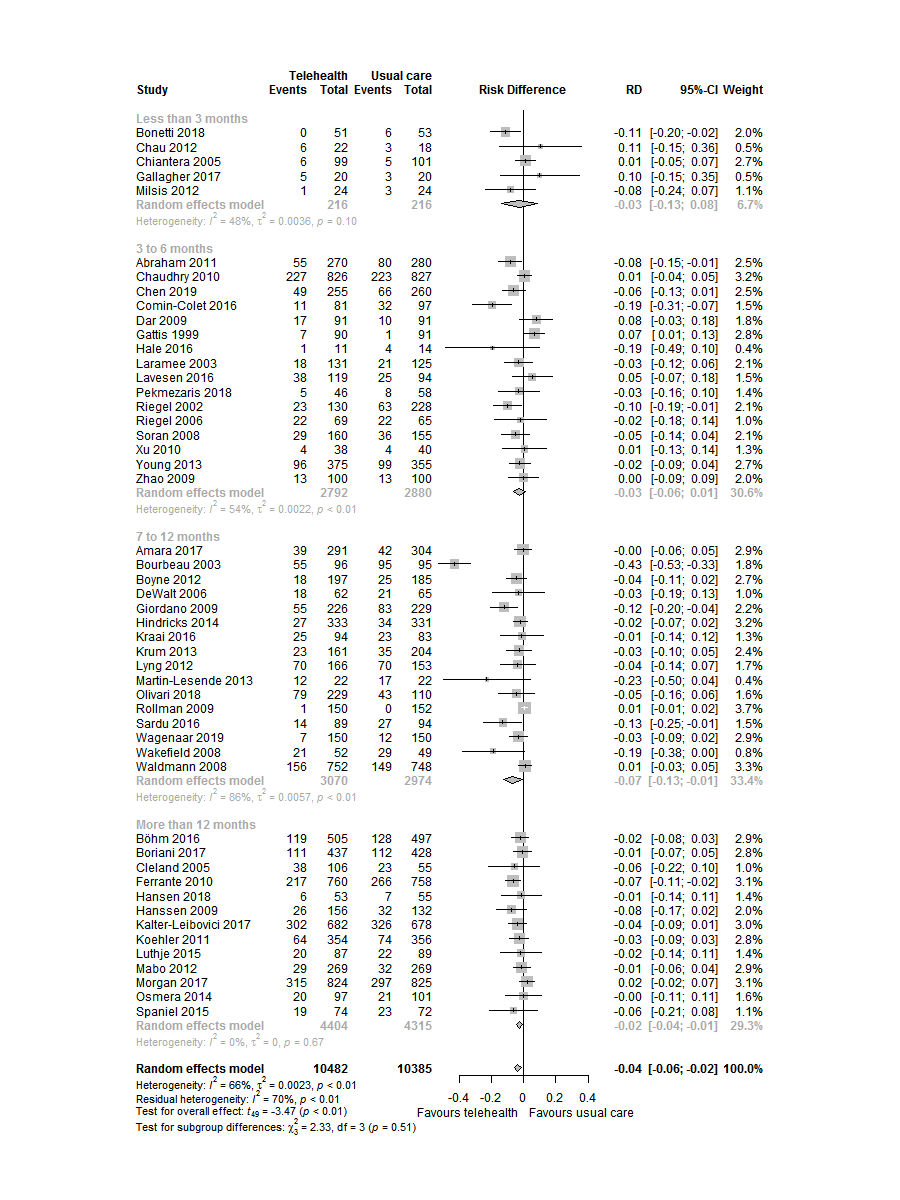


Multimedia Appendix 5 Figure 24. Forest plot of participants with a condition-related hospitalization for telehealth compared to usual care, stratified by length of follow-up


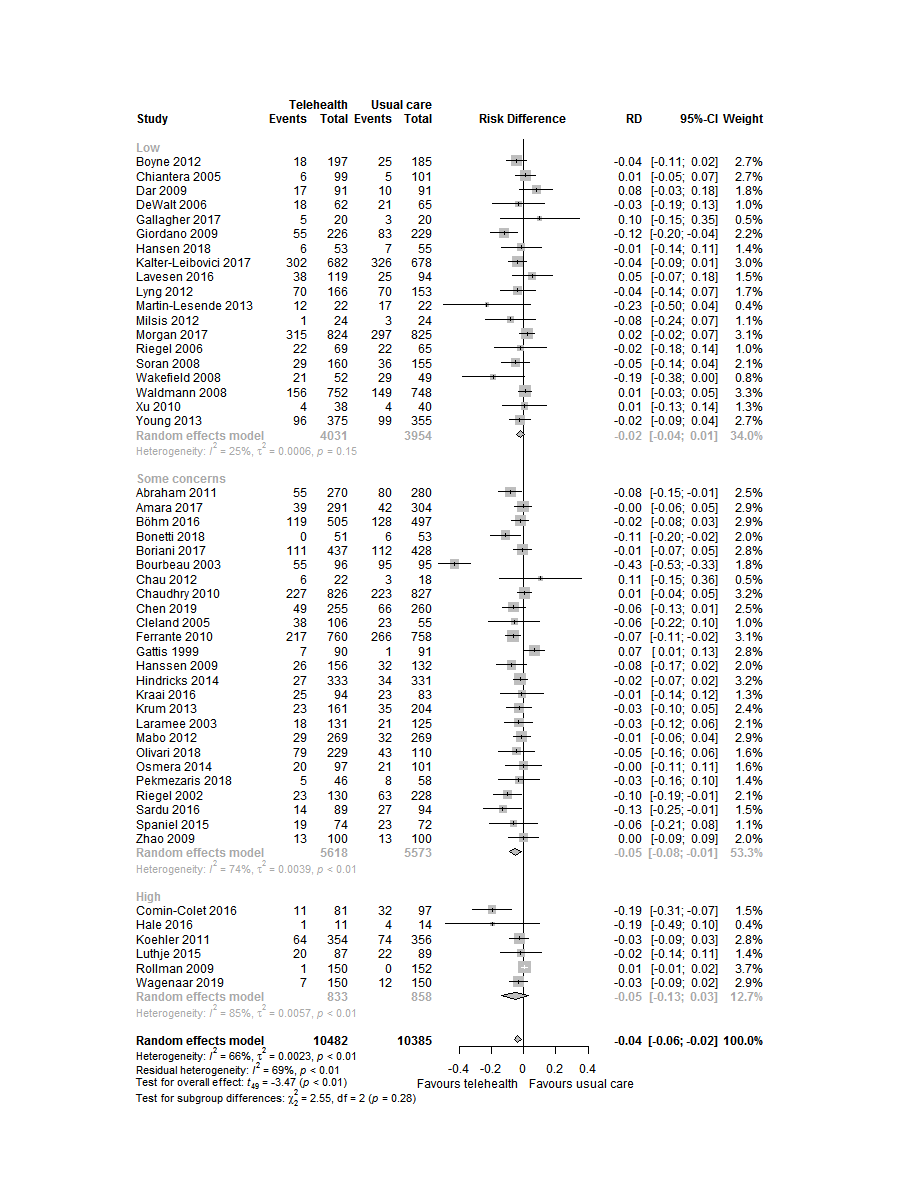


Multimedia Appendix 5 Figure 25. Forest plot of participants with a condition-related hospitalization for telehealth compared to usual care, stratified by risk of bias

Unexplained heterogeneity is below 60% for all analyses. Additionally, confidence intervals overlap largely, and variation in point estimates seems reasonable. Therefore, I do not downgrade for inconsistency.

*Risk of bias*

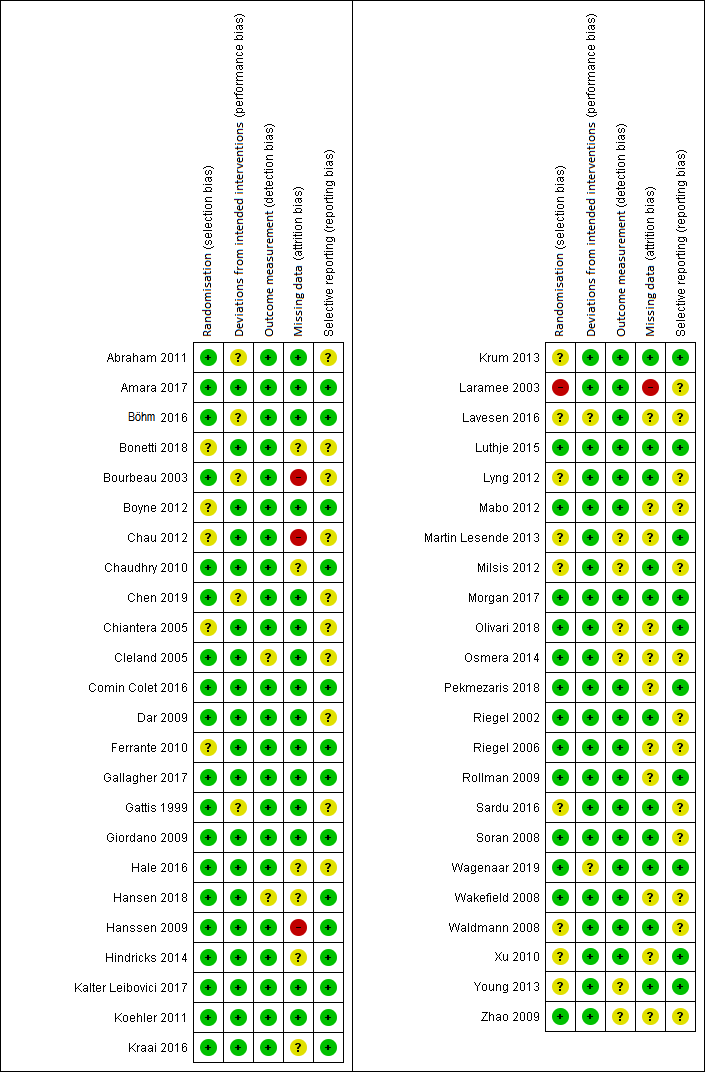


Multimedia Appendix 5 Figure 26. Risk of bias per domain per study reporting participants with a condition-related hospitalization


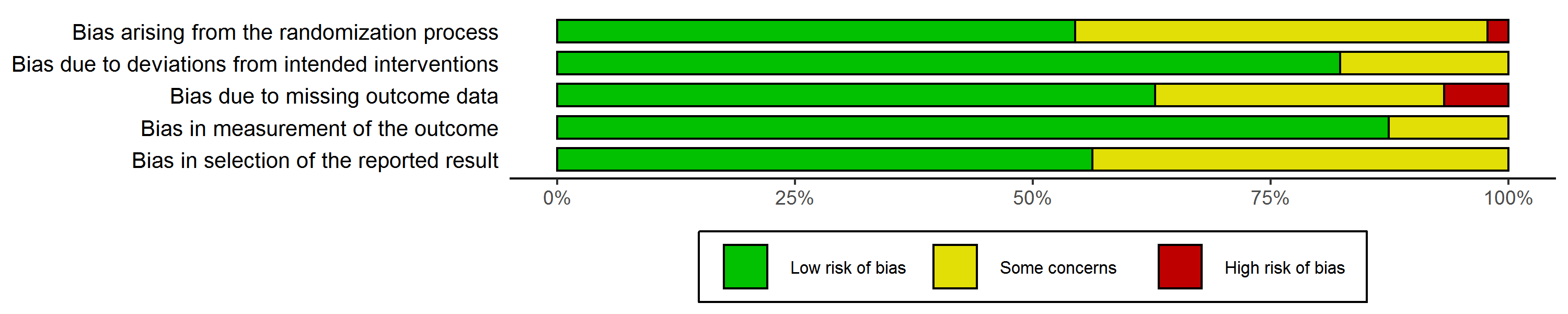


Multimedia Appendix 5 Figure 27. Weighted risk of bias summary per domain for participants with a condition-related hospitalization

Studies at a low risk of bias accounted for more than 50% of the weight in the meta-analysis in each domain. Thus, the quality of evidence is not downgraded for risk of bias.

*Imprecision*The confidence interval of the point estimate does not overlap a null effect, and a large number of participants were included. Therefore, the quality of evidence is not downgraded for imprecision.

*Publication bias*

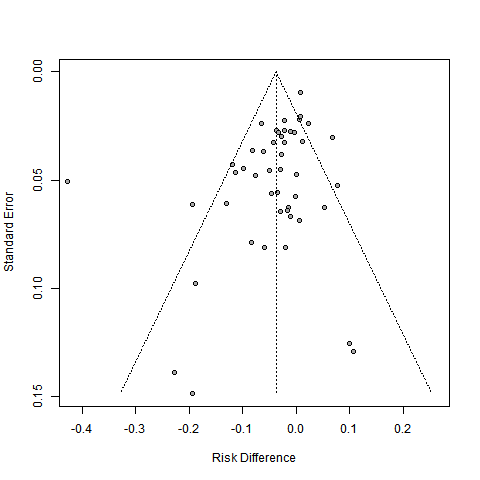


Multimedia Appendix 5 Figure 28. Funnel plot for participants with a condition-related hospitalization

The funnel plot appears to be quite symmetrical, so risk of publication bias seems small. Therefore, quality of evidence is not downgraded for risk of publication bias.

Summary: Unexplained heterogeneity is below the threshold value of 60%, imprecision is limited owing to the large number of participants, the majority of studies has a low risk of bias, and risk for publication bias appears low.
Overall judgement: High quality of evidence

**All-cause hospital days**
*Inconsistency*

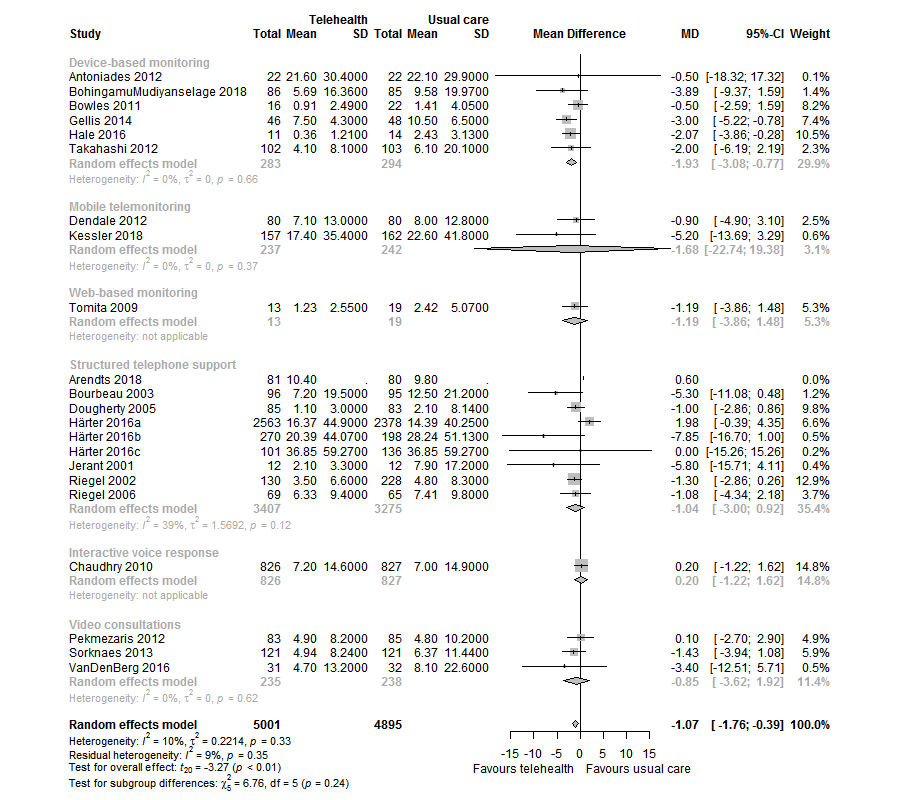


Multimedia Appendix 5 Figure 29. Forest plot for all-cause hospital days for telehealth compared to usual care, stratified by telehealth type


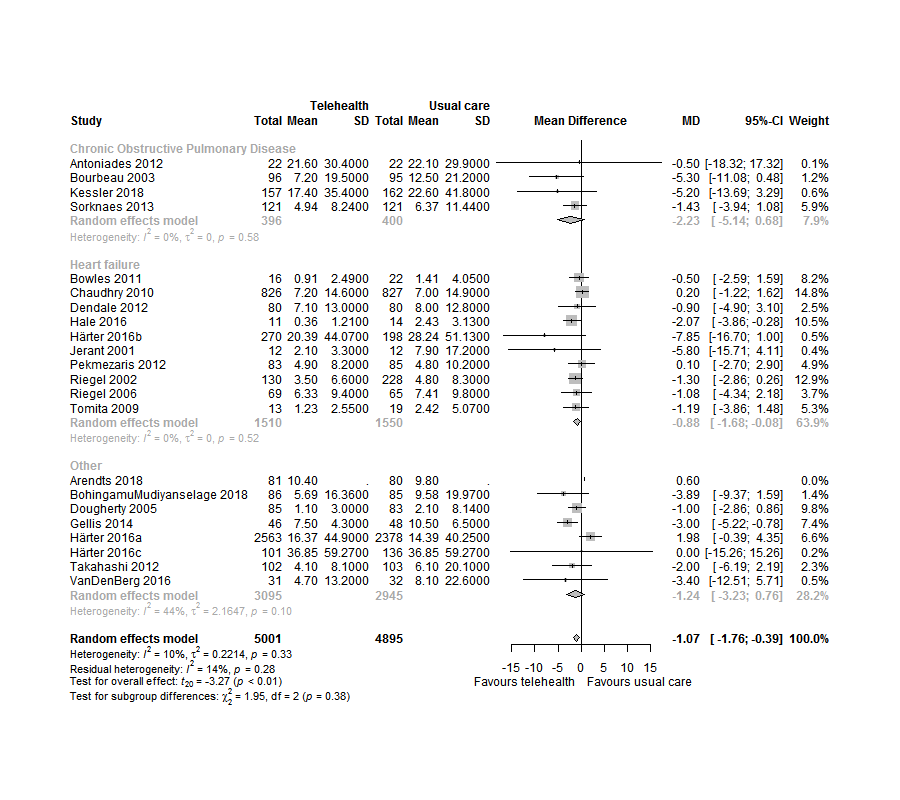


Multimedia Appendix 5 Figure 30. Forest plot for all-cause hospital days for telehealth compared to usual care, stratified by health condition


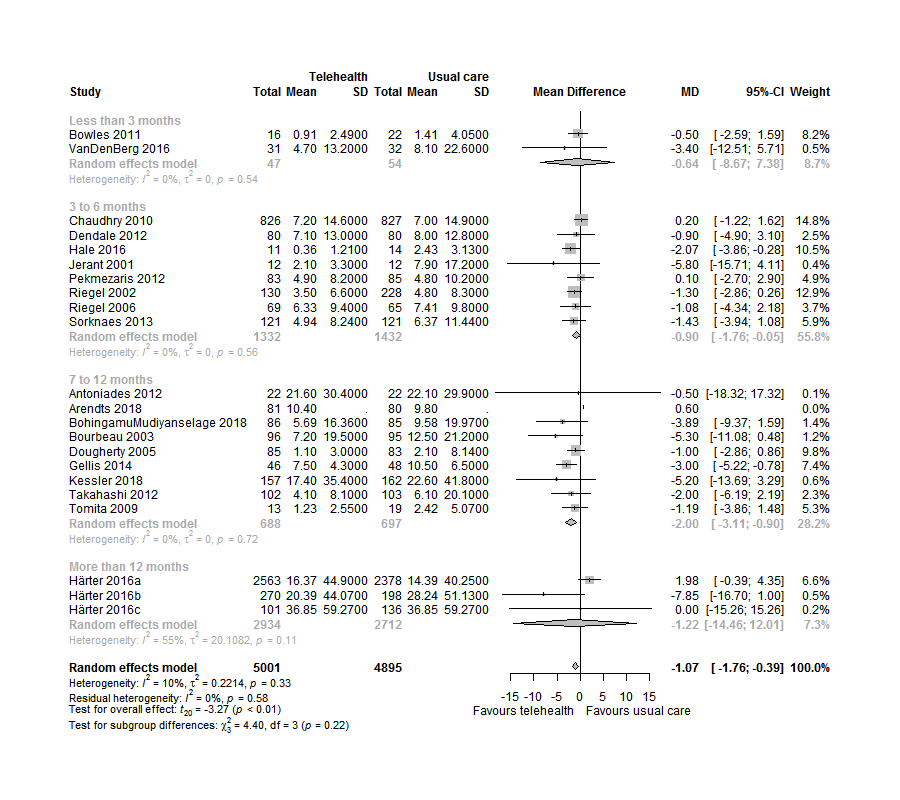


Multimedia Appendix 5 Figure 31. Forest plot for all-cause hospital days for telehealth compared to usual care, stratified by length of follow-up


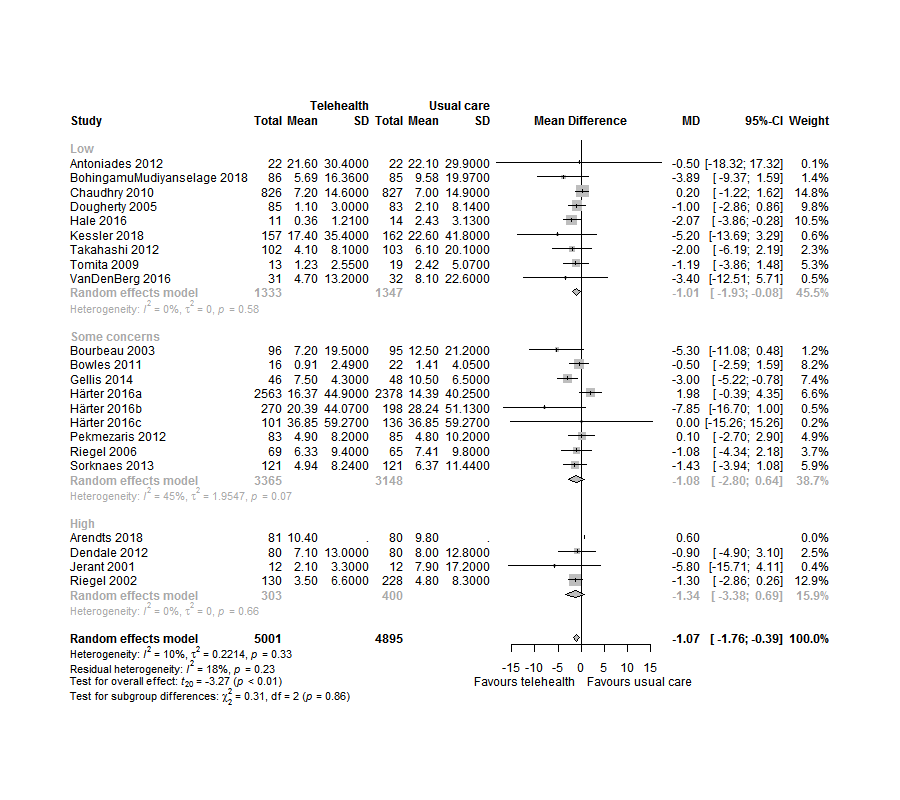


Multimedia Appendix 5 Figure 32. Forest plot for all-cause hospital days for telehealth compared to usual care, stratified by risk of bias

Residual heterogeneity is below 10%, the majority of confidence intervals overlap, and variation between point estimates seems reasonable. Therefore, quality of evidence is not downgraded for inconsistency.

*Risk of bias*
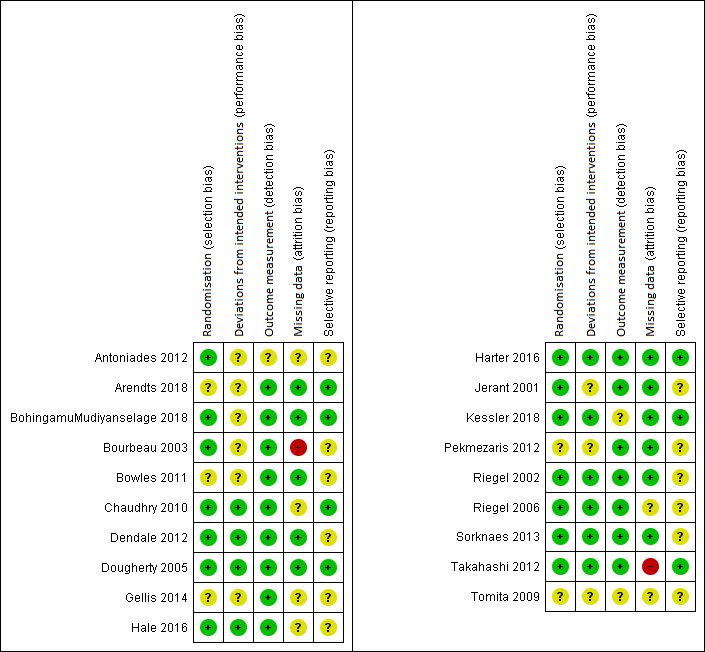


Multimedia Appendix 5 Figure 33. Risk of bias per domain per study reporting all-cause hospital days


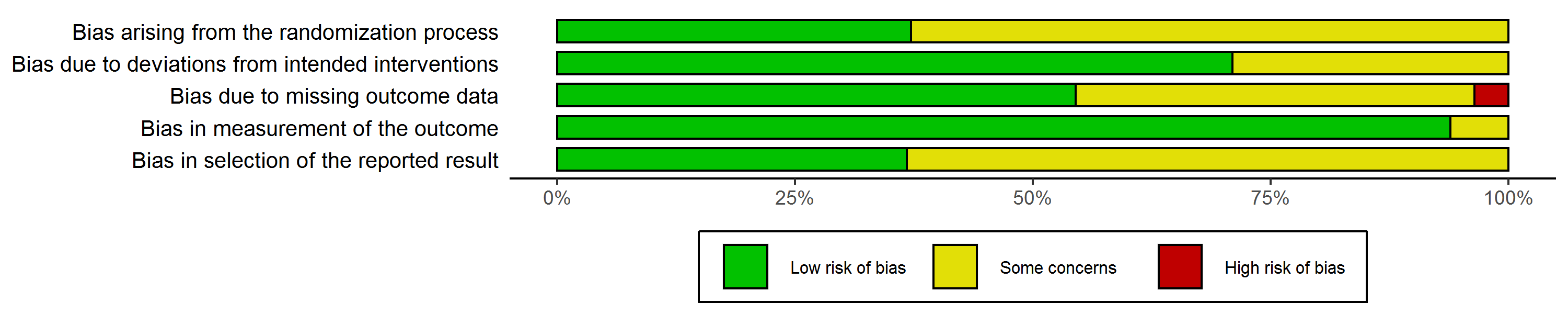


Multimedia Appendix 5 Figure 34. Weighted risk of bias summary per domain for all-cause hospital days

There was only one domain wherein studies with some concerns in terms of risk of bias accounted for more than 60% of the weight in the meta-analysis. Thus, there is no reason to downgrade the quality of evidence for risk of bias.

*Imprecision*
The confidence interval of the summary estimate does not overlap a null effect, and the analysis included well over 2000 participants, so there is no need to downgrade the quality of evidence.

*Publication bias*

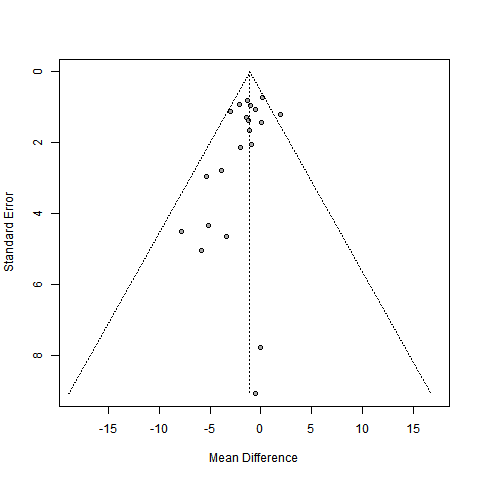


Multimedia Appendix 5 Figure 35. Funnel plot for all-cause hospital days

A limited amount of asymmetry can be observed in the funnel plot. However, as the GRADE guidelines recommend being very conservative when it comes to downgrading quality of evidence for publication bias, we consider this to be a close call, but do not downgrade the quality of evidence.

Summary: Unexplained heterogeneity is well below the threshold value of 60%, imprecision is limited owing to the large number of participants, and the majority of studies has a low risk of bias. There may be some risk of publication bias, however we do not consider this sufficiently convincing to downgrade quality of evidence.

Overall judgement: High quality of evidence

**Condition-related hospital days***Inconsistency*
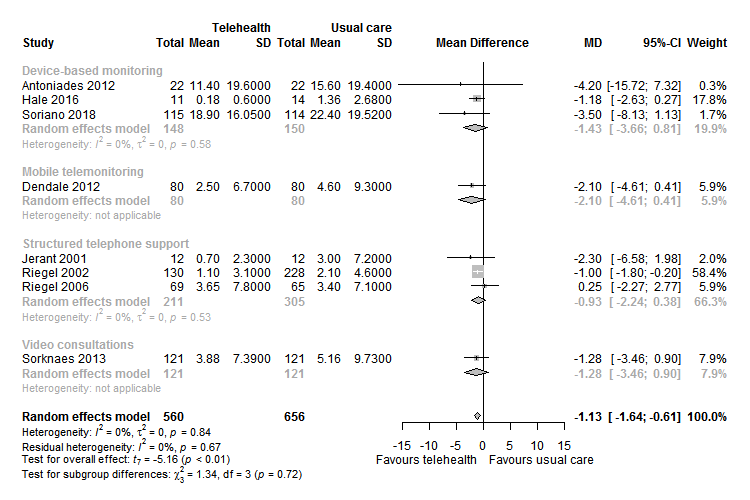


Multimedia Appendix 5 Figure 36. Forest plot for condition-related hospital days for telehealth compared to usual care, stratified by telehealth type


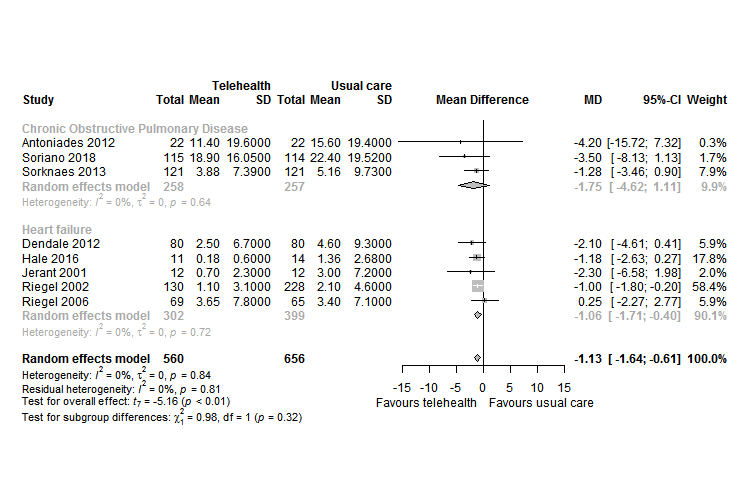


Multimedia Appendix 5 Figure 37. Forest plot for condition-related hospital days for telehealth compared to usual care, stratified by health condition


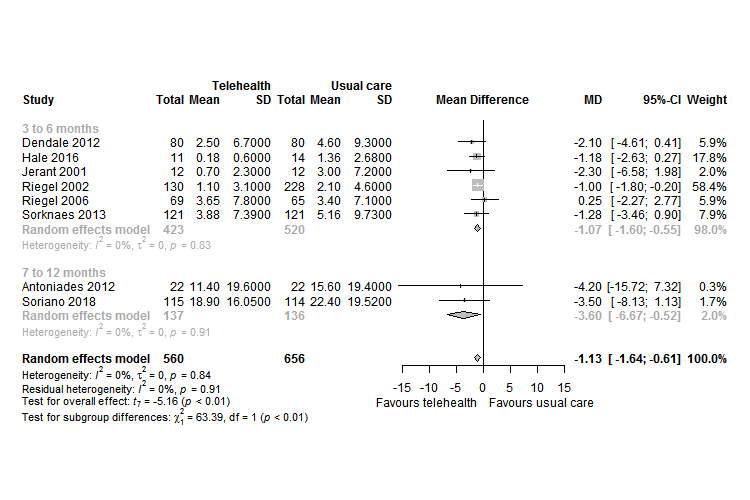


Multimedia Appendix 5 Figure 38. Forest plot for condition-related hospital days for telehealth compared to usual care, stratified by length of follow-up


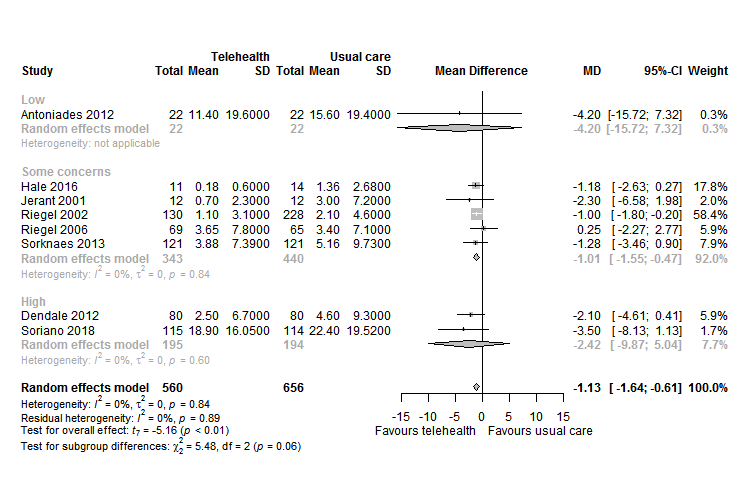


Multimedia Appendix 5 Figure 39. Forest plot for condition-related hospital days for telehealth compared to usual care, stratified by risk of bias

Each analysis shows 0% heterogeneity. Although 0% heterogeneity seems unlikely, the majority of confidence intervals appears to overlap, and variation between point estimates seems reasonable. Therefore, we do not downgrade quality of evidence for inconsistency.

*Risk of bias*

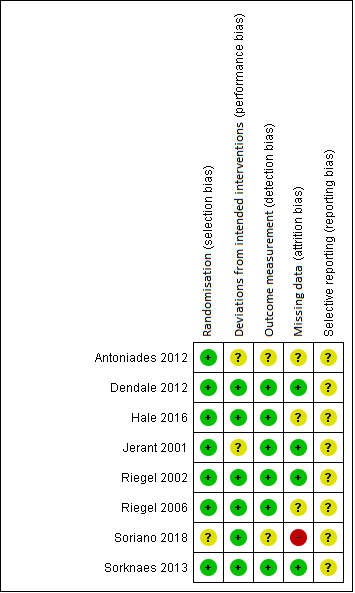


Multimedia Appendix 5 Figure 40. Risk of bias per domain per study reporting condition-related hospital days


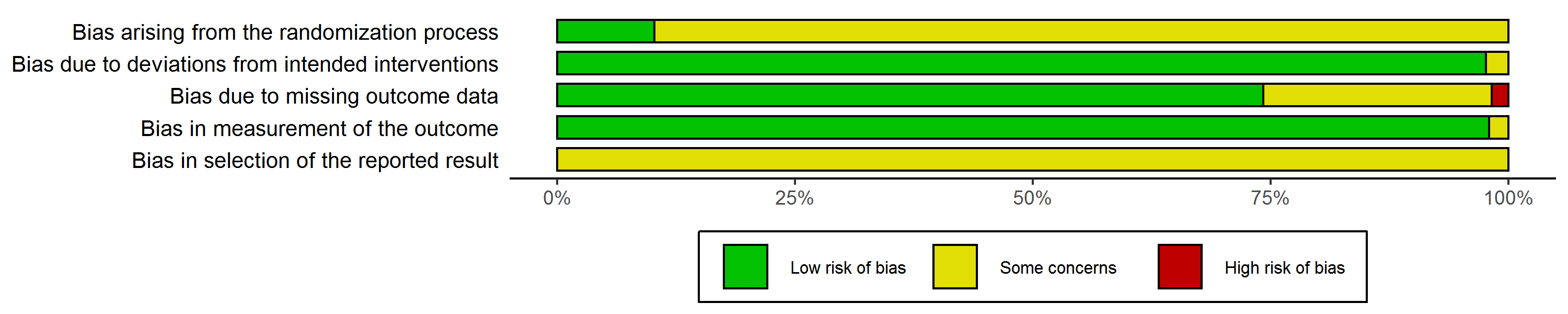


Multimedia Appendix 5 Figure 41. Weighted risk of bias summary for condition-related hospital days

More than 50% of the weight is accounted for by studies at low risk of bias in three out of the five domains. Thus, downgrading is not necessary.

*Imprecision*
Although the analysis included fewer than 2000 participants, the confidence interval does not overlap a null effect, so there does not seem to be a need to downgrade the quality of evidence.

*Publication bias*

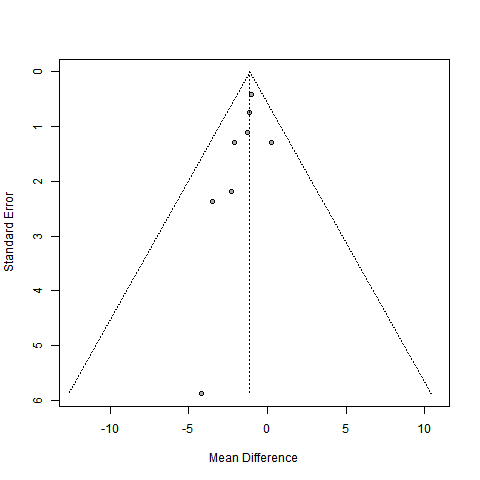


Multimedia Appendix 5 Figure 42. Funnel plot for condition-related hospital days

The funnel plot appears to be convincingly asymmetrical, which is why we downgrade the quality of evidence by 1 level for risk of publication bias.

Summary: Unexplained heterogeneity is well below the threshold value of 60%, imprecision is limited, and the majority of studies has a low risk of bias. However, we downgrade the quality of evidence by one level for risk of publication bias.

Overall judgement: Moderate quality of evidence

**Length of all-cause hospital stay***Inconsistency*
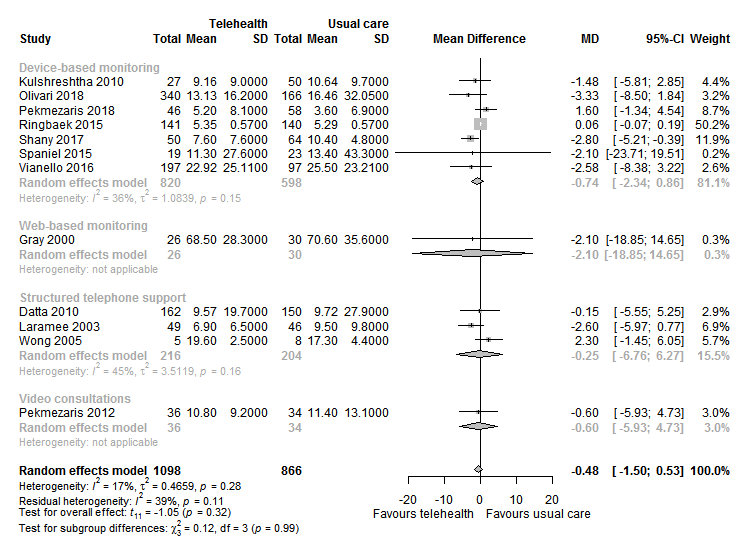


Multimedia Appendix 5 Figure 43. Forest plot for length of all-cause hospital stay for telehealth compared to usual care, stratified by telehealth type


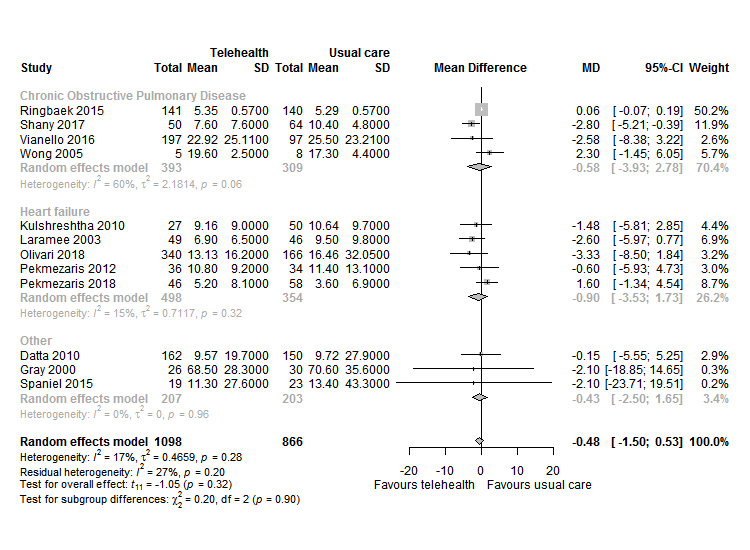


Multimedia Appendix 5 Figure 44. Forest plot for length of all-cause hospital stay for telehealth compared to usual care, stratified by health condition


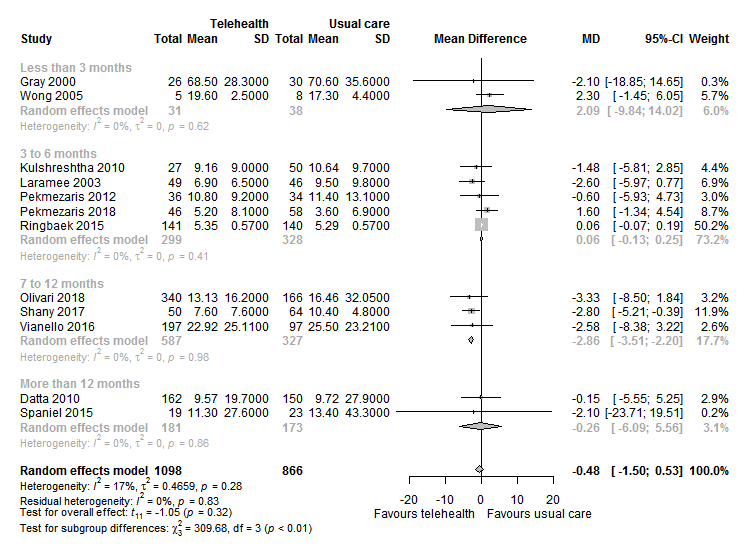


Multimedia Appendix 5 Figure 45. Forest plot for length of all-cause hospital stay for telehealth compared to usual care, stratified by health condition


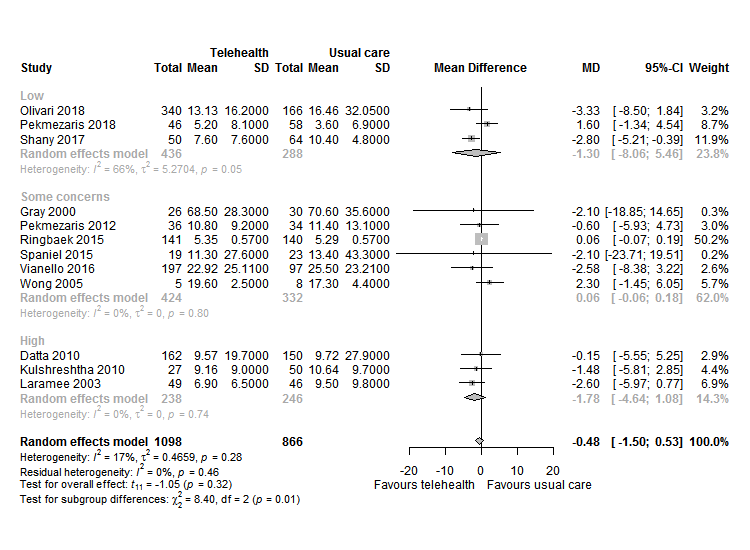


Multimedia Appendix 5 Figure 46. Forest plot for length of all-cause hospital stay for telehealth compared to usual care, stratified by risk of bias

The amount of unexplained heterogeneity is below 10% for each analysis. Furthermore, the majority of confidence intervals appears to overlap, and variation between point estimates seems limited. Therefore, there is no reason to downgrade quality of evidence for inconsistency.

*Risk of bias*

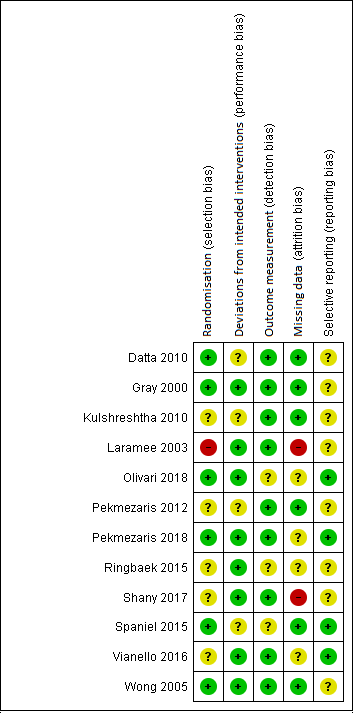


Multimedia Appendix 5 Figure 47. Risk of bias per domain per study reporting length of all-cause hospital stay


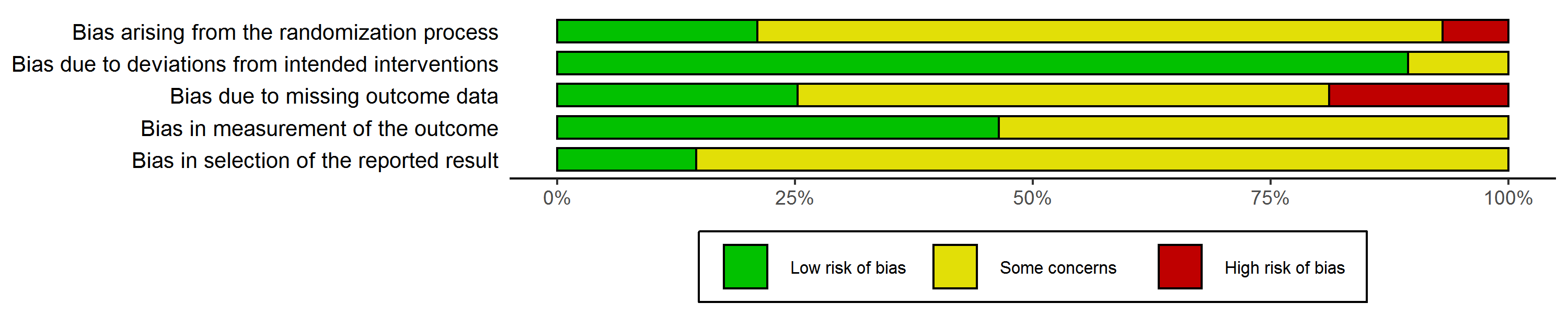


Multimedia Appendix 5 Figure 48. Weighted risk of bias summary per domain for length of all-cause hospital stay

Articles with some concerns regarding risk of bias accounted for a weight of more than 60% in 4 domains. Therefore, quality of evidence is rated down by one level for this aspect.

*Imprecision*
Because the confidence interval of the summary estimate overlaps no effect, and the analysis included less than 2000 participants, we downgrade the quality of evidence by 1 level.

*Publication bias*
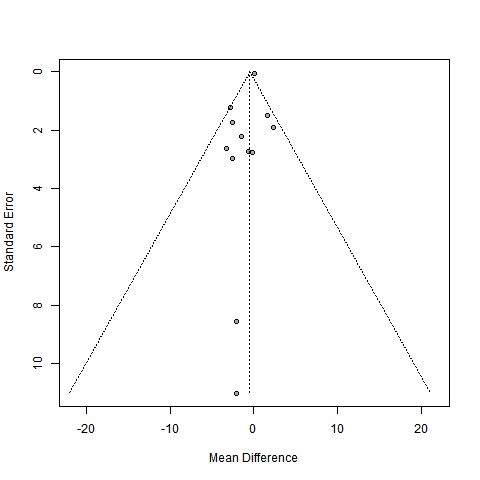


Multimedia Appendix 5 Figure 49. Funnel plot for length of all-cause hospital stay

A limited amount of asymmetry can be observed in the funnel plot. However, as the GRADE guidelines recommend being very conservative when it comes to downgrading quality of evidence for publication bias, we consider this to be a close call, but do not downgrade the quality of evidence.

Summary: Unexplained heterogeneity is well below the threshold value of 60%. We downgraded quality of evidence by one level for imprecision, as the confidence interval overlaps a null effect, and fewer than 2000 participants were included in the meta analysis. We further downgraded quality of evidence for risk of bias, because articles with some concerns regarding risk of bias accounted for more than 60% of the weight for four domains. There may be some risk of publication bias, however we do not consider this sufficiently convincing to downgrade quality of evidence.

Overall judgement: Low quality of evidence

**Length of condition-related hospital stay***Inconsistency*
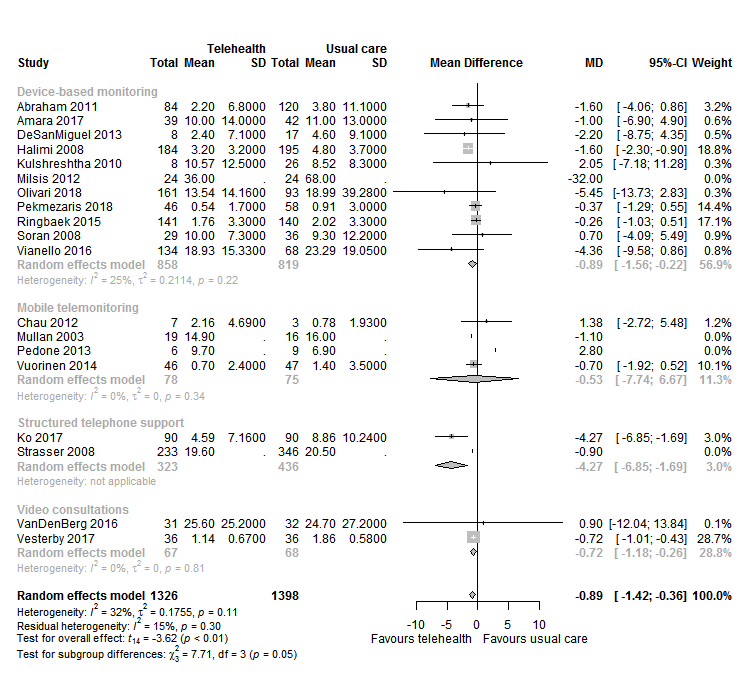


Multimedia Appendix 5 Figure 50. Forest plot for length of condition-related hospital stay for telehealth compared to usual care, stratified by telehealth type


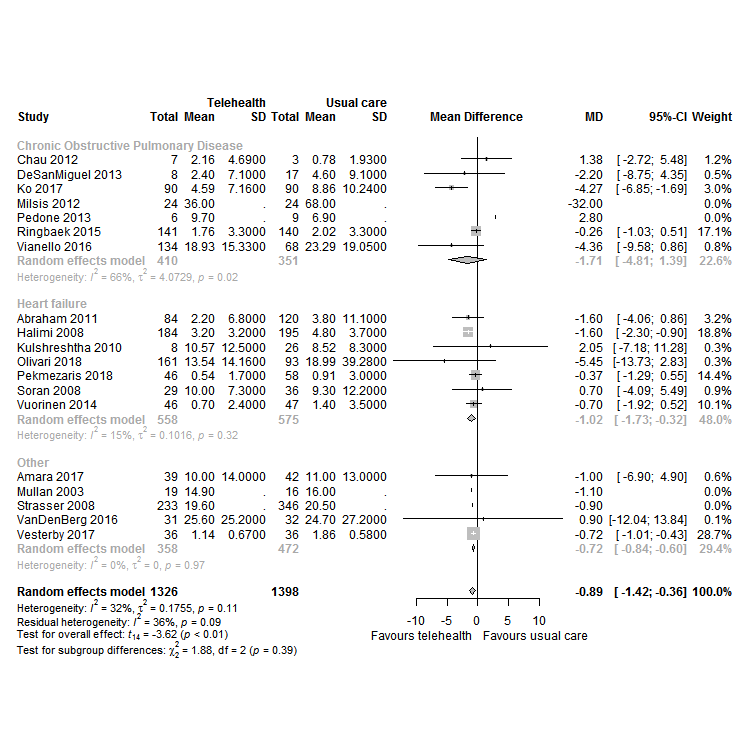


Multimedia Appendix 5 Figure 51. Forest plot for length of condition-related hospital stay for telehealth compared to usual care, stratified by health condition


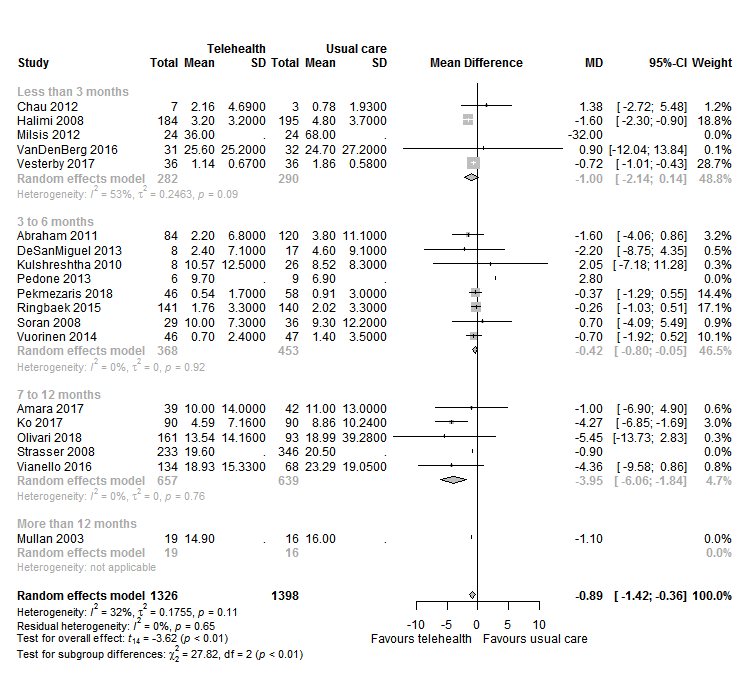


Multimedia Appendix 5 Figure 52. Forest plot for length of condition-related hospital stay for telehealth compared to usual care, stratified by length of follow-up


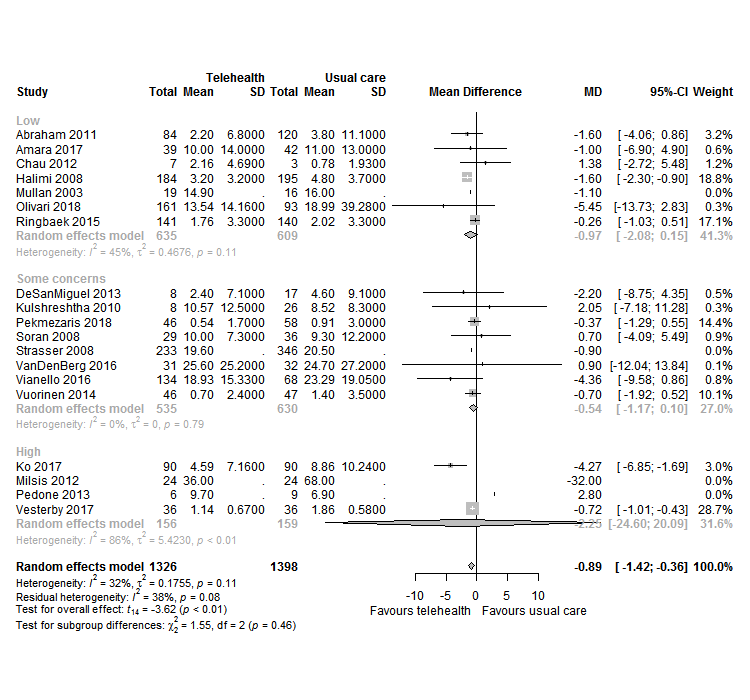


Multimedia Appendix 5 Figure 53. Forest plot for length of condition-related hospital stay for telehealth compared to usual care, stratified by risk of bias

Unexplained heterogeneity is below 15% for all analyses. Additionally, the majority of confidence intervals overlap, and variation in point estimates seems reasonable. Therefore, we do not downgrade for inconsistency.

*Risk of bias*


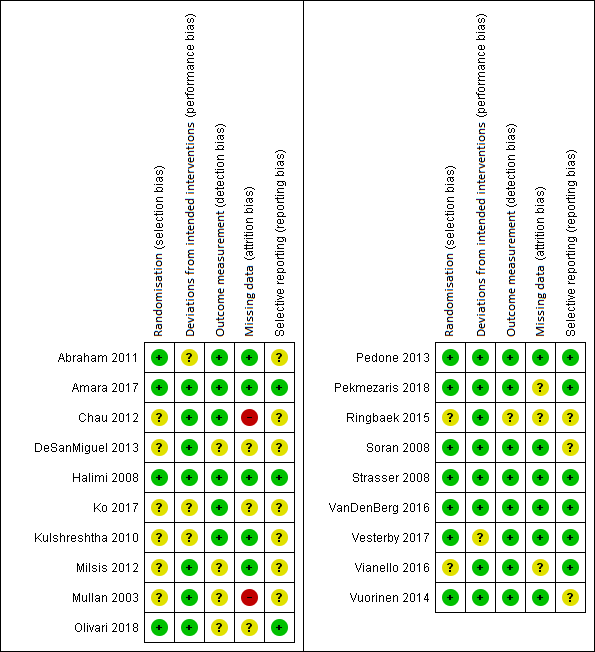


Multimedia Appendix 5 Figure 54. Risk of bias per domain per study reporting length of condition-related hospital stay


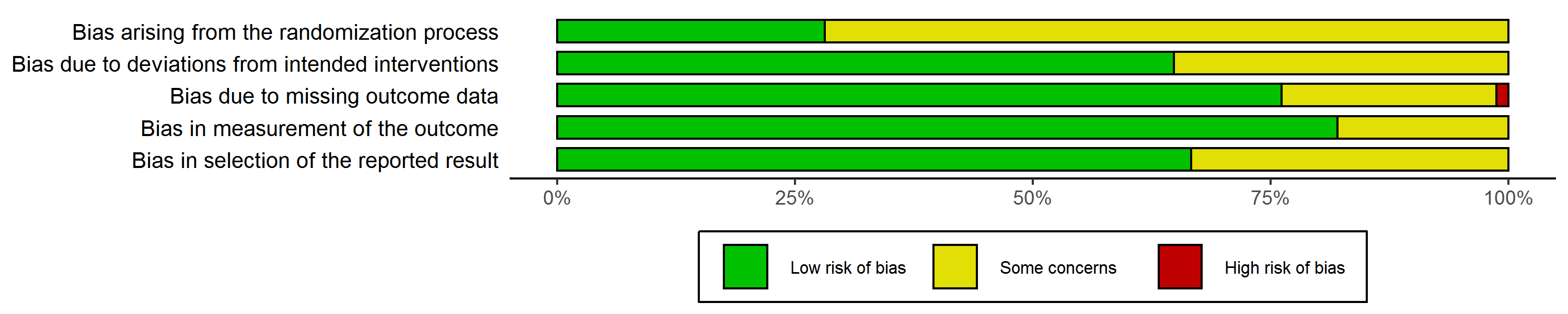


Multimedia Appendix 5 Figure 55. Weighted risk of bias summary per domain for length of condition-related hospital stay

More than 50% of the weight is accounted for by studies at low risk of bias in all domains except for randomization. Thus, we did not downgrade quality of evidence for risk of bias.

*Imprecision*
The confidence interval of the summary estimate does not overlap a null effect, and the analysis included more than 2000 participants, so there is no need to downgrade the quality of evidence for imprecision.

*Publication bias*

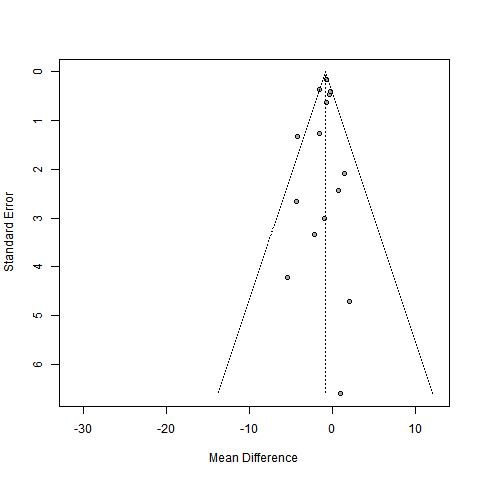


Multimedia Appendix 5 Figure 56. Funnel plot for length of condition-related hospital stay

Summary: Unexplained heterogeneity is below the threshold value of 60%, imprecision is limited owing to the large number of participants, the majority of studies has a low risk of bias, and risk for publication bias appears low.

Overall judgement: High quality of evidence
