# Supplementary material for: The Effect of Telehealth on Hospital Services Use: Systematic Review and Meta-analysis
Source: J Med Internet Res. 2021 Sep 1;23(9):e25195. doi: 10.2196/25195 (PMC8444037; doi:10.2196/25195)
Supplement: Multimedia Appendix 6 [file jmir_v23i9e25195_app6.docx]

Multimedia Appendix 6: Studies included in meta-analyses

1. Abraham WT, Adamson PB, Bourge RC, Aaron MF, Costanzo MR, Stevenson LW, et al. Wireless pulmonary artery haemodynamic monitoring in chronic heart failure: a randomised controlled trial. Lancet 2011;377(9766):658‐66.
2. Al-Sutari MM, Ahmad MM. Effect of educational program on self-care behaviors and health outcome among patients with heart failure: An experimental study. International Journal of Evidence-Based Healthcare 2017;15(4):178-85.
3. Amara W, Montagnier C, Cheggour S, Boursier M, Gully C, Barnay C, et al. Early Detection and Treatment of Atrial Arrhythmias Alleviates the Arrhythmic Burden in Paced Patients: the SETAM Study. Pacing Clin Electrophysiol 2017;40(5):527‐36.
4. Angermann CE, Störk S, Gelbrich G, Faller H, Jahns R, Frantz S, et al. Mode of action and effects of standardized collaborative disease management on mortality and morbidity in patients with systolic heart failure the interdisciplinary network for heart failure (INH) study. Circulation: Heart Failure 2012;5(1):25-35.
5. Antoniades NC, Rochford PD, Pretto JJ, Pierce RJ, Gogler J, Steinkrug J, et al. Pilot study of remote telemonitoring in COPD. Telemed J E Health 2012;18(8):634-40.
6. Arendts G, Bullow K, Etherton-Beer C, MacDonald E, Dumas S, Nagree Y, et al. A randomized-controlled trial of a patient-centred intervention in high-risk discharged older patients. European Journal of Emergency Medicine 2018;25(4):237-41.
7. Basch E, Deal AM, Kris MG, Scher HI, Hudis CA, Sabbatini P, et al. Symptom monitoring with patient-reported outcomes during routine cancer treatment: A randomized controlled trial. Journal of Clinical Oncology 2016;34(6):557-65.
8. Bekelman DB, Plomondon ME, Carey EP, Sullivan MD, Nelson KM, Hattler B, et al. Primary Results of the Patient-Centered Disease Management (PCDM) for Heart Failure Study: a Randomized Clinical Trial. JAMA Intern Med 2015;175(5):725‐32.
9. Bell SP, Schnipper JL, Goggins K, Bian A, Shintani A, Roumie C, et al. Effect of a pharmacist counseling intervention on healthcare utilization after hospital discharge: a randomized controlled trial. J Gen Intern Med 2015;30:S55‐.
10. Biese KJ, Busby-Whitehead J, Cai J, Stearns SC, Roberts E, Mihas P, et al. Telephone Follow-Up for Older Adults Discharged to Home from the Emergency Department: A Pragmatic Randomized Controlled Trial. J Am Geriatr Soc 2018;66(3):452-8.
11. Bohingamu Mudiyanselage S, Stevens J, Watts JJ, Toscano J, Kotowicz MA, Steinfort CL, et al. Personalised telehealth intervention for chronic disease management: A pilot randomised controlled trial. J Telemed Telecare 2018:1357633x18775850.
12. Böhm M, Drexler H, Oswald H, Rybak K, Bosch R, Butter C, et al. Fluid status telemedicine alerts for heart failure: A randomized controlled trial. Eur Heart J 2016;37(41):3154-63.
13. Bonetti AF, Bagatim BQ, Mendes AM, Rotta I, Reis RC, Favero MLD, et al. Impact of discharge medication counseling in the cardiology unit of a tertiary hospital in Brazil: A randomized controlled trial. Clinics (Sao Paulo) 2018;73:e325.
14. Boriani G, Da Costa A, Ricci RP, Quesada A, Favale S, Iacopino S, et al. The monitoring resynchronization dEvices and CARdiac patiEnts (MORE-CARE) randomized controlled trial: Phase 1 results on dynamics of early intervention with remote monitoring. J Med Internet Res 2013;15(8).
15. Boriani G, Da Costa A, Quesada A, Ricci RP, Favale S, Boscolo G, et al. Effects of remote monitoring on clinical outcomes and use of healthcare resources in heart failure patients with biventricular defibrillators: results of the MORE-CARE multicentre randomized controlled trial. Eur J Heart Fail 2017;19(3):416-425.
16. Bourbeau J, Julien M, Maltais F, Rouleau M, Beaupré A, Bégin R, et al. Reduction of hospital utilization in patients with chronic obstructive pulmonary disease: a disease-specific self-management intervention. Arch Intern Med 2003;163(5):585‐91.
17. Bowles KH, Holland DE, Horowitz DA. A comparison of in-person home care, home care with telephone contact and home care with telemonitoring for disease management. J Telemed Telecare 2009;15(7):344-50.
18. Bowles KH, Hanlon AL, Glick HA, Naylor MD, O'Connor M, Riegel B, et al. Clinical effectiveness, access to, and satisfaction with care using a telehomecare substitution intervention: a randomized controlled trial. Int J Telemed Appl 2011;2011:540138.
19. Boyne JJ, Vrijhoef HJ, Crijns HJ, De Weerd G, Kragten J, Gorgels AP. Tailored telemonitoring in patients with heart failure: results of a multicentre randomized controlled trial. Eur J Heart Fail 2012;14(7):791-801.
20. Braun E, Baidusi A, Alroy G, Azzam ZS. Telephone follow-up improves patients satisfaction following hospital discharge. Eur J Intern Med 2009;20(2):221-5.
21. Chau JP, Lee DT, Yu DS, Chow AY, Yu WC, Chair SY, et al. A feasibility study to investigate the acceptability and potential effectiveness of a telecare service for older people with chronic obstructive pulmonary disease. Int J Med Inform 2012;81(10):674-82.
22. Chaudhry SI, Mattera JA, Curtis JP, Spertus JA, Herrin J, Lin Z, et al. Telemonitoring in patients with heart failure. N Engl J Med 2010;363(24):2301-9.
23. Chen SH, Tsai YF, Sun CY, Wu IW, Lee CC, Wu MS. The impact of self-management support on the progression of chronic kidney disease - A prospective randomized controlled trial. Nephrology Dialysis Transplantation 2011;26(11):3560-6.
24. Chen C, Li X, Sun L, Cao S, Kang Y, Hong L, et al. Post-discharge short message service improves short-term clinical outcome and self-care behaviour in chronic heart failure. ESC Heart Fail 2019;6(1):164-73.
25. Chiantera A, Scalvini S, Pulignano G, Pugliese M, De Lio L, Mazza A, et al. Role of telecardiology in the assessment of angina in patients with recent acute coronary syndrome. J Telemed Telecare 2005;11 Suppl 1:93-4.
26. Cleland JG, Louis AA, Rigby AS, Janssens U, Balk AH. Noninvasive home telemonitoring for patients with heart failure at high risk of recurrent admission and death: the Trans-European Network-Home-Care Management System (TEN-HMS) study. J Am Coll Cardiol 2005;45(10):1654-64.
27. Comin-Colet J, Enjuanes C, Verdu-Rotellar JM, Linas A, Ruiz-Rodriguez P, Gonzalez-Robledo G, et al. Impact on clinical events and healthcare costs of adding telemedicine to multidisciplinary disease management programmes for heart failure: Results of a randomized controlled trial. J Telemed Telecare 2016;22(5):282-95.
28. Dansky KH, Vasey J, Bowles K. Impact of telehealth on clinical outcomes in patients with heart failure. Clin Nurs Res 2008;17(3):182-99.
29. Dar O, Riley J, Chapman C, Dubrey SW, Morris S, Rosen SD, et al. A randomized trial of home telemonitoring in a typical elderly heart failure population in North West London: Results of the Home-HF study. Eur J Heart Fail 2009;11(3):319-25.
30. Datta SK, Oddone EZ, Olsen MK, Orr M, McCant F, Gentry P, et al. Economic analysis of a tailored behavioral intervention to improve blood pressure control for primary care patients. Am Heart J 2010;160(2):257-63.
31. De Jong M, Van Der Meulen A, Romberg-Camps M, Becx M, Cilissen M, Maljaars J, et al. Telemedicine enables a safe shift from examination room based care to personalized care for inflammatory bowel disease: a pragmatic randomized multicenter trial with myibdcoach. Gastroenterology 2017;152(5):S186‐.
32. Dendale P, De Keulenaer G, Troisfontaines P, Weytjens C, Mullens W, Elegeert I, et al. Effect of a telemonitoring-facilitated collaboration between general practitioner and heart failure clinic on mortality and rehospitalization rates in severe heart failure: the TEMA-HF 1 (TElemonitoring in the MAnagement of Heart Failure) study. Eur J Heart Fail 2012;14(3):333-40.
33. De San Miguel K, Smith J, Lewin G. Telehealth remote monitoring for community-dwelling older adults with chronic obstructive pulmonary disease. Telemed J E Health 2013;19(9):652-7.
34. DeVito Dabbs A, Song MK, Myers BA, Li R, Hawkins RP, Pilewski JM, et al. A Randomized Controlled Trial of a Mobile Health Intervention to Promote Self-Management After Lung Transplantation. American Journal of Transplantation 2016;16(7):2172-80.
35. DeWalt DA, Malone RM, Bryant ME, Kosnar MC, Corr KE, Rothman RL, et al. A heart failure self-management program for patients of all literacy levels: a randomized, controlled trial. BMC Health Serv Res 2006;6():30.
36. Dhalla IA, O'Brien T, Morra D, Thorpe KE, Wong BM, Mehta R, et al. Effect of a postdischarge virtual ward on readmission or death for high-risk patients: a randomized clinical trial. Jama 2014;312(13):1305-12.
37. Dinesen B, Haesum LK, Soerensen N, Nielsen C, Grann O, Hejlesen O, et al. Using preventive home monitoring to reduce hospital admission rates and reduce costs: a case study of telehealth among chronic obstructive pulmonary disease patients. J Telemed Telecare 2012;18(4):221-5.
38. Domingues FB, Clausell N, Aliti GB, Dominguez DR, Rabelo ER. Education and telephone monitoring by nurses of patients with heart failure: Randomized clinical trial. Arquivos Brasileiros de Cardiologia 2011;96(3):233-9.
39. Dougherty CM, Thompson EA, Lewis FM. Long-term outcomes of a telephone intervention after an ICD. Pacing Clin Electrophysiol 2005;28(11):1157-67.
40. Dudas V, Bookwalter T, Kerr KM, Pantilat SZ. The impact of follow-up telephone calls to patients after hospitalization. Dis Mon 2002;48(4):239-48.
41. Ferrante D, Varini S, Macchia A, Soifer S, Badra R, Nul D, et al. Long-term results after a telephone intervention in chronic heart failure: DIAL (Randomized Trial of Phone Intervention in Chronic Heart Failure) follow-up. J Am Coll Cardiol 2010;56(5):372-8.
42. Finlayson K, Chang AM, Courtney MD, Edwards HE, Parker AW, Hamilton K, et al. Transitional care interventions reduce unplanned hospital readmissions in high-risk older adults. BMC Health Serv Res 2018;18(1):956.
43. Fors A, Blanck E, Ali L, Ekberg-Jansson A, Fu M, Kjellberg IL, et al. Effects of a person-centred telephone-support in patients with chronic obstructive pulmonary disease and/or chronic heart failure – A randomized controlled trial. PLoS One 2018;13(8).
44. Gallagher BD, Moise N, Haerizadeh M, Ye S, Medina V, Kronish IM. Telemonitoring Adherence to Medications in Heart Failure Patients (TEAM-HF): A Pilot Randomized Clinical Trial. J Card Fail 2017;23(4):345-9.
45. Garbutt JM, Banister C, Highstein G, Sterkel R, Epstein J, Bruns J, et al. Telephone coaching for parents of children with asthma: Impact and lessons learned. Archives of Pediatrics and Adolescent Medicine 2010;164(7):625-30.
46. Gattis WA, Hasselblad V, Whellan DJ, O'Connor CM. Reduction in heart failure events by the addition of a clinical pharmacist to the heart failure management team: results of the Pharmacist in Heart Failure Assessment Recommendation and Monitoring (PHARM) Study. Arch Intern Med 1999;159(16):1939-45.
47. Gellis ZD, Kenaley BL, Have TT. Integrated telehealth care for chronic illness and depression in geriatric home care patients: The integrated telehealth education and activation of mood (I-TEAM) study. J Am Geriatr Soc 2014;62(5):889-95.
48. Investigators G. Randomised trial of telephone intervention in chronic heart failure: DIAL trial. Bmj 2005;331(7514):425.
49. Giordano A, Scalvini S, Zanelli E, Corrà U, G.L L, Ricci VA, et al. Multicenter randomised trial on home-based telemanagement to prevent hospital readmission of patients with chronic heart failure. Int J Cardiol 2009;131(2):192-9.
50. Goodwin PJ, Segal RJ, Vallis M, Ligibel JA, Pond GR, Robidoux A, et al. Randomized trial of a telephone-based weight loss intervention in postmenopausal women with breast cancer receiving letrozole: the LISA trial. Journal of Clinical Oncology 2014;32(21):2231‐9.
51. Gray JE, Safran C, Davis RB, Pompilio-Weitzner G, Stewart JE, Zaccagnini L, et al. Baby CareLink: using the internet and telemedicine to improve care for high-risk infants. Pediatrics 2000;106(6):1318-24.
52. Hale TM, Jethwani K, Kandola MS, Saldana F, Kvedar JC. A Remote Medication Monitoring System for Chronic Heart Failure Patients to Reduce Readmissions: A Two-Arm Randomized Pilot Study. J Med Internet Res 2016;18(5):e91.
53. Halimi F, Clementy J, Attuel P, Dessenne X, Amara W. Optimized post-operative surveillance of permanent pacemakers by home monitoring: the OEDIPE trial. Europace 2008;10(12):1392-9.
54. Hannan J. APN telephone follow up to low-income first time mothers. J Clin Nurs 2013;22(1-2):262-70.
55. Hansen C, Loges C, Seidl K, Eberhardt F, Troster H, Petrov K, et al. INvestigation on Routine Follow-up in CONgestive HearT FAilure Patients with Remotely Monitored Implanted Cardioverter Defibrillators SysTems (InContact). BMC Cardiovasc Disord 2018;18(1):131.
56. Hanssen TA, Nordrehaug JE, Eide GE, Hanestad BR. Does a telephone follow-up intervention for patients discharged with acute myocardial infarction have long-term effects on health-related quality of life? A randomised controlled trial. J Clin Nurs 2009;18(9):1334-45.
57. Harrison JD, Young JM, Solomon MJ, Butow PN, Secomb R, Masya L. Randomized pilot evaluation of the supportive care intervention "CONNECT" for people following surgery for colorectal cancer. Diseases of the Colon and Rectum 2011;54(5):622-31.
58. Harter M, Dirmaier J, Dwinger S, Kriston L, Herbarth L, Siegmund-Schultze E, et al. Effectiveness of Telephone-Based Health Coaching for Patients with Chronic Conditions: A Randomised Controlled Trial. PLoS One 2016;11(9):e0161269.
59. Sisk JE, Hebert PL, Horowitz CR, McLaughlin MA, Wang JJ, Chassin MR. Effects of nurse management on the quality of heart failure care in minority communities: a randomized trial. Ann Intern Med 2006;145(4):273-83.
60. Hebert PL, Sisk JE, Wang JJ, Tuzzio L, Casabianca JM, Chassin MR, et al. Cost-effectiveness of nurse-led disease management for heart failure in an ethnically diverse urban community. Ann Intern Med 2008;149(8):540-8.
61. Hindricks G, Taborsky M, Glikson M, Heinrich U, Schumacher B, Katz A, et al. Implant-based multiparameter telemonitoring of patients with heart failure (IN-TIME): A randomised controlled trial. The Lancet 2014;384(9943):583-90.
62. Ho TW, Huang CT, Chiu HC, Ruan SY, Tsai YJ, Yu CJ, et al. Effectiveness of Telemonitoring in Patients with Chronic Obstructive Pulmonary Disease in Taiwan-A Randomized Controlled Trial. Sci Rep 2016;6:23797.
63. Imhof L, Naef R, Wallhagen MI, Schwarz J, Mahrer-Imhof R. Effects of an advanced practice nurse in-home health consultation program for community-dwelling persons aged 80 and older. J Am Geriatr Soc 2012;60(12):2223-31.
64. Ishani A, Christopher J, Palmer D, Otterness S, Clothier B, Nugent S, et al. Telehealth by an Interprofessional Team in Patients With CKD: A Randomized Controlled Trial. Am J Kidney Dis 2016;68(1):41-9.
65. Jakobsen AS, Laursen LC, Rydahl-Hansen S, Ostergaard B, Gerds TA, Emme C, et al. Home-based telehealth hospitalization for exacerbation of chronic obstructive pulmonary disease: findings from "the virtual hospital" trial. Telemed J E Health 2015;21(5):364-73.
66. Javadpour A, Hedayati A, Dehbozorgi GR, Azizi A. The impact of a simple individual psycho-education program on quality of life, rate of relapse and medication adherence in bipolar disorder patients. Asian J Psychiatr 2013;6(3):208-13.
67. Jerant AF, Azari R, Nesbitt TS. Reducing the cost of frequent hospital admissions for congestive heart failure: a randomized trial of a home telecare intervention. Med Care 2001;39(11):1234-45.
68. Jódar-Sánchez F, Ortega F, Parra C, Gómez-Suárez C, Bonachela P, Leal S, et al. Cost-utility analysis of a telehealth programme for patients with severe chronic obstructive pulmonary disease treated with long-term oxygen therapy. J Telemed Telecare 2014;20(6):307‐16.
69. Kalter-Leibovici O, Freimark D, Freedman LS, Kaufman G, Ziv A, Murad H, et al. Disease management in the treatment of patients with chronic heart failure who have universal access to health care: A randomized controlled trial. BMC Med 2017;15(1).
70. Kessler R, Casan-Clara P, Koehler D, Tognella S, Viejo JL, Dal Negro RW, et al. COMET: a multicomponent home-based disease-management programme versus routine care in severe COPD. Eur Respir J 2018;51(1).
71. Ko FWS, Cheung NK, Rainer TH, Lum C, Wong I, Hui DSC. Comprehensive care programme for patients with chronic obstructive pulmonary disease: A randomised controlled trial. Thorax 2017;72(2):122-8.
72. Koehler F, Winkler S, Schieber M, Sechtem U, Stangl K, Böhm M, et al. Impact of remote telemedical management on mortality and hospitalizations in ambulatory patients with chronic heart failure: the telemedical interventional monitoring in heart failure study. Circulation 2011;123(17):1873‐80.
73. Kraai I, de Vries A, Vermeulen K, van Deursen V, van der Wal M, de Jong R, et al. The value of telemonitoring and ICT-guided disease management in heart failure: Results from the IN TOUCH study. Int J Med Inform 2016;85(1):53-60.
74. Krum H, Forbes A, Yallop J, Driscoll A, Croucher J, Chan B, et al. Telephone support to rural and remote patients with heart failure: the Chronic Heart Failure Assessment by Telephone (CHAT) study. Cardiovasc Ther 2013;31(4):230‐7.
75. Kulshreshtha A, Kvedar JC, Goyal A, Halpern EF, Watson AJ. Use of remote monitoring to improve outcomes in patients with heart failure: A pilot trial. Int J Telemed Appl 2010.
76. Laramee AS, Levinsky SK, Sargent J, Ross R, Callas P. Case management in a heterogeneous congestive heart failure population: a randomized controlled trial. Arch Intern Med 2003;163(7):809-17.
77. Lavesen M, Ladelund S, Frederiksen AJ, Lindhardt B, Overgaard D. Nurse-initiated telephone follow-up on patients with chronic obstructive pulmonary disease improves patient empowerment, but cannot prevent readmissions. Dan Med J 2016;63(10).
78. Lindegaard Pedersen J, Pedersen PU, Damsgaard EM. Nutritional follow-up after discharge prevents readmission to hospital - A randomized clinical trial. Journal of Nutrition, Health and Aging 2017;21(1):75-82.
79. Luthje L, Vollmann D, Seegers J, Sohns C, Hasenfuss G, Zabel M. A randomized study of remote monitoring and fluid monitoring for the management of patients with implanted cardiac arrhythmia devices. Europace 2015;17(8):1276-81.
80. Lyng P, Persson H, Hgg-Martinell A, Hgglund E, Hagerman I, Langius-Eklf A, et al. Weight monitoring in patients with severe heart failure (WISH). A randomized controlled trial. Eur J Heart Fail 2012;14(4):438-44.
81. Mabo P, Victor F, Bazin P, Ahres S, Babuty D, Da Costa A, et al. A randomized trial of long-term remote monitoring of pacemaker recipients (The COMPAS trial). Eur Heart J 2012;33(9):1105-11.
82. Martin-Lesende I, Orruno E, Bilbao A, Vergara I, Cairo MC, Bayon JC, et al. Impact of telemonitoring home care patients with heart failure or chronic lung disease from primary care on healthcare resource use (the TELBIL study randomised controlled trial). BMC Health Serv Res 2013;13:118.
83. Mayo NE, Nadeau L, Ahmed S, White C, Grad R, Huang A, et al. Bridging the gap: The effectiveness of teaming a stroke coordinator with patient's personal physician on the outcome of stroke. Age Ageing 2008;37(1):32-8.
84. Milsis A, Katsaras T, Saoulis N, Varoutaki E, Vontetsianos A. Clinical effectiveness of the "healthwear" wearable system in the reduction of COPD patients' hospitalization. 2012;83 LNICST:54-60.
85. Morgan JM, Kitt S, Gill J, McComb JM, Andre Ng G, Raftery J, et al. Remotemanagement of heart failure using implantable electronic devices. Eur Heart J 2017;38(30):2352-60.
86. Olivari Z, Giacomelli S, Gubian L, Mancin S, Visentin E, Di Francesco V, et al. The effectiveness of remote monitoring of elderly patients after hospitalisation for heart failure: The renewing health European project. Int J Cardiol 2018;257:137-42.
87. Ong MK, Romano PS, Edgington S, Aronow HU, Auerbach AD, Black JT, et al. Effectiveness of Remote Patient Monitoring After Discharge of Hospitalized Patients With Heart Failure: the Better Effectiveness After Transition -- Heart Failure (BEAT-HF) Randomized Clinical Trial. JAMA Intern Med 2016;176(3):310‐8.
88. Osmera O, Bulava A. The benefits of remote monitoring in long-term care for patients with implantable cardioverter-defibrillators. Neuro Endocrinol Lett 2014;35 Suppl 1:40-8.
89. Paquette J, Le May S, Lachance Fiola J, Villeneuve E, Lapointe A, Bourgault P. A randomized clinical trial of a nurse telephone follow-up on paediatric tonsillectomy pain management and complications. J Adv Nurs 2013;69(9):2054‐65.
90. Pekmezaris R, Mitzner I, Pecinka KR, Nouryan CN, Lesser ML, Siegel M, et al. The impact of remote patient monitoring (telehealth) upon medicare beneficiaries with heart failure. Telemedicine and e-Health 2012;18(2):101-8.
91. Pekmezaris R, Nouryan CN, Schwartz R, Castillo S, Makaryus AN, Ahern D, et al. A Randomized Controlled Trial Comparing Telehealth Self-Management to Standard Outpatient Management in Underserved Black and Hispanic Patients Living with Heart Failure. Telemed J E Health 2018.
92. Phillips VL, Vesmarovich S, Hauber R, Wiggers E, Egner A. Telehealth: reaching out to newly injured spinal cord patients. Public Health Rep 2001;116 Suppl 1:94-102.
93. Pinnock H, Hanley J, McCloughan L, Todd A, Krishan A, Lewis S, et al. Effectiveness of telemonitoring integrated into existing clinical services on hospital admission for exacerbation of chronic obstructive pulmonary disease: researcher blind, multicentre, randomised controlled trial. Bmj 2013;347:f6070.
94. Riegel B, Carlson B, Kopp Z, LePetri B, Glaser D, Unger A. Effect of a standardized nurse case-management telephone intervention on resource use in patients with chronic heart failure. Arch Intern Med 2002;162(6):705-12.
95. Riegel B, Carlson B, Glaser D, Romero T. Randomized controlled trial of telephone case management in Hispanics of Mexican origin with heart failure. J Card Fail 2006;12(3):211-9.
96. Ringbaek T, Green A, Laursen LC, Frausing E, Brondum E, Ulrik CS. Effect of tele health care on exacerbations and hospital admissions in patients with chronic obstructive pulmonary disease: a randomized clinical trial. Int J Chron Obstruct Pulmon Dis 2015;10:1801-8.
97. Rollman BL, Herbeck Belnap B, LeMenager MS, Mazumdar S, Houck PR, Counihan PJ, et al. Telephone-delivered collaborative care for treating post-CABG depression: A randomized controlled trial. JAMA - Journal of the American Medical Association 2009;302(19):2095-103.
98. Sardu C, Santamaria M, Rizzo MR, Barbieri M, di Marino M, Paolisso G, et al. Telemonitoring in heart failure patients treated by cardiac resynchronisation therapy with defibrillator (CRT-D): the TELECART Study. Int J Clin Pract 2016;70(7):569-76.
99. Scherr D, Kastner P, Kollmann A, Hallas A, Auer J, Krappinger H, et al. Effect of home-based telemonitoring using mobile phone technology on the outcome of heart failure patients after an episode of acute decompensation: randomized controlled trial. J Med Internet Res 2009;11(3):e34.
100. Schwarz KA, Mion LC, Hudock D, Litman G. Telemonitoring of heart failure patients and their caregivers: a pilot randomized controlled trial. Prog Cardiovasc Nurs 2008;23(1):18-26.
101. Seto E, Leonard KJ, Cafazzo JA, Barnsley J, Masino C, Ross HJ. Mobile phone-based telemonitoring for heart failure management: a randomized controlled trial. J Med Internet Res 2012;14(1):e31.
102. Shany T, Hession M, Pryce D, Roberts M, Basilakis J, Redmond S, et al. A small-scale randomised controlled trial of home telemonitoring in patients with severe chronic obstructive pulmonary disease. J Telemed Telecare 2017;23(7):650-6.
103. Smolis-Bąk E, Dąbrowski R, Piotrowicz E, Chwyczko T, Dobraszkiewicz-Wasilewska B, Kowalik I, et al. Hospital-based and telemonitoring guided home-based training programs: effects on exercise tolerance and quality of life in patients with heart failure (NYHA class III) and cardiac resynchronization therapy. A randomized, prospective observation. Int J Cardiol 2015;199:442‐7.
104. Soran OZ, Pina IL, Lamas GA, Kelsey SF, Selzer F, Pilotte J, et al. A randomized clinical trial of the clinical effects of enhanced heart failure monitoring using a computer-based telephonic monitoring system in older minorities and women. J Card Fail 2008;14(9):711-7.
105. Soriano JB, Garcia-Rio F, Vazquez-Espinosa E, Conforto JI, Hernando-Sanz A, Lopez-Yepes L, et al. A multicentre, randomized controlled trial of telehealth for the management of COPD. Respir Med 2018;144:74‐81.
106. Sorknaes AD, Bech M, Madsen H, Titlestad IL, Hounsgaard L, Hansen-Nord M, et al. The effect of real-time teleconsultations between hospital-based nurses and patients with severe COPD discharged after an exacerbation. J Telemed Telecare 2013;19(8):466-74.
107. Spaniel F, Novak T, Bankovska Motlova L, Capkova J, Slovakova A, Trancik P, et al. Psychiatrist's adherence: a new factor in relapse prevention of schizophrenia. A randomized controlled study on relapse control through telemedicine system. J Psychiatr Ment Health Nurs 2015;22(10):811-20.
108. Steventon A, Bardsley M, Billings J, Dixon J, Doll H, Hirani S, et al. Effect of telehealth on use of secondary care and mortality: findings from the Whole System Demonstrator cluster randomised trial. Bmj 2012;344:e3874.
109. Takahashi PY, Pecina JL, Upatising B, Chaudhry R, Shah ND, Van Houten H, et al. A randomized controlled trial of telemonitoring in older adults with multiple health issues to prevent hospitalizations and emergency department visits. Arch Intern Med 2012;172(10):773-9.
110. Tomita MR, Tsai BM, Fisher NM, Kumar NA, Wilding G, Stanton K, et al. Effects of multidisciplinary internet-based program on management of heart failure. J Multidiscip Healthc 2009;2:13-21.
111. Tsuchihashi-Makaya M, Matsuo H, Kakinoki S, Takechi S, Kinugawa S, Tsutsui H. Home-based disease management program to improve psychological status in patients with heart failure in Japan. Circ J 2013;77(4):926-33.
112. Van Den Berg M, Crotty M, Liu E, Killington M, Kwakkel G, Van Wegen E. Early supported discharge by caregiver-mediated exercises and e-health support after stroke: A proof-of-concept trial. Stroke 2016;47(7):1885-92.
113. Vasilopoulou M, Papaioannou AI, Kaltsakas G, Louvaris Z, Chynkiamis N, Spetsioti S, et al. Home-based maintenance tele-rehabilitation reduces the risk for acute exacerbations of COPD, hospitalisations and emergency department visits. Eur Respir J 2017;49(5).
114. Venter A, Burns R, Hefford M, Ehrenberg N. Results of a telehealth-enabled chronic care management service to support people with long-term conditions at home. J Telemed Telecare 2012;18(3):172-5.
115. Vesterby MS, Pedersen PU, Laursen M, Mikkelsen S, Larsen J, Soballe K, et al. Telemedicine support shortens length of stay after fast-track hip replacement. Acta Orthop 2017;88(1):41-7.
116. Vianello A, Fusello M, Gubian L, Rinaldo C, Dario C, Concas A, et al. Home telemonitoring for patients with acute exacerbation of chronic obstructive pulmonary disease: a randomized controlled trial. BMC Pulm Med 2016;16(1):157.
117. Vuorinen AL, Leppanen J, Kaijanranta H, Kulju M, Helio T, van Gils M, et al. Use of home telemonitoring to support multidisciplinary care of heart failure patients in Finland: randomized controlled trial. J Med Internet Res 2014;16(12):e282.
118. Wade MJ, Desai AS, Spettell CM, Snyder AD, McGowan-Stackewicz V, Kummer PJ, et al. Telemonitoring with case management for seniors with heart failure. Am J Manag Care 2011;17(3):e71-9.
119. Wagenaar KP, Broekhuizen BDL, Jaarsma T, Kok I, Mosterd A, Willems FF, et al. Effectiveness of the European Society of Cardiology/Heart Failure Association website ‘heartfailurematters.org’ and an e-health adjusted care pathway in patients with stable heart failure: results of the ‘e-Vita HF’ randomized controlled trial. Eur J Heart Fail 2019;21(2):238‐46.
120. Wakefield BJ, Ward MM, Holman JE, Ray A, Scherubel M, Burns TL, et al. Evaluation of home telehealth following hospitalization for heart failure: a randomized trial. Telemed J E Health 2008;14(8):753-61.
121. Waldmann A, Katalinic A, Schwaab B, Richardt G, Sheikhzadeh A, Raspe H. The TeleGuard trial of additional telemedicine care in CAD patients. 2 Morbidity and mortality after 12 months. J Telemed Telecare 2008;14(1):22-6.
122. Walker PP, Pompilio PP, Zanaboni P, Bergmo TS, Prikk K, Malinovschi A, et al. Telemonitoring in chronic obstructive pulmonary disease (chromed) a randomized clinical trial. American Journal of Respiratory and Critical Care Medicine 2018;198(5):620‐8.
123. Weintraub A, Gregory D, Patel AR, Levine D, Venesy D, Perry K, et al. A multicenter randomized controlled evaluation of automated home monitoring and telephonic disease management in patients recently hospitalized for congestive heart failure: the SPAN-CHF II trial. J Card Fail 2010;16(4):285-92.
124. Wong KW, Wong FKY, Chan MF. Effects of nurse-initiated telephone follow-up on self-efficacy among patients with chronic obstructive pulmonary disease. J Adv Nurs 2005;49(2):210-22.
125. Xu C, Jackson M, Scuffham PA, Wootton R, Simpson P, Whitty J, et al. A randomized controlled trial of an interactive voice response telephone system and specialist nurse support for childhood asthma management. Journal of asthma 2010;47(7):768‐73.
126. Young JM, Butow PN, Walsh J, Durcinoska I, Dobbins TA, Rodwell L, et al. Multicenter randomized trial of centralized nurse-led telephone-based care coordination to improve outcomes after surgical resection for colorectal cancer: the CONNECT intervention. Journal of Clinical Oncology 2013;31(28):3585‐91.
127. Zhao Y, Wong FKY. Effects of a postdischarge transitional care programme for patients with coronary heart disease in China: A randomised controlled trial. J Clin Nurs 2009;18(17):2444-55.
