# Supplementary material for: The Effect of Telehealth on Hospital Services Use: Systematic Review and Meta-analysis
Source: J Med Internet Res. 2021 Sep 1;23(9):e25195. doi: 10.2196/25195 (PMC8444037; doi:10.2196/25195)
Supplement: Multimedia Appendix 7 [file jmir_v23i9e25195_app7.docx]

**Multimedia Appendix 7: Hospitalization rates**

Multimeida Appendix 7 Table 1. All-cause hospitalization rates as reported in the articles

*Telehealth*

*Usual care*

| Author / year | Rate | N | Rate | N | Rate difference (%) | Reported as | Health condition | Telehealth type |
| --- | --- | --- | --- | --- | --- | --- | --- | --- |
| Abraham 2011 | 0.32 | 270 | 0.44 | 280 | -0.12 (-27.3%) | Rate per patient per 6 months | Heart failure | Device-based monitoring |
| Boriani 2017 | 86 | 437 | 90 | 428 | -4 (-4.4%) | 2-year rate per 100 patients | Heart failure | Device-based monitoring |
| Cross 2018a | 2.7 | 116 | 11.2 | 117 | -8.5 (-75.9%) | Rate per 100 participants per year | Inflammatory bowel disease | Mobile telemonitoring |
| Cross 2018b | 0.9 | 115 | 11.2 | 117 | -10.3 (-92%) | Rate per 100 participants per year | Inflammatory bowel disease | Mobile telemonitoring |
| Dario 2017 | 0.02 | 168 | 0.04 | 78 | -0.02 (-50%) | Rate (not specified) | Type 2 diabetes | Device-based monitoring |
| Dewalt 2012 | 0.75 | 303 | 0.73 | 302 | 0.02 (+2.7%) | Rate of all-cause death or hospitalization per patientyear | Heart failure | Structured telephone support |
| Sisk 2006 | 0.74 | 203 | 0.93 | 203 | -0.19 (-20.4%) | Rate per patientyear | Heart failure | Structured telephone support |
| Ko 2017 | 1.75 | 90 | 2.59 | 90 | -0.84 (-32.4%) | Rate per patientyear | COPD | Structured telephone support |
| Tompkins 2010 | - | 193 | - | 197 | - | IRR (0.87) | Heart failure | Device-based monitoring |
| Wade 2011 | 1.21 | 164 | 1.19 | 152 | 0.02 (+1.7%) | Rate per patientyear | Heart failure | Device-based monitoring |
| Walker 2018 | 1.16 | 78 | 1.36 | 79 | -0.2 (-14.7%) | Rate per patientyear | COPD | Device-based monitoring |

Multimedia Appendix 7 Table 2. Condition-related hospitalization rates as reported in the articles

*Usual care*

*Telehealth*

| Author / year | Rate | N | Rate | N | Rate difference (%) | Reported as | Health condition | Telehealth type |
| --- | --- | --- | --- | --- | --- | --- | --- | --- |
| Böhm 2016 | 0.24 | 505 | 0.3 | 497 | -0.06 (-20%) | Rate per patientyear | Heart failure | Device-based monitoring |
| Boriani 2017 | 56 | 437 | 58 | 428 | -2 (-3.4%) | 2-year rate per 100 patients | Heart failure | Device-based monitoring |
| Cordova 2016 | 35/10951 | 34 | 44/12012 | 33 | -9 (-12.7%) | events / number of days in study | COPD | Device-based monitoring |
| Cross 2018a | 9.8 | 116 | 16.4 | 117 | -6.6 (-40.2%) | Rate per 100 patientyears | Inflammatory bowel disease | Mobile telemonitoring |
| Cross 2018b | 14.4 | 115 | 16.4 | 117 | -2 (-12.2%) | Rate per 100 patientyears | Inflammatory bowel disease | Mobile telemonitoring |
| Dario 2017 | 0.01 | 168 | 0.01 | 78 | 0 | Rate (not specified) | Type 2 diabetes | Device-based monitoring |
| Dewalt 2012 | 0.27 | 303 | 0.3 | 302 | -0.03 (-10%) | Rate per patientyear | Heart failure | Structured telephone support |
| Ko 2017 | 1.24 | 90 | 1.85 | 90 | -0.61 (-33%) | Rate per patientyear | COPD | Structured telephone support |
| Pedone 2013 | 13 | 50 | 20 | 49 | -7 (-35%) | Rate per 100 patientyears | COPD | Mobile telemonitoring |
| Phillips 2001 | 0.39 | 36 | 0.92 | 39 | -0.53 (-57.6%) | Rate per patientyear | Spinal cord injury | Video consultations |
| Wade 2011 | 1.19 | 164 | 1.16 | 152 | 0.03 (+2.6%) | Rate per patientyear | Heart failure | Device-based monitoring |
